# Genome-Wide Identification, Characterization, and Transcript Analysis of the TCP Transcription Factors in *Vitis vinifera*

**DOI:** 10.3389/fgene.2019.01276

**Published:** 2019-12-20

**Authors:** Songtao Jiu, Yan Xu, Jiyuan Wang, Lei Wang, Shiping Wang, Chao Ma, Le Guan, Muhammad Abdullah, Maoxiang Zhao, Wenping Xu, Wenli Ma, Caixi Zhang

**Affiliations:** ^1^Department of Plant Science, School of Agriculture and Biology, Shanghai Jiao Tong University, Shanghai, China; ^2^Key Laboratory of Genetics and Fruit Development, College of Horticulture, Nanjing Agricultural University, Nanjing, China; ^3^Agricultural Technology Extension and Service Center of Ningxia Agricultural Reclamation Management Bureau, Yinchuan, China

**Keywords:** genome-wide analysis, TCP gene family, phylogeny analysis, expression pattern, grapevine

## Abstract

The TEOSINTE BRANCHED 1/CYCLOIDEA/PROLIFERATING CELL FACTORS (TCP) protein, belonging to a plant-specific transcription factors (TFs) family, participates in the control of plant growth and development by regulating cell proliferation. Until now, a comprehensive study of concerning the TCP gene family and their roles in grapevine (*Vitis vinifera* L.) has not been completed. Using bioinformatics approaches, 17 *VvTCP* genes were identified and further classified into two classes, designated class I (PCF subclass) and class II (CIN and CYC/TB1 subclass), which was further supported by exon-intron organizations and conserved motif analysis. Promoter analysis demonstrated that *VvTCPs* have numerous *cis*-acting elements related to plant growth and development, phytohormone, and abiotic/biotic stress responses. The singleton duplication of grapevine *TCP* genes contributed to this gene family expansion. The syntenic analyses among *Vitis vinifera*, *Arabidopsis*, and *Oryza sativa* showed that these genes located in corresponding syntenic blocks arose before the divergence of *V. vinifera*, *Arabidopsis*, and *O. sativa*. The expression levels of 17 *VvTCPs* were determined in different tissues and fruit developmental stages, and abscisic acid (ABA) treatment. Seventeen *VvTCPs* exhibited distinct tissue-specific expression patterns, potentially illustrating the functional divergence of *VvTCPs* in all tested tissues. Eleven *VvTCPs* were down-regulated in five berry developmental stages, while three *VvTCPs* were up-regulated. Additionally, many members were strongly modulated by ABA treatment, suggesting these *VvTCPs* have important and diverse regulatory roles in ABA treatment. Our results provide valuable information on the evolution and functions of the *VvTCPs*, pave the way for further functional verification of these *VvTCPs* in grapevine.

## Introduction

The TEOSINTE BRANCHED 1/CYCLOIDEA/PROLIFERATING CELL FACTORS (TCP) gene is a plant-specific and widespread transcription factor (TF) family, which plays versatile functions in multiple biological processes, such as lateral branch (Takeda et al., 2003; Vadde et al., 2018), embryo growth (Tatematsu et al., 2008; Balsemao-Pires et al., 2013), leaf morphogenesis (Vadde et al., 2018; Mao et al., 2014), flower development (Xu et al., 2013; Juntheikki-Palovaara et al., 2014), circadian rhythm (Giraud et al., 2010), mitochondrial biogenesis (Hammani et al., 2011), seed sprout (Tatematsu et al., 2008; Rueda-Romero et al., 2012), and hormonal transduction pathways (Guo et al., 2010; Viola et al., 2011). According to the previous reports, TCP gene family is characterized as holding a basic helix-loop-helix (bHLH) motif of 59 amino acid (AA), involving in protein-protein interaction, DNA binding, and protein nuclear localization (Martin-Trillo and Cubas, 2010). TCP gene family can be divided into two subfamilies, class I (PCF subclass) and class II (CYC/TB1 and CIN subclass), on the basis of differences in AA sequences, particularly in the basic region of the TCP domains (Navaud et al., 2007; Martin-Trillo and Cubas, 2010). Compared with class II, the most significant difference is a four-amino-acid deletion of the TCP domain in class I. According to the bioinformatic analysis, some TCP members of class II also have an arginine-rich R domain outside the conservative TCP domain with unclear function, which is deemed to contribute to protein-protein interaction (Lupas et al., 1991; Cubas et al., 1999). The screening assays of DNA-binding site demonstrated that the two TCP subclasses can specifically recognize and bind to GC-rich DNA sequences with slightly different. To be specific, GGNCCCAC is the DNA binding sequence for class I, whereas class II prefers to bind to the motif G(T/C)GGNCCC (Kosugi and Ohashi, 1997; Schommer et al., 2008; Viola et al., 2011).

In general, the two class (class I and class II) genes are proven to play an antagonistical role in the specific biological process. TCP genes belonging to class I, such as *AtTCP20* and *OsPCF1*/*OsPCF2*, play a vital role in cell propagation and growth (Li et al., 2005; Martin-Trillo and Cubas, 2010; Danisman et al., 2012). In practice, single mutants of most PCF-type TCP genes do not display any noticeable phenotypic difference compared with wild type (WT) plants, which might be functional redundancies. For instance, growing evidence indicates that *AtTCP9* and *AtTCP20* function redundantly in the regulation of leaf senescence by jasmonate signaling pathway (Danisman et al., 2012). Moreover, *AtTCP14* and *AtTCP15* showed overlapping functions in repressing endoreduplication through regulating the cell-cycle gene expression to further affect the growth and development of organ and cell (Peng et al., 2015). However, double mutants of *tcp14–tcp15* showed altered sepal and blade shape, shortened internode length, as well as reduced the capability of seed germination in comparison with the WT plants (Kieffer et al., 2011; Resentini et al., 2015). Additionally, the expression levels of *CYCA1:1*, *SHOOT-MERISTEMLESS*, and *BP* in quintuple mutants of *tcp8-tcp15-tcp21-tcp22-tcp23* were up-regulated, which resulted in larger leaves (Aguilar Martinez and Sinha, 2013).

Class II can be further divided into two subclades: CYC/TB1 and CIN (Martin-Trillo and Cubas, 2010). Contrast with class I, phenotypic analysis about mutants show that the TCP genes belonging to class II usually inhibit cell growth and proliferation. In the *cin*-type mutants of *Arabidopsis* and tomato, leaf blade cells are able to continuously divide for longer period in comparison with the WT plants, and thus the mutants showed bigger leaves with altered shape and/or crinkled surface (Palatnik et al., 2003; Crawford et al., 2004; Ori et al., 2007; Efroni et al., 2008; Masuda et al., 2008). Additionally, many TCP genes of TB1-type function as axillary bud-specific regulators, such as *AtBRC1* and *AtBRC2* (Aguilar-Martinez et al., 2007), *OsFC1*/*OsTB1* (Takeda et al., 2003; Minakuchi et al., 2010), *ZmTB1* (Doebley et al., 1997), and *PsBRC1* (Braun et al., 2012). The loss-of-function of the above genes resulted in superabundant shoot branching, which are revealing of a negative function of these TCP genes on bud activity (Takeda et al., 2003; Minakuchi et al., 2010; Braun et al., 2012). Additionally, miR319a-mediated TCPs interacted with ASYMMETRIC LEAVES2 and ensured normal development of leaves *via* suppressing the expressions of *KNAT2* and *BP* (Li et al., 2012).

Recently, TCP gene family has been reported in various plants, for instance, 24 TCP genes in *Arabidopsis*, 30 in *Lycopersicon esculentum*, 28 in *Oryza sativa*, 33 in *Populus euphratica*, 36 in *Populus trichocarpa*, 27 in *Citrullus lanatus*, 19 in *Prunus mume* (Martin-Trillo and Cubas, 2010; Parapunova et al., 2014; Ma et al., 2016; Shi et al., 2016; Zhou et al., 2016). The grape is an ideal model for understanding the berry development in perennial fruit species. The availability of grapevine genome sequencing (Jaillon et al., 2007; Velasco et al., 2007) make it possible and feasible to examine and identify gene families in *Vitis vinifera*. Although TCP family genes have important regulatory roles in plant growth and development, but still lack of detail analysis in grapevine. Hence, we performed a comprehensive bioinformatics analysis and expression analysis of the TCP gene family in grapevine. The present study involved in the identification of putative *VvTCPs via* genome-wide searches, the investigation of their phylogenetic relationships, chromosomal distribution, exon-intron organizations, conserved motif, promoter, as well as syntenic analysis. In addition, the expression patterns of *VvTCPs* have been investigated by RNA sequencing data and quantitative reverse-transcription PCR (qRT-PCR) assay. The findings of the current investigations will assist in better comprehending of the classification and potential functions of *VvTCPs* and pave the way for further functional verification of these *VvTCP* genes in grapevine.

## Materials and Methods

### Plant Materials, Growth Conditions, and Abscisic Acid Treatments

The grape (the hybrid of *V. vinifera* and *Vitis labrusca* cv. “Jumeigui”) plants, planted at farm of Nanjing Agricultural University (Nanjing, China) under standard field conditions, were used as experimental materials. Three biological replicates each consisting of three clusters were sampled at each sampling date. The developmental stages were defined in term of the criterion containing the color, size, sugar content, and softening degree of the berries (Boss et al., 1996). The berry samples were harvested at five development stages, including small green berry (SGB), big green berry (BGB), veraison berry (VB), post-VB (PVB), and ripening berry (RB). Additionally, initial flowering (IF), full flowering (FF), young stems (YS), roots (Ro), buds (Bu), young leaves (YL), medium leaves (ML), old leaves (OL), and tendrils (Te) were also collected. Five-year-old uniformly grapevine plants were chosen for the ABA treatments. ABA treatments were performed by spraying the grape berry before veraison with a solution containing 50 and 150 ppm abscisic acid (ABA), while the control berries were only treated with distilled water. The berry was collected at veraison, post-veraison, and ripening. For making one sample, nine berries were collected from three different grapevines with respect to each sampling point and treatment. All of the samples were collected in triplicate, and then immediately frozen under liquid nitrogen, and kept in refrigerator at −80°C until further analysis.

### Total RNA Extraction and cDNA Library Construction

The extraction of total RNA was carried out from grapevine samples by following cetyltrimethylammonium bromide method (Jiu et al., 2016; Jiu et al., 2018). The concentration of total RNA from each sample was evaluated using NanoDrop (Thermo Fisher Scientific Inc., USA) and OD_260/280_ ratios were close to 2.0 and OD_260/230_ ratios were >2.0 for all the samples. RNA integrity was also assessed using standard denaturing agarose gel electrophoresis. The samples of total RNA were digested with DNase I (Takara, Japan) against genomic DNA contamination. Then, manufacturer's protocol was followed for construction of cDNA libraries with 1.0 g of RNA samples (PrimeScriptTM First Strand cDNA synthesis kit, Takara, Japan).

### Mining of Grape TCP Gene Family

The sequence of grapevine genome was download from National Center for Biotechnology Information website. A local BLASTn searches against the grapevine genome database using 24 known *AtTCPs* acquired by The Arabidopsis Information Resource website (http://www.arabidopsis.org), setting the threshold of the E-value as 1e−10 to confirm the detection of all *VvTCP* genes in the grape genome database (http://genomes.cribi.unipd.it/grape/index.php). Additionally, we also used the seed file of TCP domain (PF03634) from Pfam online software (http://pfam.xfam.org/) to acquire the Hidden Markov Model (HMM) sequences, then we carried out HMM searches using HMMER3 software against the grapevine protein sequences. Subsequently, each candidate *VvTCP* was used to further confirm by the Pfam database. To eliminate repetitive genes, all potential *VvTCPs* were aligned using the DNAMAN6.0 software and manually collated. All the no-overlapping *VvTCPs* were used for further analysis. The sequences of TCP family members in *C. lanatus*, *O. sativa*, *Brassica rapa* for constructing phylogenetic tree were obtained from PlantTFDB TF database (http://planttfdb.cbi.pku.edu.cn/, v3.0). The number of TCP family genes were obtained in *Arabidopsis thaliana* (Wei et al., 2016), *O. sativa* (Yao et al., 2007), *L. esculentum* (Parapunova et al., 2014), *P. mume* (Zhou et al., 2016), *C. lanatus* (Shi et al., 2016), turnip (Du et al., 2017), Chinese cabbage (Liu et al., 2018), and *Gossypium raimondii* (Ma et al., 2014).

### Multiple Sequence Alignments and Phylogenetic Analysis

Multiple sequence alignments were performed on the AA sequences of TCP proteins in *V. vinifera*, *A. thaliana*, *C. lanatus*, *O. sativa*, and *B. rapa* genomes using the CLUSTALW program with default parameters. Subsequently, MEGA7.0 program was used to construct a phylogenetic tree based on the alignments using the neighbor-joining (NJ) method and bootstrap tests replicated 1,000 time, which was performed using the *p*-distance model (Ogrodzki and Forsythe 2015). Maximum likelihood (ML) method was also used for the construction of phylogenetic tree to confirm the result from the NJ method. Additionally, the prediction of subcellular localization of all identified VvTCP genes was completed by WoLF PSORT Prediction PSORT II (http://wolfpsort.org/) and TargetP (http://www.cbs.dtu.dk/services/TargetP) software (Emanuelsson et al., 2000).

### Conserved Motifs, and Exon-Intron Organization of VvTCP Genes

The AA sequences of 17 VvTCPs were analyzed by the MEME program (http://meme-suite.org/) using the following parameters (optimum width, 15–60; number of repetitions, any; and maximum number of motifs, 15) to identify the conserved motifs (Bailey and Elkan, 1994). The conserved motifs of VvTCP proteins were confirmed by InterPro software (http://www.ebi.ac.uk/interpro/). The gene structure display server 2.0 (GSDS, http://gsds.cbi.pku.edu.cn) (Hu et al., 2014) was employed to display exon-intron organization for the 17 VvTCPs by the comparison of the cDNAs with their genomic DNA sequences.

### Putative Promoter *Cis*-Acting Element Analysis

The nucleotide sequences of VvTCP family genes were obtained from the grapevine genome database (http://genomes.cribi.unipd.it/grape/index.php) in this study. The upstream 2,000-bp region from the start codon for all *VvTCPs* was regarded as the promoter sequence (Jiu et al., 2015; Jiu et al., 2018). The putative *cis*-acting elements of promoters were identified by PlantCARE online software (http://bioinformatics.psb.ugent.be/webtools/plantcare/html/, Lescot et al., 2002). The putative *cis*-acting elements involved in plant growth and development, plant hormone responses, as well as biotic and abiotic stress responses are summarized.

### Chromosomal Localization and Collinearity Analysis

A total of 17 *VvTCPs* were mapped to grapevine chromosomes by analyzing their chromosomal localization refer to the information available at the grapevine genome database (http://genomes.cribi.unipd.it/grape/index.php). The duplication events in the grapevine genome were acquired using MCscanX software. For syntenic analysis, synteny blocks within the grapevine genome and between grapevine and *A. thaliana* genomes, as well as between grapevine and *O. sativa* genomes were obtained from the Plant Genome Duplication Database (PGDD) website (http://chibba.agtec.uga.edu/duplication/index/downloads) and visualized by Circos software (http://circos.ca/) (Krzywinski et al., 2009).

### Expression Analysis of 17 VvTCP Genes

The expression values normalized in numerous tissues, were obtained from the RNA sequencing data (Fasoli et al., 2012). The primers used for amplifying 17 *VvTCP* genes, designed by using Primer-BLAST online software (https://www.ncbi.nlm.nih.gov/tools/primer-blast/index.cgi?LINK_LOC=BlastHome), are listed in **Supplemental Table S1**. Polymerase chain reaction (PCR) were used for screening all primer pairs. The expression levels were detected by qRT-PCR assay using a Bio-Rad System (Bio-Rad, CA, USA). Each PCR mixture (20 µl) consist of 2×TB Green™ Premix Ex Taq™ II (10 µl), dilute cDNA (1 µl), each primer (0.4 µl), and RNase-free water (8.2 µl). The condition of qRT-PCR was as follows: pre-incubate (95°C for 30 s) and then 40 cycles (95°C for 5 s, 60°C for 30 s). To detect the relative fold differences for each gene in each experiment, the Ct value of the genes was normalized to the Ct value for the reference genes, and the relative expression levels were calculated using the formula 2^−ΔΔCT^. *KyActin1* was used as the internal reference controls. The lowest expression values of the samples were manually set to 1.

### Subcellular Localization

The full-length cDNA of the *VvTCP9* and *VvTCP15* were amplified using the primers (**Supplemental Table S1**) and cloned into the binary vector PHB containing two cauliflower mosaic virus (CaMV) 35S promoter, a translation enhancer and a GFP fluorescent protein tag, respectively, to generate two fusion constructs (p35S-*VvTCP9*-GFP and p35S-*VvTCP15*-GFP). After identified the sequence, the two fusion constructs and the control vector (PHB) were transformed into *Agrobacterium tumefaciens* GV3101 strains and subsequently agroinfiltrated into the leaves of 3 to 5-week-old *Nicotiana benthamiana* plants. Localization of fluorescent proteins was observed 3–7 days after infiltration, the period when GFP fluorescence was optimal, by using a confocal laser scanning microscope (Zeiss LSM 780, Germany) according to the manufacturer's instructions.

### Statistical Analysis

The experiment was arranged in a completely randomized design (CRD) with three replications in this study and collected data were statistically analyzed with multivariate analysis methods using SAS computer software (SAS Version 9.2, Institute). Analysis of variance (ANOVA) was used to determine the overall statistical significance of the data at level of *P* < 0.05 and data were represented as average ±STDEV (n = 3).

## Results

### Identification of TCP Genes in Grapevine

**Figure 1:**
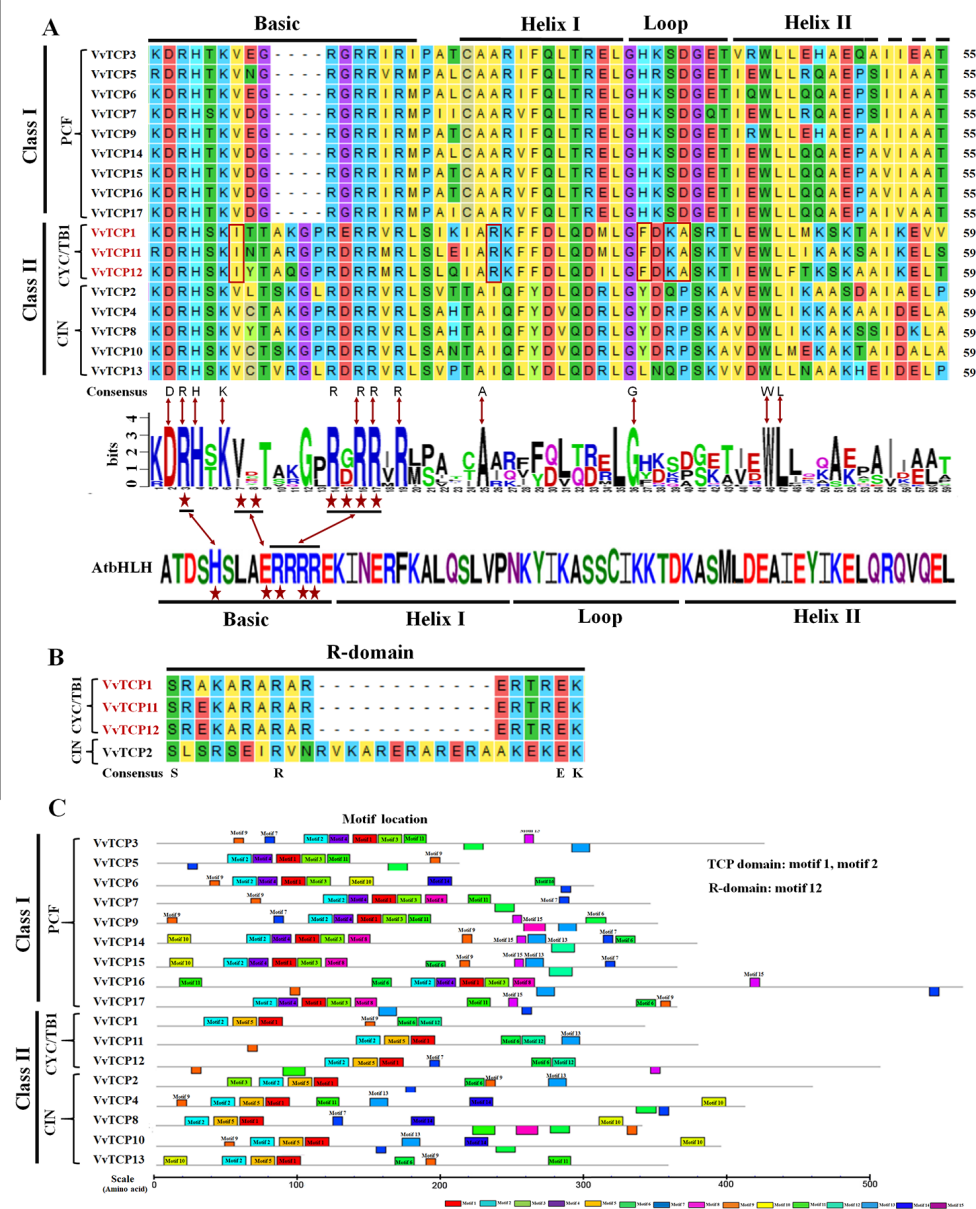
Conservative structural domains analysis of *VvTCPs* family in grapevine. **(A)** Multiple sequence alignment and protein sequence signs of the TCP domain. Multiple sequence alignment was performed with DNAMAN6.0 software. The sequence signs were constructed using WebLogo software. The stacks height indicated the sequence preservation at that position. **(B)** Multiple sequence alignment of the R domain. **(C)** Motif distribution of TCP genes in *Vitis vinifera*. Motifs were predicted using MEME online software. A colored block with motif number was consistent with each motif (color figure online). The lengths and positions of motifs in the AA sequences of 17 *VvTCPs* were exhibited by the lengths and positions of the blocks.

The availability of the grapevine genome sequences provides the sources in the genome-wide identification of grapevine genes (Jaillon et al., 2007). To identify TCP family genes in grapevine, AtTCP proteins sequences from *Arabidopsis* were used as the query for a local BLAST search against the grapevine Genome Database. A sum of 17 putative VvTCP proteins, which had the conserved TCP domain, were identified (**Table 1**). Here, we designated the *VvTCP1* to *VvTCP17* genes in grapevine according to the *Arabidopsis* TCP proteins with the highest sequence similarity and following the classification of gene terminology used in the *Arabidopsis*. The AA sequence lengths of the 17 identified VvTCP proteins ranged from 204 to 549 followed for an average of 370.65 AA. The molecular weights (Mw) of these potential VvTCP TFs ranged from 51.58 kDa (VvTCP5) to 136.89 kDa (VvTCP16), and the isoelectric points (*p*I) ranged from 4.89 to 5.10. Other characteristics of the *VvTCPs*, including the chromosome location, CIN or CYC/TB1 type, and the subcellular localization are showed in **Table 1**. The *VvTCPs* can be divided into the two TCP classes according to the differences within their TCP domains: 9 *VvTCPs* (*VvTCPs* 3, 5, 6, 7, 9, 14, 15, 16, and 17) belong to class I characterized as the existence of a four-AA-deletion in the basic domain compared with the members of class II; the 8 class II *VvTCPs* can be further clustered into the two subclasses (CYC/TB1 and CIN) (**Figure 1**).

**Table 1:** Structural and biochemical information of identified *VvTCP* members in grapevine.

Gene name^a^	Gene ID	Chromosome location	Strand	ORF (bp)	Deduced polypeptide^b^			TMD^c^	Subcellular localizations^d^	Type	Gene duplication
					Length (aa)	MW (kDa)	pI				
*VvTCP1*	VIT_214s0083g00150	chr14:22124586.22125983	Forward	993	330	81.44472	5.07	0	E.R.: 5.5, E.R._plas: 4, extr: 3, cyto: 2, mito: 2, plas: 1.5	CYC/TB1	Singleton
*VvTCP2*	VIT_210s0003g03910	chr10:6666005.6668779	Reverse	1,335	444	110.0727	5.00	0	nucl: 6, mito: 5, cyto: 2, extr: 1	CIN	Singleton
*VvTCP3*	VIT_202s0025g04590	chr2:4139943.4141512	Forward	1,236	411	102.1884	4.98	0	plas: 8, vacu: 3, cyto: 1, extr: 1, golg: 1	PCF	Singleton
*VvTCP4*	VIT_219s0014g01680	chr19:1804411.1810520	Forward	1,197	398	101.4288	4.94	0	plas: 9, vacu: 2, golg: 2, cyto: 1	CIN	Dispersed
*VvTCP5*	VIT_208s0040g01600	chr8:12723147.12724562	Reverse	615	204	51.58056	5.09	0	E.R.: 5.5, E.R._plas: 4, extr: 3, cyto: 2, mito: 2, plas: 1.5	PCF	Singleton
*VvTCP6*	VIT_214s0068g01690	chr14:25392347.25398370	Reverse	891	296	71.79306	5.10	0	nucl: 6, mito: 5, cyto: 2, extr: 1	PCF	Singleton
*VvTCP7*	VIT_210s0042g00170	chr10:12940358.12943754	Reverse	1,005	334	82.40444	5.02	0	E.R.: 5.5, E.R._plas: 4, extr: 3, cyto: 2, mito: 2, plas: 1.5	PCF	Singleton
*VvTCP8*	VIT_212s0028g02520	chr12:3280220.3283546	Forward	990	329	82.24079	5.05	0	cyto: 4.5, E.R.: 3.5, cyto_pero: 3, E.R._plas: 3, mito: 2, plas: 1.5, chlo: 1, extr: 1	PCF	Tandem
*VvTCP9*	VIT_215s0048g01150	chr15:15268234.15269562	Forward	1,020	339	84.67556	5.00	0	plas: 8, vacu: 3, cyto: 1, extr: 1, golg: 1	PCF	Singleton
*VvTCP10*	VIT_210s0003g00870	chr10:2112286.2113434	Reverse	1,149	382	96.62226	5.02	0	E.R.: 5.5, E.R._plas: 4, extr: 3, cyto: 2, mito: 2, plas: 1.5	CIN	Singleton
*VvTCP11*	VIT_217s0000g04180	chr17:4343550.4345620	Forward	1,101	366	91.26218	5.04	0	E.R.: 4.5, E.R._plas: 4, extr: 3, plas: 2.5, cyto: 2, mito: 2	CYC/ TB1	Singleton
*VvTCP12*	VIT_201s0011g02920	chr1:2574244.2576096	Reverse	1,479	492	123.2302	4.98	2	plas: 7, vacu: 4, cyto: 1, extr: 1, golg: 1	CYC/ TB1	Singleton
*VvTCP13*	VIT_214s0068g00330	chr14:24045862.24049281	Forward	1,050	349	87.8546	5.06	0	nucl: 6, mito: 5, cyto: 2, extr: 1	CIN	Singleton
*VvTCP14*	VIT_217s0000g06020	chr17:6588742.6590077	Reverse	1,107	368	92.12763	5.01	0	E.R.: 5.5, E.R._plas: 4, extr: 3, cyto: 2, mito: 2, plas: 1.5	PCF	Singleton
*VvTCP15*	VIT_201s0026g02200	chr1:11609059.11611589	Forward	1,068	355	88.21754	5.03	0	nucl: 6, mito: 5, cyto: 2, extr: 1	PCF	Singleton
*VvTCP16*	VIT_212s0035g00690	chr12:20149663.20152623	Forward	1,650	549	136.89	4.89	0	plas: 10, golg: 2, cyto: 1, vacu: 1	PCF	Dispersed
*VvTCP17*	VIT_218s0117g00340	chr18:23608849.23610082	Forward	1,068	355	88.41247	5.01	0	E.R.: 5.5, E.R._plas: 4, extr: 3, cyto: 2, mito: 2, plas: 1.5	PCF	Dispersed

a Systematic designation given to grapevine TCP genes.

b The theoretical isoelectric points (pI) and molecular weights (MW) of the deduced polypeptides were calculated using the ExPASy Compute pI/Mw tool (http://expasy.org/).

c The number of transmembrane domains was predicted by TMHMM Server v2.0.

d The subcellular localizations were predicted by WoLFPSORT. plas, plasma membrane; vacu, vacuolar membrane; chlo, chloroplast, nucl, nucleus; E.R., endoplasmatic reticulum; cyto, cytosol; golg, Golgi.

### Conserved Domains and Motif Analysis of VvTCPs in *Vitis vinifera*

The TCP domain is vital for the catalysis activity of TCP proteins. In this study, the InterPro and MEME programs were employed to confirm the conserved motifs of VvTCP proteins in *V. vinifera* (Bailey and Elkan, 1994). A total of 15 potential motifs were identified and designated motifs 1–15 in grapevine (**Supplemental Figure S1**). Motifs 1, 2, 9, and 11 were identified on the functional domains of all 17 VvTCP proteins (**Figure 1**), indicating their importance for the VvTCP proteins in grapevine. TCP domains have been expounded to involved in the dimerization and DNA binding by a bHLH-structure with usually 59 AA residues (Cubas et al., 1999). In this study, TCP domains have been identified and consisted of 55 or 59 AA residues with a bHLH-structure in all VvTCPs (**Figure 1**). In the VvTCP members of class I, the deletion of four AA was found compared with that in the VvTCP members of class II at the bHLH structure. AA sequences of VvTCPs between class I and class II were definitely different in the loop, helix I, and helix II regions, however a conserved tandem of tryptophan (W) and leucine (L) was found in helix II. The above results illuminated that TCP members belonging to different classes are supposed to have complementary functions, while those in the same class seem to display redundancies.

### Chromosomal Locations and Exon-Intron Organization of VvTCP Genes

**Figure 2:**
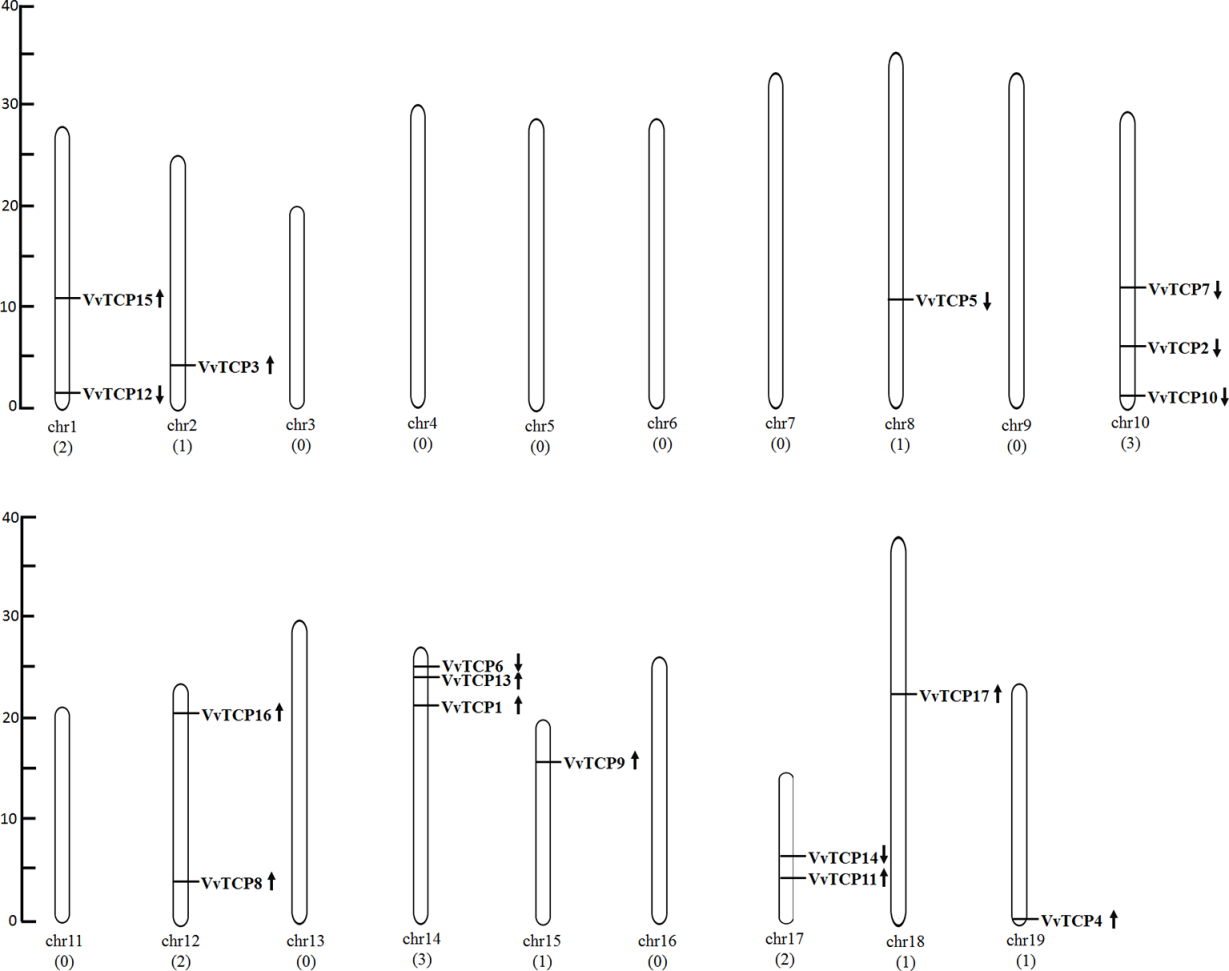
The chromosomal location of TCP genes from *Vitis vinifera*. The scale represents 40 Mb chromosomal distance. The *VvTCP* numbers are marked on the bottom of chromosomes. The *arrows* close proximity to *VvTCPs* indicated the direction of transcription. The numbers on the left side of the bars designated the approximate physical position of the first exon of corresponding *VvTCPs* on grapevine chromosomes.

**Figure 3:**
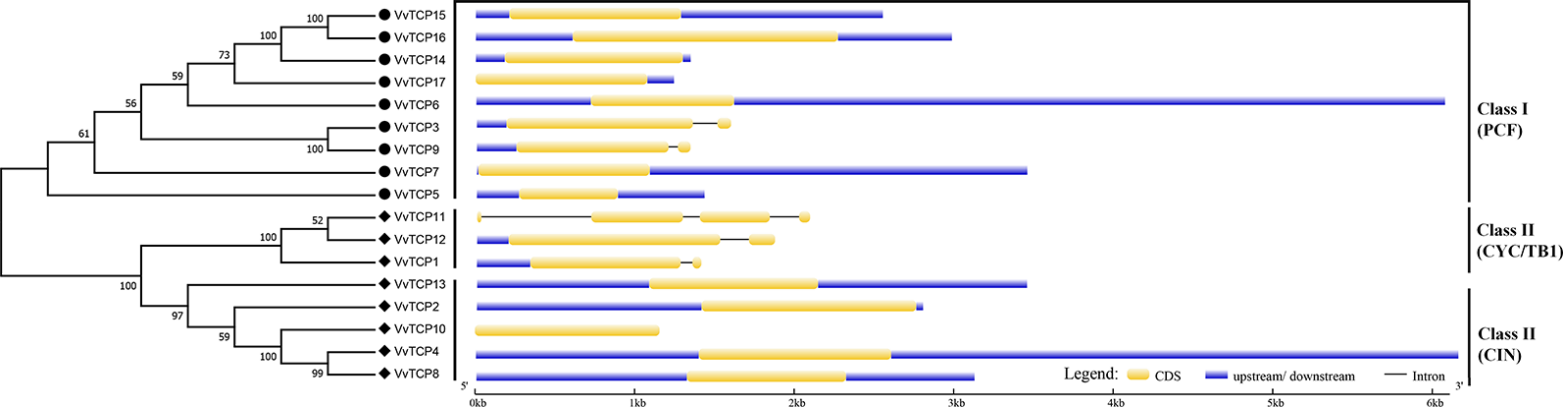
Exon-intron structure of 17 *VvTCPs* identified in *Vitis vinifera*. Shown is a graphic symbol of the gene models of all 17 *VvTCPs* identified in the present study. Exons are labeled using yellow boxes, introns are labeled using black lines, and untranslated regions are labeled using blue boxes.

**Figure 4:**
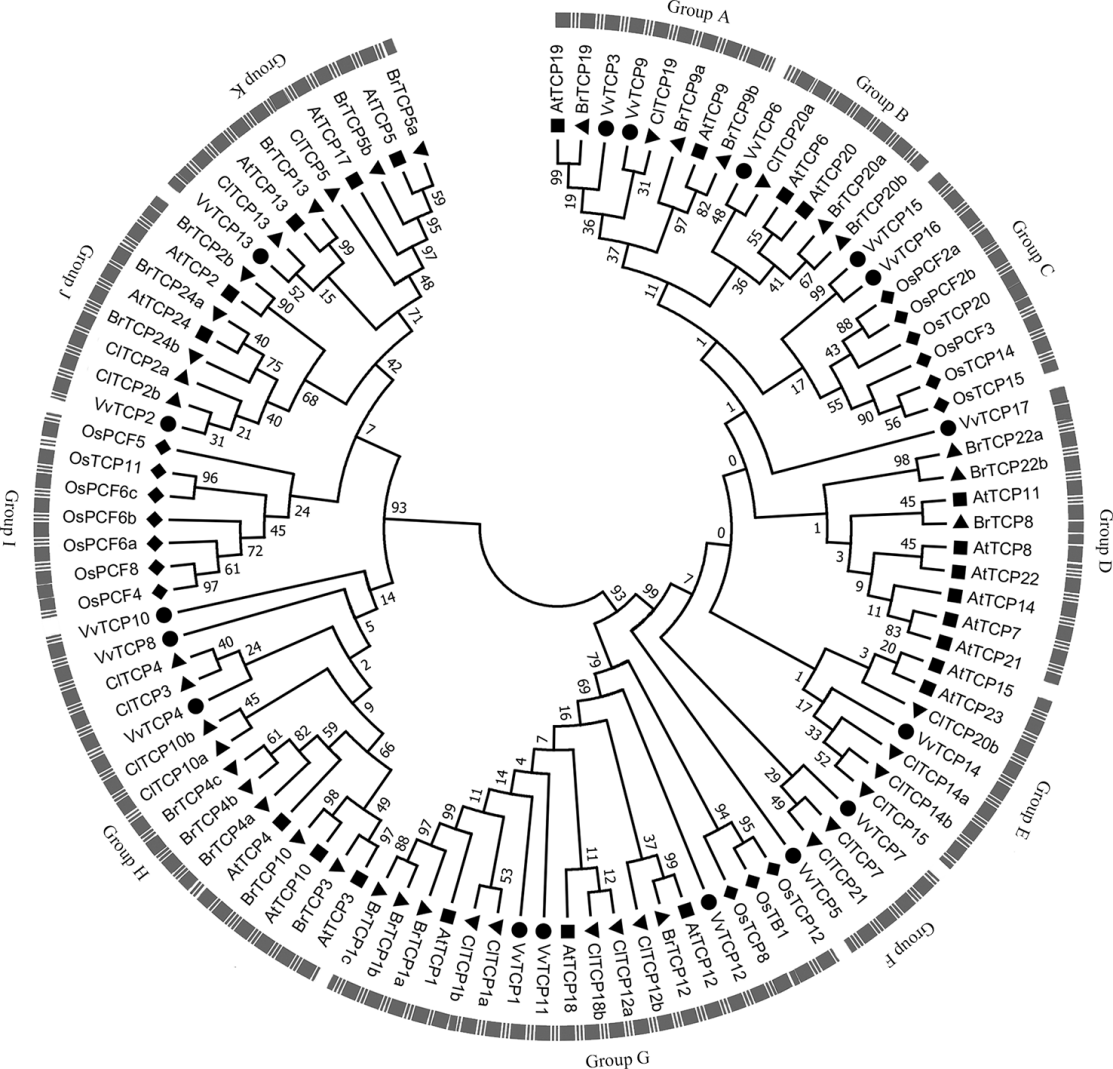
The phylogenetic analysis of TCP gene family among *Vitis vinifera*, *Arabidopsis*, *Oryza sativa*, *Citrullus Lanatus*, and *Brassica rapa*. The unrooted phylogenetic tree was constructed using the full-length protein sequences of TCPs from five species by the neighbor-joining (NJ) method with 1,000 bootstrap replicates.

The chromosomal location of each *VvTCP* in grapevine genome is shown in **Figure 2**. A total of 17 *VvTCP*s are unevenly distributed on 10 of 19 chromosomes. No genes were located on chromosomes (chrs) 3, 4, 5, 6, 7, 9, 11, 13 and 16. Whereas, one gene was located on chrs 2, 8, 15, 18, and 19, respectively. Furthermore, the chrs 1, 12, and 17 each had two genes. Meanwhile, three genes were located on chrs 10 and 14, respectively. The direction of transcription (arrows) and position of each *VvTCP* were determined on grape chromosomes available at Grape Genome Browser (Genoscope, 12× coverage). Moreover, further study displayed that the distributions of nine class I *VvTCPs* were irregular and all of them located on different chromosomes. In addition, the distribution of eight class II *VvTCPs* was also irregular and all class II *VvTCPs* located on different chromosomes except *VvTCP1* and *VvTCP13* on same chr14, as well as *VvTCP2* and *VvTCP10* on same chr10. To better comprehend the structure of *VvTCP* genes, the exon-intron organization were analyzed, which play an important role in the evolution of numerous gene families. The present study showed that most *VvTCPs* (12 out of 17) had only one exon organization, and four *VvTCP* members (*VvTCPs 1*, *3*, *9*, and *12*) contained two exons separated by one intron (**Figure 3**). Moreover, *VvTCP11* had four exons separated by three introns. Most of *VvTCP* genes possessed similar exon-intron organizations and distribution feature in the same *VvTCP* subclass, which were verified by the phylogenetic analysis (**Figure 4**).

### Phylogenetic Analysis of the VvTCP Gene Family

To better understand the phylogenetic relationships of *TCP* genes in *V. vinifera*, *Arabidopsis*, *O. sativa*, *C. lanatus*, and *B. rapa*, a NJ phylogenetic tree was created based on multiple sequence alignment of 17 *VvTCPs*, 23 *AtTCPs*, 16 *OsTCPs*, 21 *ClTCPs*, and 23 *BrTCPs* using MEGA 7.0 software (**Figure 4**). The previous reports showed that bootstrap values for some nodes of the ML phylogenetic tree were low because of quite large number of *TCP* genes (Yao et al., 2007; Martin-Trillo and Cubas, 2010). Therefore, we also constructed ML phylogenetic tree and further sought extra evidences to validate the dependability of our phylogenetic tree. The phylogenetic trees constructed by NJ and ML methods mentioned above were almost identical with only tiny differences at some branches, indicating that the two methods were largely identify with one another. In addition, the analysis of conservative domains and motif and gene structure also further verified the rationality of our phylogenetic trees. In view of the great similarity among these tree topologies exactly as previous reports (Yao et al., 2007; Martin-Trillo and Cubas, 2010), the NJ phylogenetic tree was selected for deep analysis. According to the NJ phylogenetic tree, the *TCP* genes family was divided into 11 categories designated as group A to group K (**Figure 4**). Based on their sequence structures both outside and within the TCP domain, *TCPs* in group G belonged to the class II subclass CYC/TB1-type, groups H, I, J, and K belonged to the class II subclass CIN-type, whereas *TCPs* in the remaining groups A–F belonged to class I subclass PCF-type. Group G, the largest clade among all groups, had 18 TCP memberships, holding 18.0% of the overall *TCP* genes; while group H is the second largest clade, consisting of 15 TCP members. The smallest clade is group F, which contains only four memberships. In general, the *TCP* genes displayed a scattered distribution in the most of groups, suggesting that the expansion of *TCP* gene family have been performed before the lineage evolutionary divergence times. It is interesting to note that group I only contained seven TCP members of *O. sativa*. Meanwhile, the *O. sativa* TCP genes were absent in several groups, such as groups A, B, D, E, F, H, J, and K.

### Identification of the Putative *Cis*-Elements in the Promoter of Grapevine TCP Genes

The evaluation of *cis*-elements in promoters is a key factor for comprehending transcriptional regulation and gene function. To recognize the putative *cis*-regulatory elements of the *VvTCP*s, the promoter sequences of the 17 *VvTCPs* were searched in the *Vitis* genomic database (http://genomes.cribi.unipd.it/grape/index.php). In the present study, a 2-kb was considered as a promoter region for 16 *VvTCPs*, whereas, a <2 kb promoter sequence for the *VvTCP3* was also identified because of the existence of another gene situated at the <2 kb upstream (**Supplemental Data Set 1**). A total of 92 putative *cis*-elements involved in plant hormones [e.g., auxin, ABA, methyl jasmonate (MeJA), ethylene, gibberellin (GA), and salicylic acid (SA)] responses, biotic and abiotic stress response, and plant growth and development were identified using PlantCARE online database (**Supplementary Table S2**). As shown in **Supplemental Table S2**, the predicted *cis*-elements differed among the 17 *VvTCPs*, and two *cis*-elements (CAAT-box and TATA-box) were the most abundant, which had the largest number in all 17 *VvTCPs*. It is quite interesting to recognize that these unique *cis*-elements (AuxRR-core, MBSI, RY-element, circadian, etc.) were only present in one of 17 *VvTCPs* implying that these gene-specific *cis*-elements might play the special role in the regulating some biological process.

**Figure 5:**
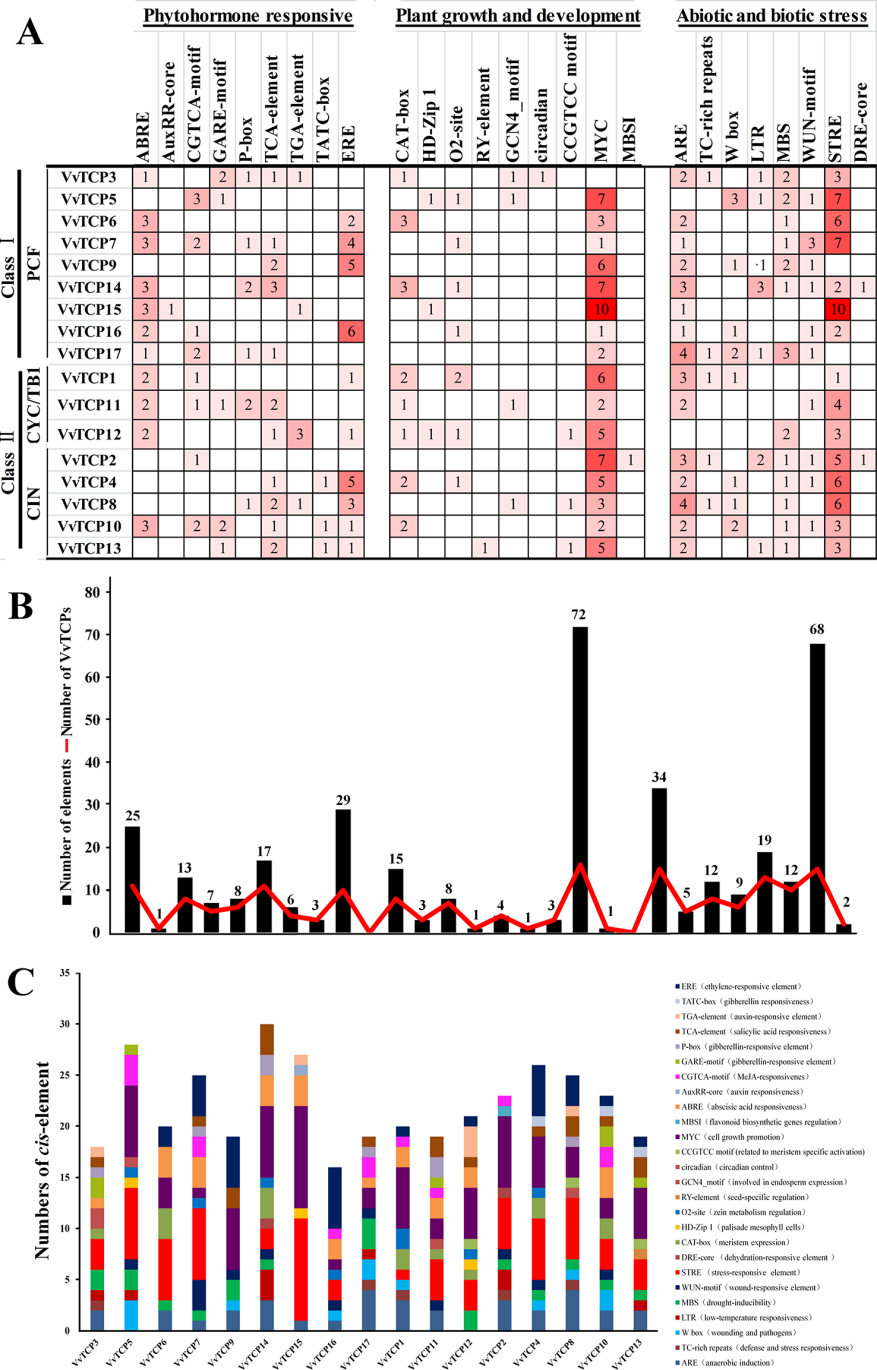
*Cis*-regulatory element analysis of the promoter regions of VvTCP genes in grapevine. **(A)** Number of every *cis*-regulatory element in the promoter region of *VvTCPs*. **(B)** Statistics for the overall number of *VvTCPs*, together with the corresponding *cis*-regulatory elements (red dot) and the overall number of *cis*-regulatory elements in VvTCP gene family grapevine (black box). Based on the functional annotation, the *cis*-regulatory elements were divided into three main categories: phytohormone responsive, plant growth and development, abiotic and biotic stresses-related *cis*-regulatory elements (detailed results shown in **Supplementary Table S2**). **(C)** Number of *cis*-acting elements in the promoter of VvTCPs genes that are related to stress responses and plant growth and development.

As shown in **Figure 5**, some phytohormone-related *cis*-regulatory elements including the ABA response element (ABRE), the MeJA response element (CGTCA motif), the SA-responsive element (TCA element), the ethylene-responsive element (ERE) and the auxin-responsive element (AuxRR-core and TGA-element) and the GA-responsive element (TATC-box, GARE motif, and P-box) were found in the promoters of 11, 8, 11, 10, 5, and 14 *VvTCPs*, respectively. A mass of phytohormone-responsive elements were recognized in the *VvTCP* promoters, suggesting they could play an important role in regulating the growth and development in plants (**Figure 5**). In several *cis*-elements related to plant growth and development, the circadian regulator element (circadian), the leaf development-related *cis*-regulatory element (HD-Zip1), the zein-metabolism regulation element (O_2_ site), the seed-specific regulation element (RY element), the meristem expression element (CAT-box), the meristem specific activation element (CCGTCC-box), and the endosperm-specific expression element (GCN4_motif) were identified in the promoters of 1, 3, 7, 1, 8, 3, and 4 *VvTCPs*, respectively. Additionally, some stress-related (e.g., low-temperatures, drought, and pathogens) *cis*-regulatory elements were also observed in the putative promoter sequences of the *VvTCPs* (**Figure 5**).

### Gene Duplication and Syntenic Analysis of the VvTCP Genes

For the gene family expansion and evolution of new functions, gene duplication and divergence are essential steps in the plant genome. *Vitis* spp. has suffered from whole-genome duplications (WGD) during its generative history (Jaillon et al., 2007). Moreover, several gene duplication events including tandem duplication, WGD/segmental, and rearrangements at the chromosomal and gene level, drive the evolution of gene families encoding proteins (Maher et al., 2006). To evaluate the effect of duplications on the *VvTCPs* family, we firstly analyzed the origins of duplicate genes for the *VvTCPs* family in the grapevine genome utilizing the MCScanX software. Each member of the *VvTCPs* was allocated to one of five duplication events (dispersed, singleton, tandem, proximal, and WGD/segmental). The results showed that 76.47% (13) of the VvTCPs in grapevine were duplicated from singleton event, compared with only 17.65% (3) from dispersed, and 5.88% (1) from tandem event (**Table 1**). However, no WGD/segmental and proximal duplication events were found in the grapevine VvTCP genes. The results demonstrated that singleton duplication played a vital role in the expansion of the *VvTCPs* family in grapevine.

**Figure 6:**
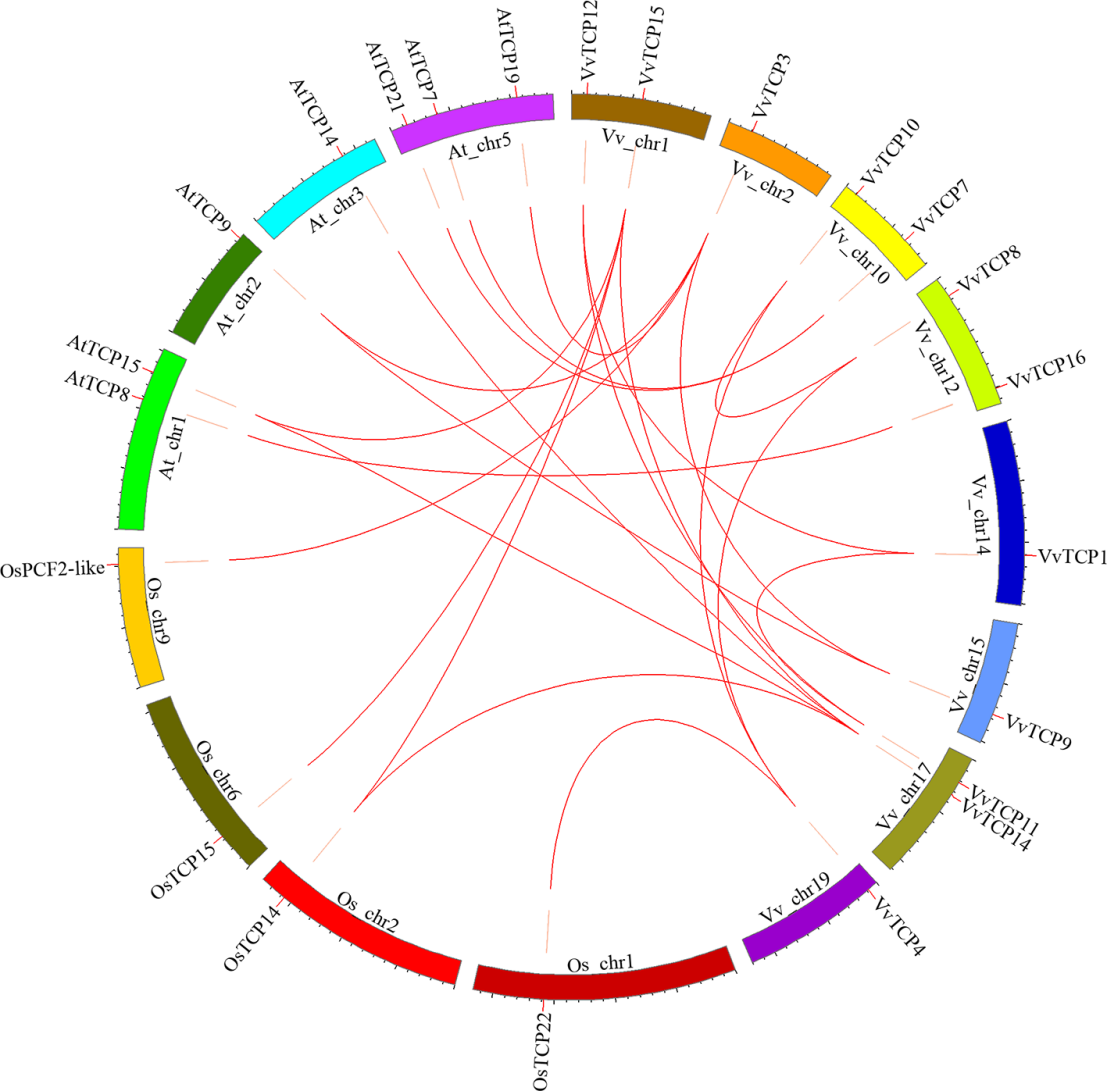
Syntenic block among TCPs from *Vitis vinifera*, *Arabidopsis thaliana*, and *Oryza sativa*. Chromosomes of *V. vinifera*, *A. thaliana*, and *O. sativa* are shown in different colors and in circular form. The approximate positions of the *VvTCPs*, *OsTCPs*, and *AtTCPs* are labeled with a short red line on the circle. Colored curves represented the syntenic relationships between grapevine, *O. sativa*, and *A. thaliana* TCP genes.

To further explore the origin and probable evolutionary mechanisms of the *VvTCPs* family, we also investigated the syntenic blocks between *Vitis* and other plant species, and detected that syntenic relationship was observed between grapevine and *A. thaliana*, meanwhile between grapevine and rice (*O. sativa*) based on the results obtained from PGDD online database. The syntenic analysis demonstrated that eight syntenic gene pairs within grapevine genome included: *VvTCP1*-*VvTCP12*/*VvTCP11*, *VvTCP3*-*VvTCP9*, *VvTCP4*-*VvTCP8*/*VvTCP10*, *VvTCP8*-*VvTCP10*, *VvTCP11*-*VvTCP12*, *VvTCP14*-*VvTCP15*. The syntenic analysis showed that nine syntenic gene pairs between grapevine and *A. thaliana* included: *VvTCP3*-*AtTCP9* (*AT2G45680*)/*AtTCP19* (*AT5G51910*), *VvTCP7*-*AtTCP21* (*AT5G08330*)/*AtTCP7* (*AT5G23280*), *VvTCP9*-*AtTCP9* (*AT2G45680*), *VvTCP14*-*AtTCP15* (*AT1G69690*)/*AtTCP14* (*AT3G47620*), *VvTCP15*-*AtTCP15* (*AT1G69690*), *VvTCP16*-*AtTCP8* (*AT1G58100*). Moreover, the syntenic analysis showed that five syntenic gene pairs between grapevine and *O. sativa* included: *VvTCP3*-*OsPCF2*-like (LOC_Os09g34950), *VvTCP4*-*OsTCP22* (LOC_Os01g55100), *VvTCP14*-*OsTCP14* (LOC_Os02g51280), *VvTCP15*-*OsTCP15* (LOC_Os06g12230)/*OsTCP14* (LOC_Os02g51280) (**Figure 6**). The syntenic analyses among grapevine, *A. thaliana*, and rice showed that these genes located in corresponding syntenic blocks arose before the divergence of grapevine, *A. thaliana*, and rice.

### MiR159/miR319 Target Site Prediction

**Figure 7:**
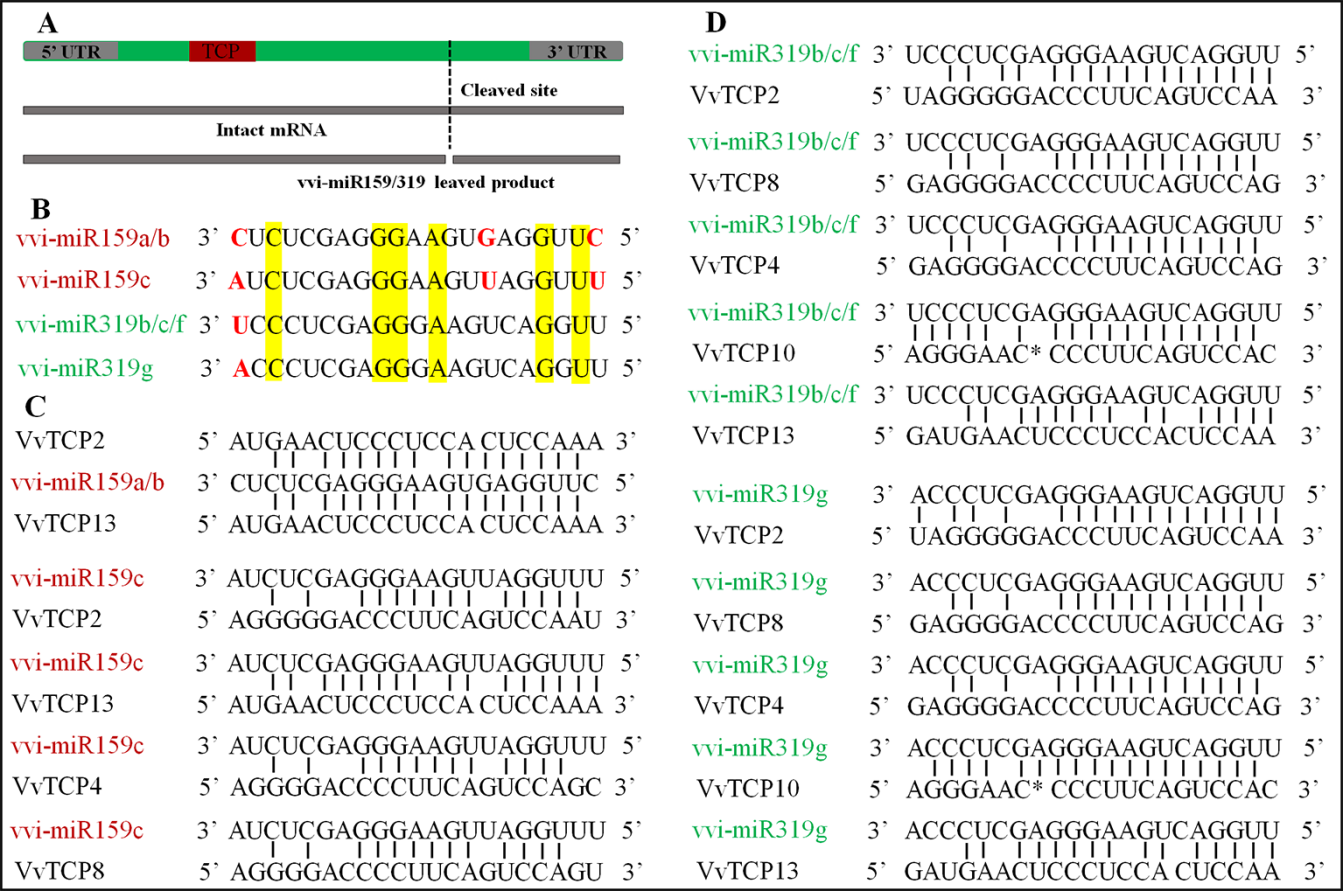
The miR319/159 binding sites of TCP gene in *Vitis vinifera*. **(A)** The TCP gene structure displayed the coding region (green), the TCP domain (red). **(B)** The sequence alignment of vvi-miR319 and vvi-miR159 in grapevine. **(C)** Alignment of vvi-miR159 complementary sequences with *V. vinifera* TCP genes. **(D)** Alignment of vvi-miR319 complementary sequences with *V. vinifera* TCP genes.

In *V. vinifera*, three vvi-miR159 family members (vvi-miR159a, b, and c) and four miR319 family members (vvi-miR319b, c, f, and g) stored in miRBase (http://www.mirbase.org/summary.shtml?org=vvi). A result of two putative miR159-target genes (*VvTCPs 2* and *13*) was attained from the analysis of psRNATarget software with the full-length nucleotide sequences of *VvTCPs* and the mature sequence of vvi-miR159a, and b. However, four putative miR159-targeted TCP genes (*VvTCPs 2*, *4*, *8*, and *13*) were predicted based on the mature vvi-miR159c sequence in the psRNATarget online application. And all miR159-targeted TCP genes belonged to CIN subclass. Meanwhile, five putative miR319-targeted TCP genes (*VvTCPs 2*, *4*, *8*, *10*, and *13*) were predicted with the full-length nucleotide sequences of *VvTCPs* and the mature sequence of vvi-miR319b, c, g and f. And all target genes for four vvi-miR319, which belonged to CIN subclass. Likewise, five TCP genes (*AtTCPs 2*, *3*, *4*, *10*, and *24*) were target by miR319 in *Arabidopsis*, and all of them were also CIN subclass. The phenomenon showed that the miR319-target sites were reserved during the diversification and evolution of the plants. Previous reports have shown that the mature sequence of miR159 and miR319 were very similar, however, they were encoded by different precursors (Schommer et al., 2012). Sometimes, they were assigned in the same family. In *V. vinifera*, the vvi-miR159 sequence was discordant with miR319 aside from six same base (**Figures 7A, B**). Thus, we considered vvi-miR159 and vvi-miR319 is not belong same miRNA family in *V. vinifera*. The alignments of the reverse vvi-miR159/319 sequence and VvTCP genes showed that these VvTCPs are complementary to the vvi-miR159/319 sequence (**Figures 7C, D**). Though, the mismatch of vvi-miR159/319 and their targets raised to 3–8 bases, the regulation of TCP TFs through vvi-miR159/319 was considered to be possible in grapevine.

### Tissue-Specific Expression Patterns of VvTCP Genes in Grapevine

**Figure 8:**
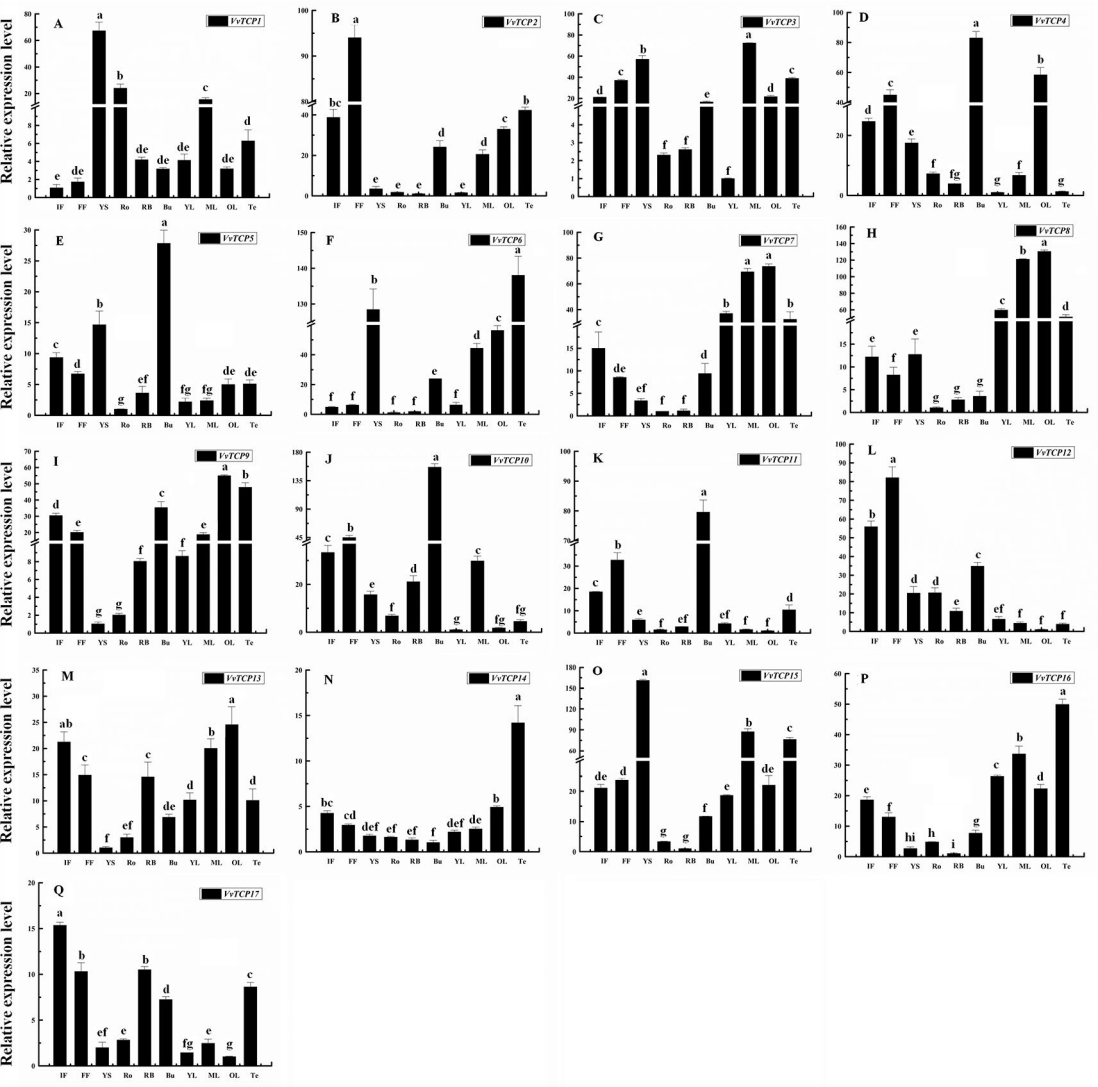
Relative expression levels of *VvTCPs* in different organs of ‘Jumeigui' grapevine. Values were normalized against the expression data of *KyActin1* and are given as means ± standard error among three biological replicates. Different letters indicate significant differences between genes (*p* < 0.05). The expression levels were calculated based on the 2^−ΔΔCt^ method.

To explore the tissue-specific expression patterns of *TCP* genes in *V. vinifera*, we analyzed the transcripts of *VvTCPs* utilizing qRT-PCR analysis of different tissues, including fruits, leaves, roots, buds, stems, tendrils and flowers from the ‘Jumeigui' grapevine. As shown in **Figure 8**, 17 *VvTCPs* exhibited tissue-specific expression patterns, potentially showing the functional divergence of *VvTCPs* in all tested tissues. For example, *VvTCP11* and *VvTCP12*, the members of CYC/TB1 subclass, are both highly expressed in flower and bud, whereas poorly expressed in YS, Ro, RB, leaves, and Te. As shown in **Figure 3**, *VvTCP11* and *VvTCP12* possessed the closest relationship in the phylogenetic tree of *VvTCPs*, further implying their functional similarity on regulating the growth and development of various tissues. The expression level of *VvTCP1* belonging to CYC/TB1 subclass, was higher in the YS, Ro, ML than other selected tissues. For the CIN subclass, the transcript accumulation levels of *VvTCP8* and *VvTCP13*, were higher in ML and OL, however low expression levels were observed in Ro, displaying that they might play a vital role in the growth and development of ML and OL. *VvTCP4* and *VvTCP10* presented very high expression levels in Bu and FF, however low expression levels in Ro, YS, RB, YL, and Te. In particular, *VvTCP2* were preferentially expressed at high levels in FF, which indicated that it might make a difference in flower growth and development. Additionally, PCF subclass displayed more extensive tissue-specific expression patterns; such as, *VvTCP6*, *VvTCP14*, and *VvTCP16* were hugely expressed in Te; *VvTCP7* and *VvTCP9* displayed high relative transcript levels in Te, ML and OL; and *VvTCP3* and *VvTCP15* are highly expressed in YS, Te, and ML, *VvTCP17* exhibited higher expression levels in flowers. Remarkably, qRT-PCR analysis showed that *VvTCP5* preferentially expressed at high levels in flowers, which indicated that they might play a vital role in flower growth and development. The results urge us to explore the expression level of *VvTCPs* during different berry developmental stages.

### Expression Patterns of VvTCPs During Different Fruit Developmental Stages

**Figure 9:**
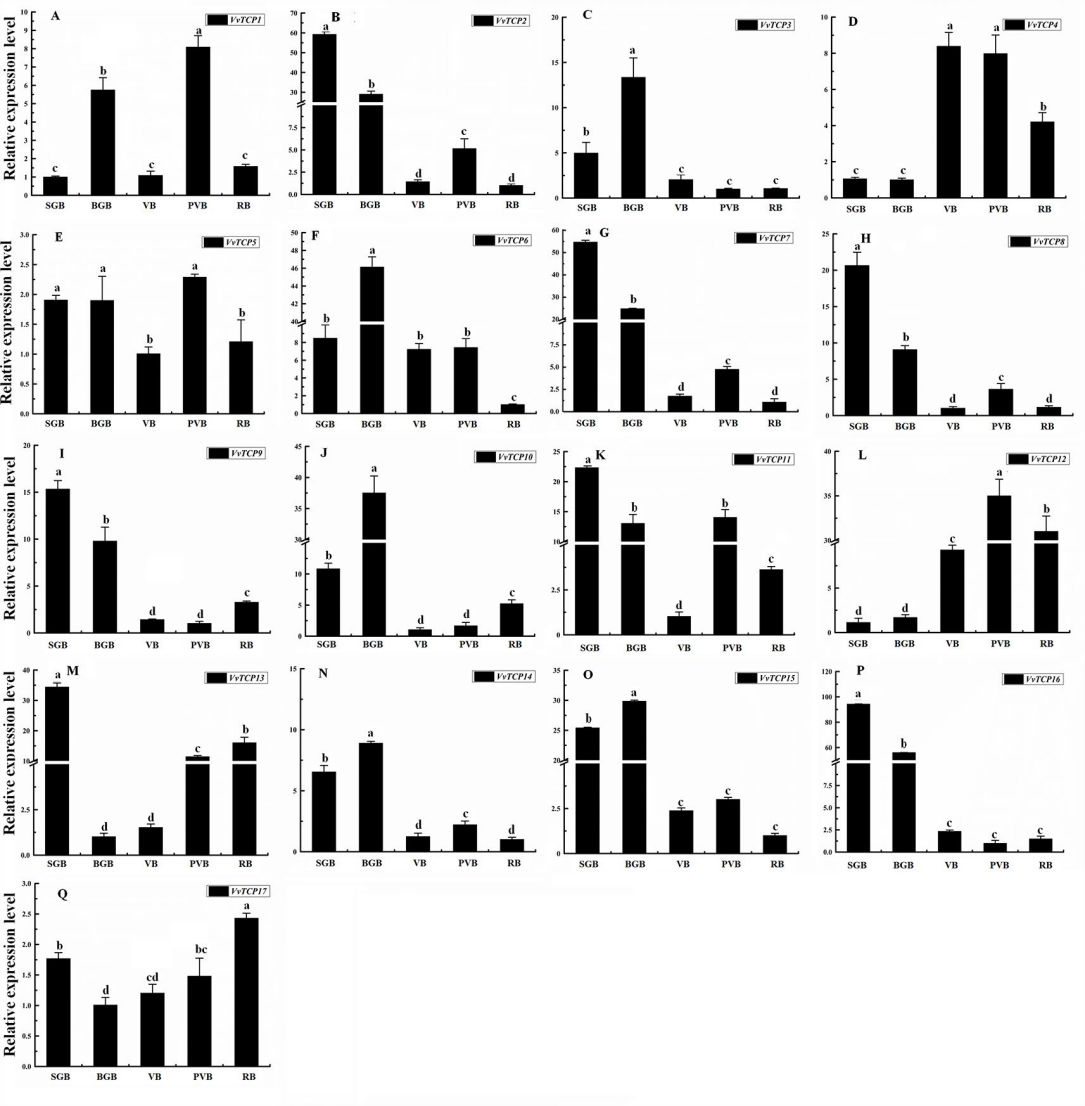
Relative expression levels of *VvTCPs* in different fruit development of ‘Jumeigui' grapevine. Values were normalized against the expression data of *KyActin1* and are given as means ± standard error among three biological replicates. Different letters indicate significant differences between genes (*p* < 0.05). The expression levels were calculated based on the 2^−ΔΔCt^ method.

**Figure 10:**
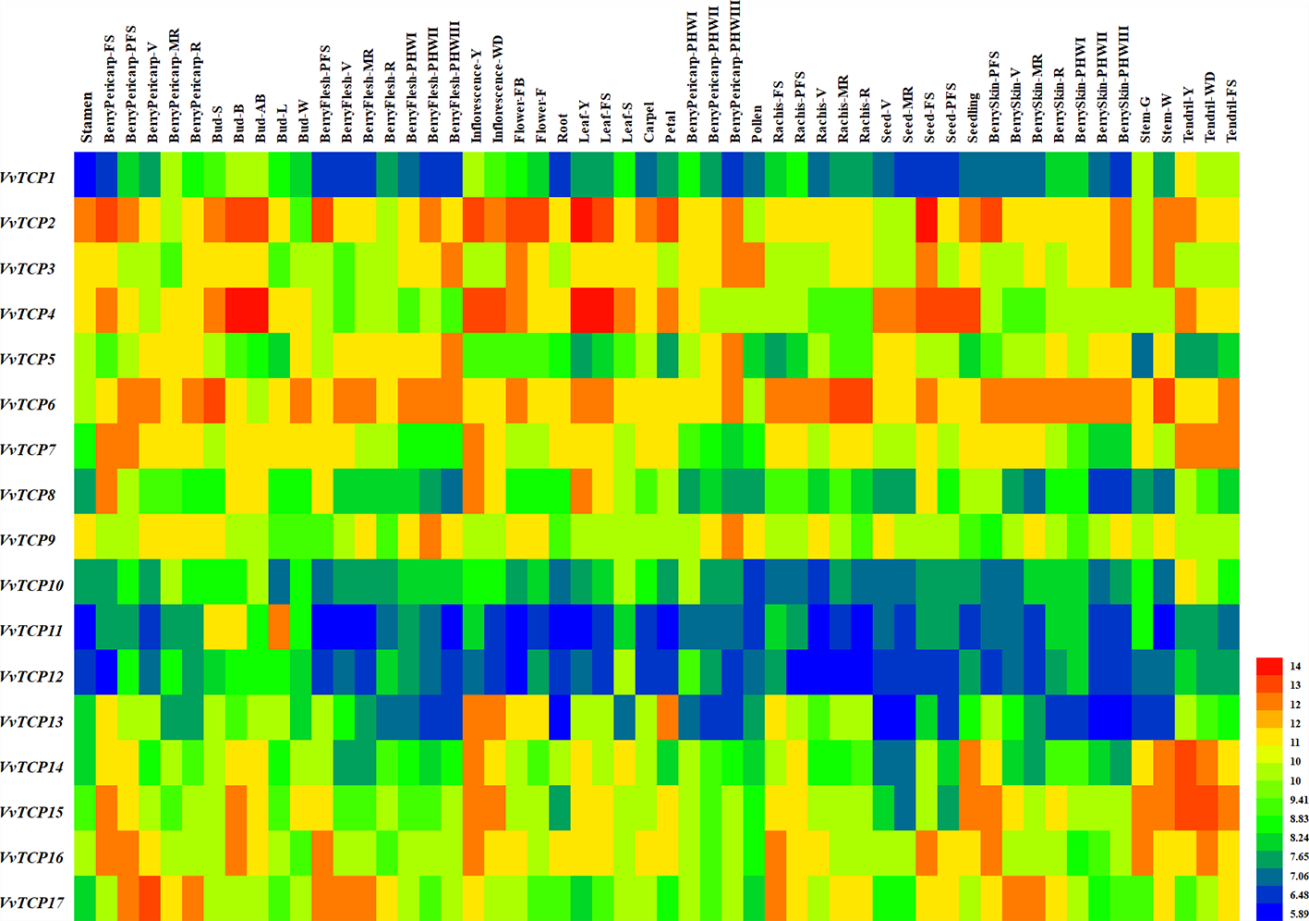
Expression profiles of *VvTCP* genes in the *V. vinifera* cv. ‘Corvina' atlas (GEO accession: GSE36128). Data were normalized based on the mean expression value of each gene in all tissues analyzed. The mean expression values were again normalized using logarithm with the base of 2 using the Heml software. Blue and red boxes show low and high expression levels, respectively, for every gene.

To illuminate their functions in fruit development, qRT-PCR were employed to explore the expression levels of 17 *VvTCP* genes at different fruit developmental stages. As indicated in **Figures 9** and **10**, the expression level of eight *VvTCPs* (*VvTCPs 2*, *6*, *7*, *9*, *10*, *14*, *15*, and *16*) were down-regulated toward fruit maturity, which suggested that they might function mainly in the early period of berry development. As shown in **Supplementary Table S2**, *VvTCPs 3*, *8*, and *11* harbored GCN4_motif *cis*-elements involving in endosperm expression, which is consistent with high expression level of three *VvTCPs* in early period of fruit development. On the contrary, the transcripts of three genes (*VvTCPs 4*, *12*, and *17*) were up-regulated during the ripening process, which suggested that they might play a vital important role in fruit maturation. Same expression levels were observed for *VvTCP5*, which indicated that *VvTCP5* participate in the whole process of berry development. *VvTCP1* showed a jump expression patterns during different fruit developmental stages. *VvTCP1* showed high expression levels at BGB and PVB in comparison with the other stages. The expression patterns of *VvTCP13* during SGB period was significantly high as compared to the other stages. The relative expression level of *VvTCP13* showed a declined trend from SGB to BGB and then exhibited a continuous increasing trend as fruit proceeds toward maturity, indicating that *VvTCP13* played different roles in different stages of fruit development.

### Transcript Analysis of VvTCP Genes in Response to ABA Treatment

**Figure 11:**
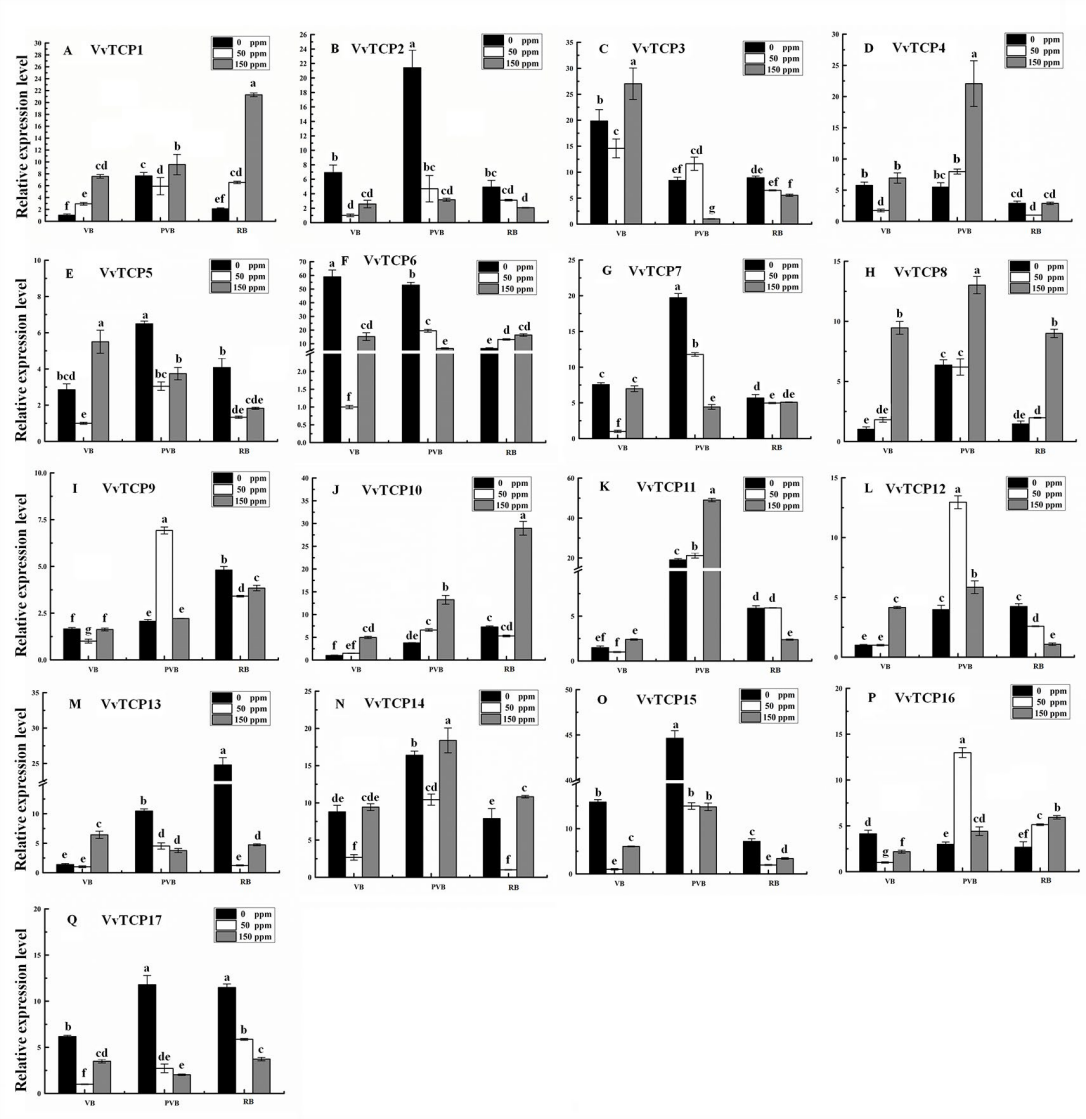
Relative expression levels of *VvTCPs* under abscisic acid treatment. Values were normalized against the expression data of *KyActin1* and are given as means ± standard error among three biological replicates. The expression levels were calculated based on the 2^−ΔΔCt^ method.

Abscisic acid (ABA) have engrained roles in fruit developmental processes as well as stress signaling networks (Bari and Jones, 2009). As shown in **Supplementary Table S2**, ABA-responsive *cis*-element, ABRE, were observed in several *VvTCPs*. In order to comprehend how *VvTCPs* express in response to ABA treatment, qRT-PCR was employed to analyze *VvTCP* transcripts respond to ABA, which has three concentration levels (0, 50, 150 ppm). The expression levels of four *VvTCPs* (*VvTCPs 1*, *10*, *14*, and *16*) were all enhanced with 50 ppm, 150 ppm ABA compared with the 0 ppm ABA at VB, PVB, and RB period, which is consistent with presence of ABA-responsive *cis*-element (ABRE) in the promoter of these *VvTCPs*, further implying ABA may play an important role in berry ripening (**Figure 11**). On the contrary, the expression levels of five *VvTCPs* (*VvTCPs 2*, *6*, *7*, *15*, and *17*) were all suppressed with 50 ppm, 150 ppm ABA compared with the 0 ppm ABA at VB, PVB, and RB period. *VvTCP11* transcript was enhanced with 150 ppm ABA compared with the 0 ppm ABA at VB and PVB period, whereas it was suppressed with 150 ppm ABA compared with the 0 ppm ABA at RB period. The expression levels of two *VvTCPs* (*VvTCPs 5* and *13*) were all enhanced with 150 ppm ABA compared with the 0 ppm ABA at veraison, however they were suppressed with 50 and 150 ppm ABA compared with the 0 ppm ABA at PVB and RB period. The expression levels of two *VvTCPs* (*VvTCPs 9* and *12*) were strongly enhanced with 50 ppm ABA at PVB period compared with the 0 and 150 ppm ABA at veraison and RB period.

### Subcellular Localizations

**Figure 12:**
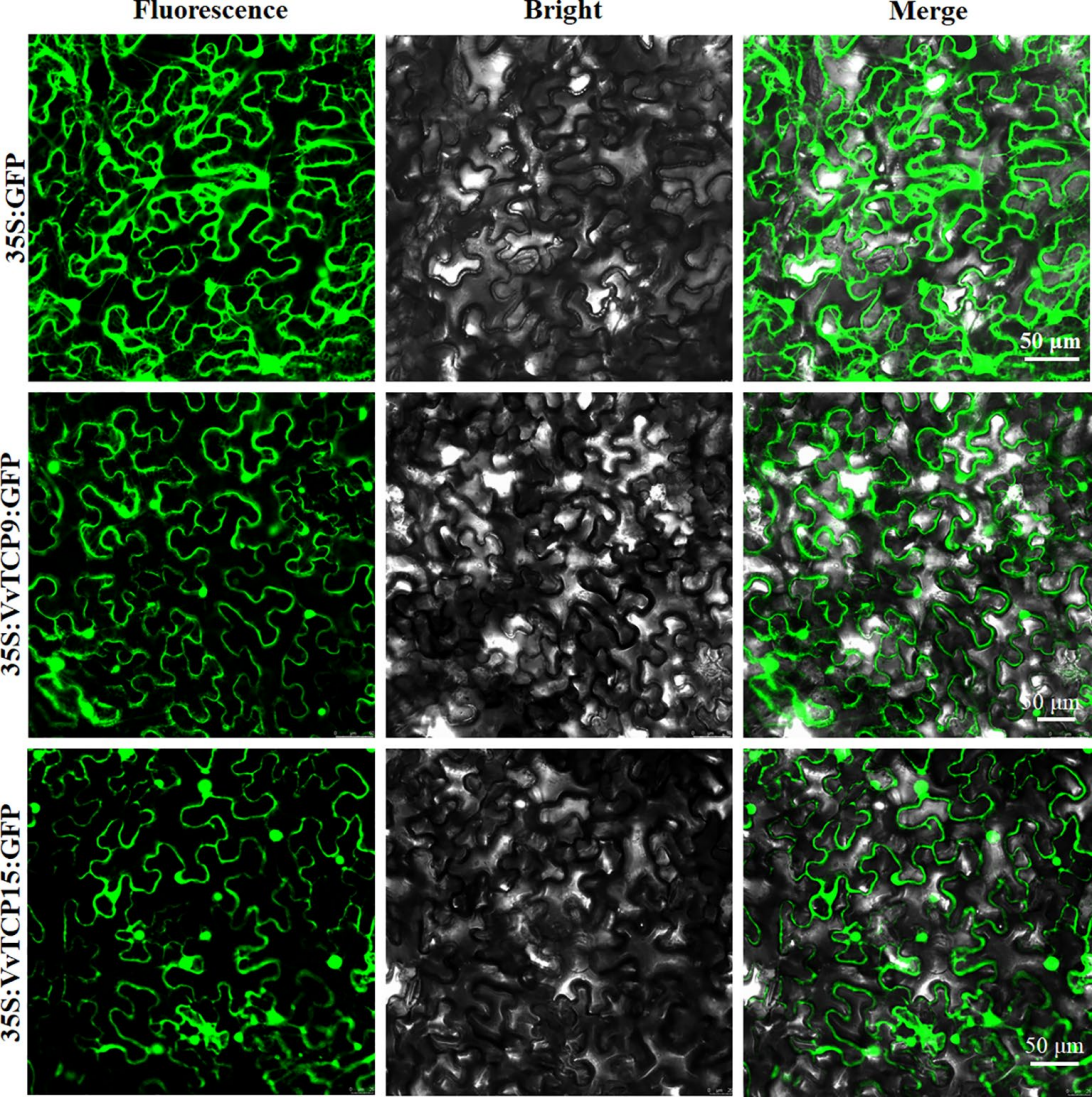
The leaves of 3 to 5-week-old *Nicotiana benthamiana* plants were transiently transformed with control, 35S-*VvTCP9*-GFP, and 35S-*VvTCP15*-GFP. Images under blight field (middle), fluorescence (left), and the merged images are shown on the right. Bar: 50 μm.

To investigate the function of VvTCPs, their subcellular localizations were measured by the fluorescent protein-tagging method. Firstly, the full-length open reading frames (ORFs) lacking the stop codon of two VvTCPs were merged to the N-terminal of green fluorescence protein (GFP) protein driven by CaMV 35S promoter, generating fusion proteins p35S-*VvTCP9*-GFP and p35S-*VvTCP15*-GFP were agroinfiltrated into leaves of 3 to 5-week-old *N. benthamiana* plants. Fluorescence microscopy exhibited that the PHB-GFP was homogenously disseminated throughout the whole cell, the fusion proteins of p35S-*VvTCP9*-GFP, and p35S-*VvTCP15*-GFP were observed in the plasma membrane and nucleus (**Figure 12**). The results demonstrated that VvTCP9 and VvTCP15 were both nuclear- and cytomembrane-localized protein. Different from other nuclear-localized TFs, VvTCP9, and VvTCP15 also localized in the cytomembrane, which indicated that two VvTCPs (VvTCP9 and VvTCP15), as well as other single nuclear-localized TCP TFs may possessed diversity function.

## Discussion

TCP proteins are a type of plant-specific TFs, which participate in numerous biological activities throughout the growth and development in plants (Manassero et al., 2013). To date, many TCP genes have been reported in some studies with genome-wide characterization, such as, *G. raimondii* (Ma et al., 2014), *Malus domestica* (Xu et al., 2014), *L. esculentum* (Parapunova et al., 2014), *Arabidopsis* (Li, 2015), *C. lanatus* (Shi et al., 2016), and *B. rapa* (Du et al., 2017). Nevertheless, no comprehensive analyses of TCP TFs in *V. vinifera* have been done. In the present study, we performed a comprehensive analysis of the *VvTCPs* family in grapevine by exploring their evolutionary relations, exon-intron organization, conserved motifs, *cis*-regulatory elements, gene duplication and syntenic analysis, and expression patterns in various tissues and developmental phases and under ABA treatment situations, and sub-cellular localizations.

### TCP Genes Family in *Vitis vinifera* and Their Evolution

In the present study, a sum of 17 *VvTCP* genes were identified from the grapevine genome, and the TCPs number of *V. vinifera* was less than those of *Arabidopsis*, *O. sativa*, *L. esculentum*, *P. mume*, *C. lanatus*, turnip, Chinese cabbage, and *G. raimondii*, which had 24, 22, 30, 19, 27, 39, 39, and 38 TCP members, respectively (**Table 2**). The quantity of TCP genes in turnip and *G. raimondii* are almost 2.29 and 2.24 times more than that in *V. vinifera*, which are inconsistent regarding the coding genes in turnip genome (41,174 genes, http://brassicadb.org/brad/speciesinfo.php) and *G. raimondii* genome (40,976 genes) are about 1.56 and 1.55 times more than that in grapevine (26,346 genes) (Wang et al., 2012). It is observed that many *BrTCP* genes contain two or more counterparts in turnip and *G. raimondii*, suggesting that the enlargement of TCP gene family in turnip and *G. raimondii* might be due to genome duplication events. Therefore, we consider that the difference of genome duplication events may be the main reason for this inconsistency. The phylogenetic tree among *V. vinifera*, *Arabidopsis*, *O. sativa*, *C. lanatus*, and *B. rapa* supported the previous described division of TCP genes family into two subfamilies: class I (PCF) and class II (CYC/TB1 and CIN) (Navaud et al., 2007; Martin-Trillo and Cubas, 2010). TCP gene family in *V. vinifera* also consisted of these three types: PCF with nine members, CIN with five members, and CYC/TB1 with three members (**Table 2**). Nearly all the VvTCPs had the conserved motif 1 and motif 2 in *V. vinifera*. Besides conservative motif 1 and motif 2, the VvTCPs of PCF type usually has motif 4 in front of motif 1 in proteins. Motif 5 was only identified in the VvTCPs of class II. Moreover, motif 12 was only found in VvTCP1, VvTCP11, and VvTCP12 of the CYC/TB1 type, which contained an R domain (**Figure 1B**). Furthermore, motifs 1–2 were specific to the TCP domain, which was formed by a bHLH-motif of 54–59 AA remains. These showed that *VvTCPs* could play vital roles in plant growth and development, even though they belong to identical TCP subclass. Moreover, most of VvTCP proteins are belong to the same subclass that not only shared similar conserved domain but also similar gene structure.

**Table 2:** The number of TCP family genes in *Vitis vinifera*, *Arabidopsis thaliana*, *Oryza sativa*, *Lycopersicon esculentum*, *Prunus mume*, Citrullus lanatus, turnip, Chinese cabbage, and *Gossypium raimondii*.

Species	PCF	CIN	CYC/TB1	Total
*Vitis vinifera*	9	5	3	17
*Arabidopsis thaliana*	13	8	3	24
*Oryza sativa*	10	9	3	22
*Lycopersicon esculentum*	13	11	6	30
*Prunus mume*	10	3	6	19
*Citrullus lanatus*	12	9	6	27
Turnip (*Brassica rapa* ssp. *rapa*)	20	13	6	39
Chinese cabbage (*Brassica rapa* L. ssp. *pekinensis*)	19	14	6	39
*Gossypium raimondii*	25	9	4	38

### Role of *Cis*-Elements in the Transcriptional Regulation of VvTCP Genes

As the key unit of transcriptional regulation, *cis*-elements participated in the regulation of molecular networks in many biological processes (Ibraheem et al., 2010). In terms of *cis*-regulatory element sequences, the promoters of *VvTCPs* have vastly repetitive regions and some common motifs. The minimum 25 *cis*-regulatory elements (ACE, GT1-motif, MRE, G-Box, etc.) in this study are necessary for light-driven transcriptional regulation in the promoter of *VvTCPs*, which are in accord with the important role of *VvTCPs* in the light-responsive process. Moreover, the existence of the circadian element shows the circadian regulation is very important for the *VvTCP* genes expression. Some *VvTCPs* (e.g., *VvTCP1*, *VvTCP*8, *VvTCP10*, *VvTCP14*, and *VvTCP16*) existed in ABA-responsive element (ABRE), which were in accord with the increased expression level of these VvTCPs under the ABA treatment. The results of *cis*-elements analyses showed the presence of unique *cis*-regulatory elements in the promoter of only one TCP gene, hence deducing the specificity of these genes expression. The unique *cis*-regulatory elements were recognized due to the length of their sequences (6–15 bases) but not usually easy to generate some nucleotide variabilities. Interestingly, out of the 23 unique *cis*-elements, four were identified only in the *VvTCP1* promoter, followed by three in *VvTCP2*, two in *VvTCP3*, *VvTCP11*, *VvTCP14*, and *VvTCP16*, and only one in *VvTCP5*, *VvTCP9*, *VvTCP10*, *VvTCP13*, and *VvTCP16*. As we all known, gene duplication is the initial driving force for new functions in the development of genomic and genetic systems, and is one of the main evolutionary mechanisms further leading to species formation and differentiation (Lynch and Conery, 2000). Previous reports indicated that the enlargement of gene families is largely caused by gene duplication events on several types, including WGD, tandem duplication, segmental duplication, and other transposition events (Moore and Purugganan, 2003). Our results demonstrate that singleton duplication is a predominant type for *VvTCP* genes and the key factor to the enlargement of *VvTCPs* family in grapevine.

### Differential Expression Profiles of MiR319/miR159-Targeted VvTCP Genes in *Vitis vinifera*

Previous reports indicated that more than half of the target genes of miRNA are TFs, such as MYB, TCP, AP2, etc. (Aukerman and Sakai, 2003; Martin-Trillo and Cubas, 2010; Xu et al., 2015). Concerning *V. vinifera* TCP genes, four of them (*VvTCPs 2*, *4*, *8*, and *13*) were predicted to be targeted by vvi-miR159. Additionally, five *VvTCP* genes (*VvTCPs 2*, *4*, *8*, *10*, and *13*) were predicted to be targeted by vvi-miR319. In *V. vinifera*, the sequence of vvi-miR319 was obviously different with respect to vvi-miR159, but they are predicted to enable to regulate the same *VvTCP* genes. However, miR159 and miR319 possessed similar mature sequences, whereas they targeted different genes in *Arabidopsis*. Though miR159 did not target the *TCP* genes, it had same role with miR319 in regulating flower development in plants (Hong and Jackson, 2015). Current studies showed that five *VvTCP* genes (*VvTCPs 2*, *4*, *8*, *10*, and *13*) were targeted by vvi-miR319. The mRNA fragments of TCP gene are produced by a miR319a-mediated cleavage and this have been verified by *in vivo* experiment (Palatnik et al., 2003). In plants, the miR319 family involved in regulating floral development with the miR319-mediated-TCP model. The flaws in stamen and petal development of *Arabidopsis* were found in the mutants of miR319 loss-of-function (Nag et al., 2009). Similar to miR159, over-expression of miR319 leads to male sterile and stamen imperfections (Palatnik et al., 2007). In this study, four target genes (*VvTCPs 2*, *4*, *10*, and *13*) were all expressed strongly in flower, especially at FF time. Thus, *VvTCPs* could possess an interactional and coefficient relations in the floral grow and development process of *V. vinifera*.

### Potential Roles of VvTCP Genes in *Vitis vinifera*

Many evidences have already suggested that TCP genes participate in regulating cell propagation and growth, and playing multiple roles in the process of growth and development in plants (Martin-Trillo and Cubas, 2010). The genes of CYC/TB1 subclass are mostly participated in the growth and development of axillary meristems that are responsible for the growth of either lateral shoots or flowers. *AtTCP1*, belonging to CYC/TB1 subclass, is participated in the longitudinal elongation of some tissues, such as inflorescent stems, rosette leaves, and petioles. *AtTCP1* is expressed strongly in the distal region of growing rosette leaves, the lower part of the inflorescence stem, and the midrib region of the petiole and blade (Koyama et al., 2010). In addition, the mutant plants of gain-of-*tcp1-1D* have lengthened petioles and leaves, while the plants of *TCP1-SRDX* show reduced statures and petioles, as well as epinastic and rounded leaves (Guo et al., 2010). *VvTCP1*, which is closely related to *AtTCP1*, was highly expressed in YS, ML, and root (**Figure 8**). *AtTCP18* (*BRC1*, *BRANCHED1*), a homolog of *TB1* in *Arabidopsis*, was highly expressed in some tissues that mainly included axillary buds, such as stem, silique, inflorescence, and leaf base (Aguilar-Martinez et al., 2007). *AtTCP18* acts downstream of strigolactone and auxin to regulate the outgrowth of axillary bud, and the mutant plants with decreased activity of either gene display an increasing amount of the rosette branches. Conversely, the inhibition effect in lateral branches were observed in plants due to the up-regulated expression of *AtTCP18*. *VvTCP11*, which is closely related to *AtTCP18*, exhibits a significantly higher relative expression level in the bud (**Figure 8**). To some extent, this result is in accord with the expression level of *AtTCP18* and indicates that *VvTCP11* likely play a significant role in axillary bud development, similar to *AtTCP18*, in grapevine. Additionally, the TCPs of CYC/TB1-type might be less ancient than CIN subclass, and CIN-type TCPs play a vital role in the leaf growth and development. For instance, eight CIN-type genes (*AtTCPs 2*, *3*, *4*, *5*, *10*, *13*, *17*, and *24*) displayed relatively high expression levels in leaves and were capable for the regulation of leaf growth and development in *Arabidopsis* (Palatnik et al., 2003; Ori et al., 2007). Likewise, five CIN-subclass genes in *V. vinifera* except *VvTCP10* were highly expressed in ML or OL. The above results showed that the regulation of leaf growth and development by *TCP* genes that are homologous to those in grapevine might be similar in *Arabidopsis*.

In *VvTCPs* of PCF-type, all genes displayed widespread expression level, implying these genes might play important roles in numerous different development phases. In *Arabidopsis*, *AtTCP14* involved in regulating embryonic growth development through seed sprouting, which was closely linked to ABA responses (Tatematsu et al., 2008). In the present study, *VvTCP14*, and *VvTCP17* were phylogenetically close to *AtTCP14*. Among them, *VvTCP14* exhibited higher transcript expression levels after 150 ppm ABA treatment, however, *VvTCP17* was down-regulated expression after ABA treatment at different berry development stages, implying they might play distinct roles in berry development in response to ABA. In grapevine, the endosperm plays a vital role in fruit setting as well as early stage of berry development. Promoter analysis showed that four (*VvTCPs 3*, *5*, *8*, and *11*) of 17 *VvTCP* genes harbored GCN4_motif *cis*-elements participated in endosperm expression (**Figure 5A** and **Supplementary Table S2**). Hence, it concluded that these *VvTCP* genes are responsible for fruit development and ripening in grapevine. In tomato, some *SlTCPs*, for instance, *SlTCP18*, *SlTCP15*, and *SlTCP12*, are preferentially expressed in fruits. Furthermore, these genes are controlled by SlAP2a (APETALA2a), SlCNR (COLORLESS NON-RIPENING), and SlRIN (RIPENING INHIBITOR) proteins, which are important TFs with vital roles in ripening, implying an important role in fruit development or ripening of tomato (Parapunova et al., 2014). In grapevine, *VvTCP6* and *VvTCP7*, which are homologs of *SlTCP18* and *SlTCP15*, respectively, also exhibited high expression levels before the version of berry, demonstrating that *VvTCP6* and *VvTCP7* might play important roles in early berry development of grapevine (**Figures 9** and **10**).

## Data Availability Statement

Publicly available datasets were analyzed in this study. This data can be found here: http://genomes.cribi.unipd.it/grape/index.php; http://www.arabidopsis.org; http://planttfdb.cbi.pku.edu.cn/ , v3.0.

## Author Contributions

SJ designed and performed most of the experiments and drafted the manuscript. YX and JW provided technical assistance. SW, CZ, MA, and LG revised the manuscript. LW and CM participated in the sequence alignment. MZ, WX, and WM performed the statistical analysis. CZ agrees to serve as the author responsible for contact and ensures communication. All authors reviewed and approved the final submission.

## Funding

The study was supported by grants from National Postdoctoral Program for Innovative Talents (Grant No. BX20180199), China Postdoctoral Science Foundation (Grant No. 2018M642028), the Special Funds of Modern Industrial Technology System for Agriculture (Grant No. CARS-29-zp-7), National Key Research and Development Plan project (Grant No. 2018YFD0201305).

## Conflict of Interest

WM was employed by the company Agriculture, Forestry and Animal Husbandry Technology Promotion Service Center of Ningxia Agricultural Group.

The remaining authors declare that the research was conducted in the absence of any commercial or financial relationships that could be construed as a potential conflict of interest.

The reviewer YXP and handling Editor declared their shared affiliation.
