# Supplementary material for: Genome-Wide Identification, Characterization, and Transcript Analysis of the TCP Transcription Factors in Vitis vinifera
Source: Front Genet. 2019 Dec 20;10:1276. doi: 10.3389/fgene.2019.01276 (PMC6934063; doi:10.3389/fgene.2019.01276)
Supplement: Supplementary file 1 [file DataSheet_1.docx]

**Table S1.** The primers used for the *VvTCP*s in the present study.

| **Primer Names** | **Sequence (5’-3’)^1^** | **Description^2^** |
| --- | --- | --- |
| VvTCP1-qF1 | TGTGCACCAGAAGGCTTGAT | qRT-PCR |
| VvTCP1-qR1 | GCCATGTTGTGGGTATGGGA | qRT-PCR |
| VvTCP2-qF1 | GGCTGTGCTAGCAATAACGC | qRT-PCR |
| VvTCP2-qR1 | CACCGGGATAAGATGGTCGG | qRT-PCR |
| VvTCP3-qF1 | CCCTCAGTAAGCCCCGTTTT | qRT-PCR |
| VvTCP3-qR1 | CTGAGATCGTTGCGCTGTTG | qRT-PCR |
| VvTCP4-qF1 | GTGCAAATGCATCACGGCAA | qRT-PCR |
| VvTCP4-qR1 | CTGTACGGACGAAGACGAGG | qRT-PCR |
| VvTCP5-qF1 | AACCGCTTACGTCAGTTCCC | qRT-PCR |
| VvTCP5-qR1 | CAACAACGATGTGAACGGCA | qRT-PCR |
| VvTCP6-qF1 | CCAGTTGGGCAATGGTAGGT | qRT-PCR |
| VvTCP6-qR1 | AAGCTGCTGGTTACTCCCAC | qRT-PCR |
| VvTCP7-qF1 | GAGACCTGATTTCGGGCAGG | qRT-PCR |
| VvTCP7-qR1 | CAACGACGCCAGCAAATTCA | qRT-PCR |
| VvTCP8-qF1 | ACTACCCGCCTGATAGCAAC | qRT-PCR |
| VvTCP8-qR1 | CAAATCCACCGCGACTCTCT | qRT-PCR |
| VvTCP9-qF1 | AGACGATACGAGAGCCGTTG | qRT-PCR |
| VvTCP9-qR1 | AGGCTCTGCCTTCAACGAAA | qRT-PCR |
| VvTCP10-qF1 | AGCTTCCAAGACCCAACACC | qRT-PCR |
| FvTCP10-qR1 | GGGTTCCCTCTCGGAAAACA | qRT-PCR |
| VvTCP11-qF1 | AGAGGCGGGAGCATCTCTAA | qRT-PCR |
| VvTCP11-qR1 | GCCCTTGCTCTTGCCTTTTC | qRT-PCR |
| VvTCP12-qF1 | AAGTCCCGCCGATGAAGAAG | qRT-PCR |
| VvTCP12-qR1 | TGCTGCTTTGGACTTGGTGA | qRT-PCR |
| VvTCP13-qF1 | TTTTGGTTTGTCCACCCGGA | qRT-PCR |
| VvTCP13-qR1 | TGCAAGGGTCTCACAGGTTG | qRT-PCR |
| VvTCP14-qF1 | AAGGATCGGCACACCAAAGT | qRT-PCR |
| VvTCP14-qR1 | TTGAGATGGGACGGACATGC | qRT-PCR |
| VvTCP15-qF1 | TCCAATGGCTCTCTTGCCTG | qRT-PCR |
| VvTCP15-qR1 | CTCCGTTGTGGGGATGTGAA | qRT-PCR |
| VvTCP16-qF1 | CATCGCTACCAGATCCGAGG | qRT-PCR |
| VvTCP16-qR1 | ACTGAAAAACCCTAGCCGCA | qRT-PCR |
| VvTCP17-qF1 | GACCGCCATGGTCCATACAT | qRT-PCR |
| VvTCP17-qR1 | CGGCACAGATAGCTGGCATA | qRT-PCR |
| Actin-qF | GATTCTGGTGATGGTGTGAGT | qRT-PCR |
| Actin -qR | GACAATTTCCCGTTCAGCAGT | qRT-PCR |
| VvTCP9-F1 | ACCAGTCTCTCTCTCAAGCTT ATGACTTCAGTTCAGAAACAA | Subcellular localization |
| VvTCP9-R1 | GCTCACCATGGATCCAAGCTT ATGCTTTGAGGAGCGAGACAT | Subcellular localization |
| VvTCP15-F1 | ACCAGTCTCTCTCTCAAGCTT ATGATCATGGAGGGAGAAAAT | Subcellular localization |
| VvTCP15-R1 | GCTCACCATGGATCCAAGCTT GCTAGTTGTATCATGTCTATG | Subcellular localization |

1 Restriction sites are indicated in red.

2 The type of experiment for which the primers were used is indicated in brackets (qRT-PCR: quantitative reverse transcription PCR).

| **Table S2 The *cis*-element analysis of VvTCP promoter in grape (*Vitis vinifera* L.)** | | | | | | | | | | | | | | | | | | | | |
| --- | --- | --- | --- | --- | --- | --- | --- | --- | --- | --- | --- | --- | --- | --- | --- | --- | --- | --- | --- | --- |
| Code | Motif | Function | Sequence | VvTCP1 | VvTCP2 | VvTCP3 | VvTCP4 | VvTCP5 | VvTCP6 | VvTCP7 | VvTCP8 | VvTCP9 | VvTCP10 | VvTCP11 | VvTCP12 | VvTCP13 | VvTCP14 | VvTCP15 | VvTCP16 | VvTCP17 |
| 1 | ABRE | abscisic acid responsiveness | ACGTG | 2 | 0 | 1 | 0 | 0 | 3 | 3 | 0 | 0 | 3 | 2 | 2 | 0 | 3 | 3 | 2 | 1 |
| 2 | AuxRR-core | auxin responsiveness | GGTCCAT | 0 | 0 | 0 | 0 | 0 | 0 | 0 | 0 | 0 | 0 | 0 | 0 | 0 | 0 | 1 | 0 | 0 |
| 3 | CGTCA-motif | MeJA-responsivenes | CGTCA | 1 | 1 | 0 | 0 | 3 | 0 | 2 | 0 | 0 | 2 | 1 | 0 | 0 | 0 | 0 | 1 | 2 |
| 4 | GARE-motif | gibberellin-responsive element | TCTGTTG | 0 | 0 | 2 | 0 | 1 | 0 | 0 | 0 | 0 | 2 | 1 | 0 | 1 | 0 | 0 | 0 | 0 |
| 5 | P-box | gibberellin-responsive element | CCTTTTG | 0 | 0 | 1 | 0 | 0 | 0 | 1 | 1 | 0 | 0 | 2 | 0 | 0 | 2 | 0 | 0 | 1 |
| 6 | TCA-element | salicylic acid responsiveness | CCATCTTTTT | 0 | 0 | 1 | 1 | 0 | 0 | 1 | 2 | 2 | 1 | 2 | 1 | 2 | 3 | 0 | 0 | 1 |
| 7 | TGA-element | auxin-responsive element | TCTTAC | 0 | 0 | 1 | 0 | 0 | 0 | 0 | 1 | 0 | 0 | 0 | 3 | 0 | 0 | 1 | 0 | 0 |
| 8 | TATC-box | gibberellin responsiveness | TATCCCA | 0 | 0 | 0 | 1 | 0 | 0 | 0 | 0 | 0 | 1 | 0 | 0 | 1 | 0 | 0 | 0 | 0 |
| 9 | ERE | ethylene-responsive element | ATTTTAAA | 1 | 0 | 0 | 5 | 0 | 2 | 4 | 3 | 5 | 1 | 0 | 1 | 1 | 0 | 0 | 6 | 0 |
| 10 | ARE | anaerobic induction | AAACCA | 3 | 3 | 2 | 2 | 0 | 2 | 1 | 4 | 2 | 2 | 2 | 0 | 2 | 3 | 1 | 1 | 4 |
| 11 | TC-rich repeats | defense and stress responsiveness | GTTTTCTTAC | 1 | 1 | 1 | 0 | 0 | 0 | 0 | 1 | 0 | 0 | 0 | 0 | 0 | 0 | 0 | 0 | 1 |
| 12 | W box | wounding and pathogens | TTGACC | 1 | 0 | 0 | 1 | 3 | 0 | 0 | 1 | 1 | 2 | 0 | 0 | 0 | 0 | 0 | 1 | 2 |
| 13 | LTR | low-temperature responsiveness | CCGAAA | 0 | 2 | 1 | 0 | 1 | 0 | 0 | 0 | ·1 | 0 | 0 | 0 | 1 | 3 | 0 | 0 | 1 |
| 14 | MBS | drought-inducibility | CAACTG | 0 | 1 | 2 | 1 | 2 | 1 | 1 | 1 | 2 | 1 | 0 | 2 | 1 | 1 | 0 | 0 | 3 |
| 15 | WUN-motif | wound-responsive element | CCATTTCAA | 0 | 1 | 0 | 1 | 1 | 0 | 3 | 0 | 1 | 1 | 1 | 0 | 0 | 1 | 0 | 1 | 1 |
| 16 | STRE | stress-responsive element | AGGGG | 1 | 5 | 3 | 6 | 7 | 6 | 7 | 6 | 0 | 3 | 4 | 3 | 3 | 2 | 10 | 2 | 0 |
| 17 | DRE-core | dehydration-responsive element | GCCGAC | 0 | 1 | 0 | 0 | 0 | 0 | 0 | 0 | 0 | 0 | 0 | 0 | 0 | 1 | 0 | 0 | 0 |
| 18 | CAT-box | meristem expression | GCCACT | 2 | 0 | 1 | 2 | 0 | 3 | 0 | 0 | 0 | 2 | 1 | 1 | 0 | 3 | 0 | 0 | 0 |
| 19 | HD-Zip 1 | palisade mesophyll cells | CAAT(A/T)ATTG | 0 | 0 | 0 | 0 | 1 | 0 | 0 | 0 | 0 | 0 | 0 | 1 | 0 | 0 | 1 | 0 | 0 |
| 20 | O2-site | zein metabolism regulation | GATGATGTGG | 2 | 0 | 0 | 1 | 1 | 0 | 1 | 0 | 0 | 0 | 0 | 1 | 0 | 1 | 0 | 1 | 0 |
| 21 | RY-element | seed-specific regulation | CATGCATG | 0 | 0 | 0 | 0 | 0 | 0 | 0 | 0 | 0 | 0 | 0 | 0 | 1 | 0 | 0 | 0 | 0 |
| 22 | GCN4_motif | cis-regulatory element involved in endosperm expression | TGAGTCA | 0 | 0 | 1 | 0 | 1 | 0 | 0 | 1 | 0 | 0 | 1 | 0 | 0 | 0 | 0 | 0 | 0 |
| 23 | circadian | circadian control | CAAAGATATC | 0 | 0 | 1 | 0 | 0 | 0 | 0 | 0 | 0 | 0 | 0 | 0 | 0 | 0 | 0 | 0 | 0 |
| 24 | CCGTCC motif | cis-acting regulatory element related to meristem specific activation | CCGTCC | 0 | 0 | 0 | 0 | 0 | 0 | 0 | 1 | 0 | 0 | 0 | 1 | 1 | 0 | 0 | 0 | 0 |
| 25 | MYC | cell growth promotion | CATTTG | 6 | 7 | 0 | 5 | 7 | 3 | 1 | 3 | 6 | 2 | 2 | 5 | 5 | 7 | 10 | 1 | 2 |
| 26 | MBSI | flavonoid biosynthetic genes regulation | AAAAGTTAGTTA | 0 | 1 | 0 | 0 | 0 | 0 | 0 | 0 | 0 | 0 | 0 | 0 | 0 | 0 | 0 | 0 | 0 |
| 27 | Box 4 | part of a conserved DNA module involved in light responsiveness | ATTAAT | 3 | 0 | 0 | 3 | 3 | 0 | 6 | 3 | 3 | 0 | 2 | 2 | 1 | 6 | 3 | 7 | 1 |
| 28 | G-box | cis-acting regulatory element involved in light responsiveness | TAACACGTAG | 2 | 1 | 3 | 0 | 0 | 2 | 1 | 0 | 0 | 1 | 4 | 1 | 1 | 1 | 1 | 1 | 1 |
| 29 | G-Box | cis-acting regulatory element involved in light responsiveness | CACGTT | 1 | 0 | 1 | 0 | 0 | 2 | 1 | 0 | 0 | 1 | 1 | 1 | 0 | 2 | 2 | 1 | 0 |
| 30 | GATA-motif | part of a light responsive element | GATAGGA | 2 | 0 | 0 | 1 | 2 | 2 | 1 | 0 | 1 | 0 | 2 | 3 | 1 | 1 | 2 | 0 | 2 |
| 31 | Sp1 | light responsive element | GGGCGG | 1 | 0 | 0 | 0 | 0 | 0 | 0 | 0 | 0 | 2 | 0 | 1 | 0 | 0 | 1 | 0 | 1 |
| 32 | TCT-motif | part of a light responsive element | TCTTAC | 1 | 1 | 0 | 2 | 1 | 0 | 1 | 3 | 2 | 2 | 0 | 0 | 0 | 1 | 0 | 0 | 1 |
| 33 | chs-CMA1a | part of a light responsive element | TTACTTAA | 1 | 0 | 0 | 0 | 0 | 0 | 0 | 0 | 0 | 0 | 0 | 0 | 0 | 0 | 0 | 1 | 0 |
| 34 | AE-box | part of a module for light response | AGAAACAA | 0 | 1 | 0 | 1 | 1 | 0 | 1 | 1 | 0 | 0 | 0 | 1 | 0 | 1 | 0 | 0 | 0 |
| 35 | GT1-motif | light responsive element | GGTTAAT | 0 | 2 | 0 | 0 | 1 | 0 | 0 | 0 | 1 | 1 | 1 | 0 | 0 | 1 | 0 | 2 | 0 |
| 36 | MRE | MYB binding site involved in light responsiveness | AACCTAA | 0 | 1 | 0 | 0 | 1 | 1 | 0 | 0 | 0 | 0 | 0 | 0 | 0 | 0 | 0 | 0 | 0 |
| 37 | ACA-motif | part of gapA in (gapA-CMA1) involved with light responsiveness | AATTACAGCCATT | 0 | 1 | 0 | 0 | 0 | 0 | 0 | 0 | 0 | 0 | 0 | 0 | 0 | 0 | 0 | 0 | 0 |
| 38 | TCCC-motif | part of a light responsive element | TCTCCCT | 0 | 0 | 1 | 0 | 1 | 2 | 0 | 2 | 0 | 0 | 0 | 0 | 1 | 0 | 1 | 1 | 0 |
| 39 | AT1-motif | part of a light responsive module | AATTATTTTTTATT | 0 | 0 | 0 | 1 | 0 | 0 | 0 | 0 | 1 | 0 | 0 | 0 | 0 | 0 | 0 | 1 | 0 |
| 40 | ATCT-motif | part of a conserved DNA module involved in light responsiveness | AATCTAATCC | 0 | 0 | 0 | 1 | 0 | 1 | 0 | 1 | 0 | 1 | 0 | 0 | 1 | 0 | 0 | 0 | 0 |
| 41 | LAMP-element | part of a light responsive element | CTTTATCA | 0 | 0 | 0 | 1 | 0 | 0 | 1 | 0 | 0 | 0 | 0 | 0 | 0 | 0 | 1 | 0 | 0 |
| 42 | GA-motif | part of a light responsive element | ATAGATAA | 0 | 0 | 0 | 0 | 1 | 0 | 0 | 0 | 1 | 0 | 0 | 1 | 1 | 0 | 0 | 0 | 0 |
| 43 | I-box | part of a light responsive element | GGATAAGGTG | 0 | 0 | 0 | 0 | 0 | 1 | 1 | 0 | 0 | 0 | 1 | 0 | 1 | 0 | 1 | 0 | 0 |
| 44 | GATA-motif | part of a light responsive element | AAGGATAAGG | 0 | 0 | 0 | 0 | 0 | 0 | 0 | 2 | 0 | 0 | 0 | 0 | 0 | 0 | 0 | 0 | 0 |
| 45 | GT1-motif | light responsive element | GGTTAA | 0 | 0 | 0 | 0 | 0 | 0 | 0 | 1 | 0 | 0 | 0 | 0 | 0 | 0 | 0 | 0 | 0 |
| 46 | ATC-motif | part of a conserved DNA module involved in light responsiveness | AGTAATCT | 0 | 0 | 0 | 0 | 0 | 0 | 0 | 0 | 0 | 0 | 1 | 0 | 0 | 0 | 0 | 0 | 0 |
| 47 | Box II | part of a light responsive element | CCACGTGGC | 0 | 0 | 0 | 0 | 0 | 0 | 0 | 0 | 0 | 0 | 1 | 1 | 0 | 0 | 0 | 0 | 0 |
| 48 | ACE | cis-acting element involved in light responsiveness | CTAACGTATT | 0 | 0 | 0 | 0 | 0 | 0 | 0 | 0 | 0 | 0 | 0 | 0 | 0 | 0 | 0 | 1 | 0 |
| 49 | AAAC-motif | light responsive element | CAATCAAAACCT | 0 | 0 | 0 | 0 | 0 | 0 | 0 | 0 | 0 | 0 | 0 | 0 | 0 | 0 | 0 | 0 | 1 |
| 50 | Gap-box | part of a light responsive element | CAAATGAA(A/G)A | 0 | 0 | 0 | 0 | 1 | 0 | 0 | 0 | 0 | 0 | 0 | 0 | 0 | 0 | 0 | 0 | 0 |
| 51 | 3-AF1 binding site | light responsive element | TAAGAGAGGAA | 0 | 0 | 0 | 0 | 0 | 0 | 0 | 1 | 1 | 0 | 0 | 0 | 0 | 0 | 0 | 0 | 0 |
| 52 | AT-rich sequence | element for maximal elicitor-mediated activation | TAAAATACT | 0 | 0 | 0 | 0 | 0 | 0 | 0 | 0 | 1 | 0 | 0 | 0 | 0 | 0 | 0 | 0 | 0 |
| 53 | 3-AF3 binding site | conserved DNA module array | CACTATCTAAC | 0 | 0 | 0 | 0 | 0 | 0 | 0 | 0 | 0 | 0 | 0 | 0 | 0 | 0 | 0 | 1 | 0 |
| 54 | Box II -like sequence | regulatory element | TCCGTGTACCA | 0 | 0 | 0 | 0 | 0 | 0 | 0 | 0 | 0 | 1 | 0 | 0 | 0 | 0 | 0 | 0 | 0 |
| 55 | CAAT-box | promoter and enhancer regions | CAAAT | 40 | 24 | 25 | 33 | 44 | 31 | 32 | 33 | 48 | 35 | 30 | 30 | 36 | 32 | 33 | 28 | 34 |
| 56 | MYB | flavonoid biosynthetic pathway | CAACCA | 0 | 1 | 3 | 1 | 3 | 2 | 2 | 3 | 2 | 4 | 2 | 5 | 4 | 5 | 6 | 4 | 3 |
| 57 | TATA-box | around -30 of transcription start | TATTTAAA | 31 | 12 | 7 | 60 | 55 | 13 | 78 | 0 | 68 | 5 | 15 | 33 | 8 | 20 | 27 | 54 | 20 |
| 58 | HD-Zip 3 | protein binding site | GTAAT(G/C)ATTAC | 0 | 0 | 0 | 0 | 0 | 0 | 0 | 0 | 0 | 0 | 0 | 0 | 0 | 1 | 1 | 0 | 0 |
| 59 | AT-rich element | AT-rich DNA binding protein | ATAGAAATCAA | 0 | 1 | 0 | 1 | 0 | 0 | 1 | 0 | 1 | 0 | 0 | 0 | 0 | 0 | 0 | 0 | 0 |
| 60 | TATA | transcription promoter | TATAAAAT | 0 | 0 | 1 | 0 | 0 | 0 | 0 | 1 | 1 | 0 | 0 | 0 | 0 | 0 | 0 | 2 | 0 |
| 61 | motif I | root specific | GGTACGTGGCG | 0 | 0 | 1 | 0 | 0 | 0 | 0 | 0 | 0 | 0 | 0 | 0 | 0 | 0 | 0 | 0 | 0 |
| 62 | A-box | regulatory element | AATAACAAACTCC | 0 | 0 | 0 | 0 | 0 | 0 | 0 | 1 | 1 | 0 | 0 | 1 | 1 | 0 | 0 | 0 | 0 |
| 63 | CCAAT-box | MYBHv1 binding site | CAACGG | 0 | 0 | 0 | 0 | 1 | 0 | 0 | 0 | 0 | 1 | 1 | 0 | 0 | 0 | 0 | 0 | 2 |
| 64 | telo-box | Myb-related telomeric DNA binding motif | AAACCCTAACCCTAA | 0 | 0 | 0 | 0 | 0 | 0 | 0 | 0 | 0 | 0 | 0 | 0 | 0 | 1 | 0 | 0 | 0 |
| 65 | Myc | Unkown | TCTCTTA | 0 | 2 | 0 | 1 | 0 | 0 | 1 | 1 | 1 | 0 | 0 | 1 | 1 | 0 | 0 | 0 | 0 |
| 66 | WRE3 | Unkown | CCACCT | 1 | 2 | 1 | 1 | 3 | 2 | 0 | 2 | 0 | 0 | 1 | 1 | 0 | 0 | 3 | 0 | 0 |
| 67 | box S | Unkown | AGCCACC | 0 | 1 | 0 | 0 | 0 | 0 | 0 | 0 | 0 | 0 | 0 | 0 | 0 | 0 | 0 | 1 | 0 |
| 68 | dOCT | Unkown | CACGGATC | 0 | 1 | 0 | 0 | 0 | 0 | 0 | 0 | 0 | 0 | 0 | 0 | 0 | 0 | 0 | 0 | 0 |
| 69 | Myb | Unkown | CAACTG | 0 | 0 | 2 | 3 | 2 | 2 | 1 | 1 | 3 | 2 | 0 | 5 | 2 | 1 | 0 | 1 | 4 |
| 70 | MYB recognition site | Unkown | CCGTTG | 0 | 0 | 0 | 0 | 1 | 0 | 0 | 0 | 0 | 1 | 1 | 0 | 0 | 0 | 0 | 0 | 2 |
| 71 | CARE | Unkown | CARE | 0 | 0 | 0 | 0 | 0 | 1 | 0 | 0 | 0 | 1 | 0 | 2 | 1 | 0 | 0 | 0 | 0 |
| 72 | MYB-like sequence | Unkown | TAACCA | 0 | 0 | 0 | 0 | 0 | 1 | 1 | 1 | 1 | 0 | 0 | 1 | 1 | 1 | 1 | 2 | 1 |
| 73 | AP-1 | Unkown | TGAGTTAG | 0 | 0 | 0 | 0 | 0 | 0 | 1 | 0 | 0 | 0 | 0 | 1 | 0 | 0 | 0 | 0 | 0 |
| 74 | CTAG-motif | Unkown | ACTAGCAGAA | 0 | 0 | 0 | 0 | 0 | 0 | 0 | 1 | 0 | 0 | 0 | 0 | 0 | 0 | 0 | 0 | 0 |
| 75 | ABRE2 | Unkown | CCACGTGG | 0 | 0 | 0 | 0 | 0 | 0 | 0 | 0 | 0 | 0 | 1 | 0 | 0 | 0 | 0 | 0 | 0 |
| 76 | ABRE3a | Unkown | TACGTG | 1 | 0 | 2 | 0 | 0 | 1 | 0 | 0 | 0 | 1 | 0 | 0 | 0 | 0 | 0 | 1 | 0 |
| 77 | ABRE4 | Unkown | CACGTA | 1 | 0 | 2 | 0 | 0 | 1 | 0 | 0 | 0 | 1 | 0 | 0 | 0 | 0 | 0 | 1 | 0 |
| 78 | TCA | Unkown | TCATCTTCAT | 1 | 0 | 0 | 0 | 1 | 0 | 1 | 0 | 0 | 0 | 0 | 0 | 0 | 0 | 0 | 0 | 1 |
| 79 | AC-I | Unkown | (T/C)C(T/C)(C/T)ACC(T/C)ACC | 1 | 0 | 0 | 1 | 1 | 0 | 0 | 0 | 0 | 0 | 0 | 1 | 0 | 0 | 0 | 0 | 0 |
| 80 | AT~TATA-box | Unkown | TATATA | 1 | 0 | 0 | 7 | 13 | 0 | 6 | 4 | 8 |  | 1 | 1 | 0 | 1 | 0 | 1 | 0 |
| 81 | AAGAA-motif | Unkown | GAAAGAA | 0 | 1 | 1 | 2 | 1 | 2 | 1 | 1 | 0 | 0 | 1 | 3 | 1 | 2 | 1 | 7 | 2 |
| 82 | F-box | Unkown | CTATTCTCATT | 1 | 0 | 0 | 0 | 0 | 0 | 2 | 0 | 0 | 0 | 0 | 0 | 0 | 0 | 0 | 0 | 0 |
| 83 | as-1 | Unkown | TGACG | 1 | 1 | 0 | 0 | 3 | 0 | 2 | 0 | 0 | 2 | 1 | 0 | 0 | 0 | 0 | 1 | 2 |
| 84 | Myb-binding site | Unkown | CAACAG | 1 | 0 | 2 | 0 | 1 | 0 | 0 | 0 | 1 | 4 | 2 | 1 | 2 | 0 | 1 | 2 | 1 |
| 85 | Unnamed__1 | Unkown | CGTGG | 1 | 1 | 3 | 0 | 4 | 1 | 1 | 1 |  | 3 | 4 | 2 | 0 | 2 | 1 | 2 | 0 |
| 86 | Unnamed__2 | Unkown | CCCCGG | 0 | 0 | 0 | 0 | 0 | 0 | 0 | 0 | 0 | 0 | 0 | 0 | 0 | 1 | 0 | 0 | 0 |
| 87 | Unnamed__10 | Unkown | TCCACGTAGA | 1 | 0 | 0 | 0 | 0 | 0 | 0 | 0 | 0 | 0 | 0 | 0 | 0 | 0 | 0 | 0 | 0 |
| 88 | Unnamed__12 | Unkown | TCCACGTAGA | 1 | 0 | 0 | 0 | 0 | 0 | 0 | 0 | 0 | 0 | 0 | 0 | 0 | 0 | 0 | 0 | 0 |
| 89 | Unnamed__14 | Unkown | TCCACGTAGA | 1 | 0 | 0 | 0 | 0 | 0 | 0 | 0 | 0 | 0 | 0 | 0 | 0 | 0 | 0 | 0 | 0 |
| 90 | Unnamed__4 | Unkown | CTCC | 15 | 13 | 15 | 4 | 17 | 16 | 19 | 12 | 9 | 22 | 12 | 20 | 11 | 14 | 22 | 5 | 12 |
| 91 | Unnamed__6 | Unkown | TATAAATATct | 1 | 0 | 0 | 0 | 1 | 0 | 0 | 1 | 0 | 0 | 0 | 0 | 0 | 1 | 0 | 1 | 0 |
| 92 | Unnamed__8 | Unkown | TCCACGTAGA | 1 | 0 | 0 | 0 | 0 | 0 | 0 | 0 | 0 | 0 | 0 | 0 | 0 | 0 | 0 | 0 | 0 |
|  |  |  |  |  |  |  |  |  |  |  |  |  |  |  |  |  |  |  |  |  |


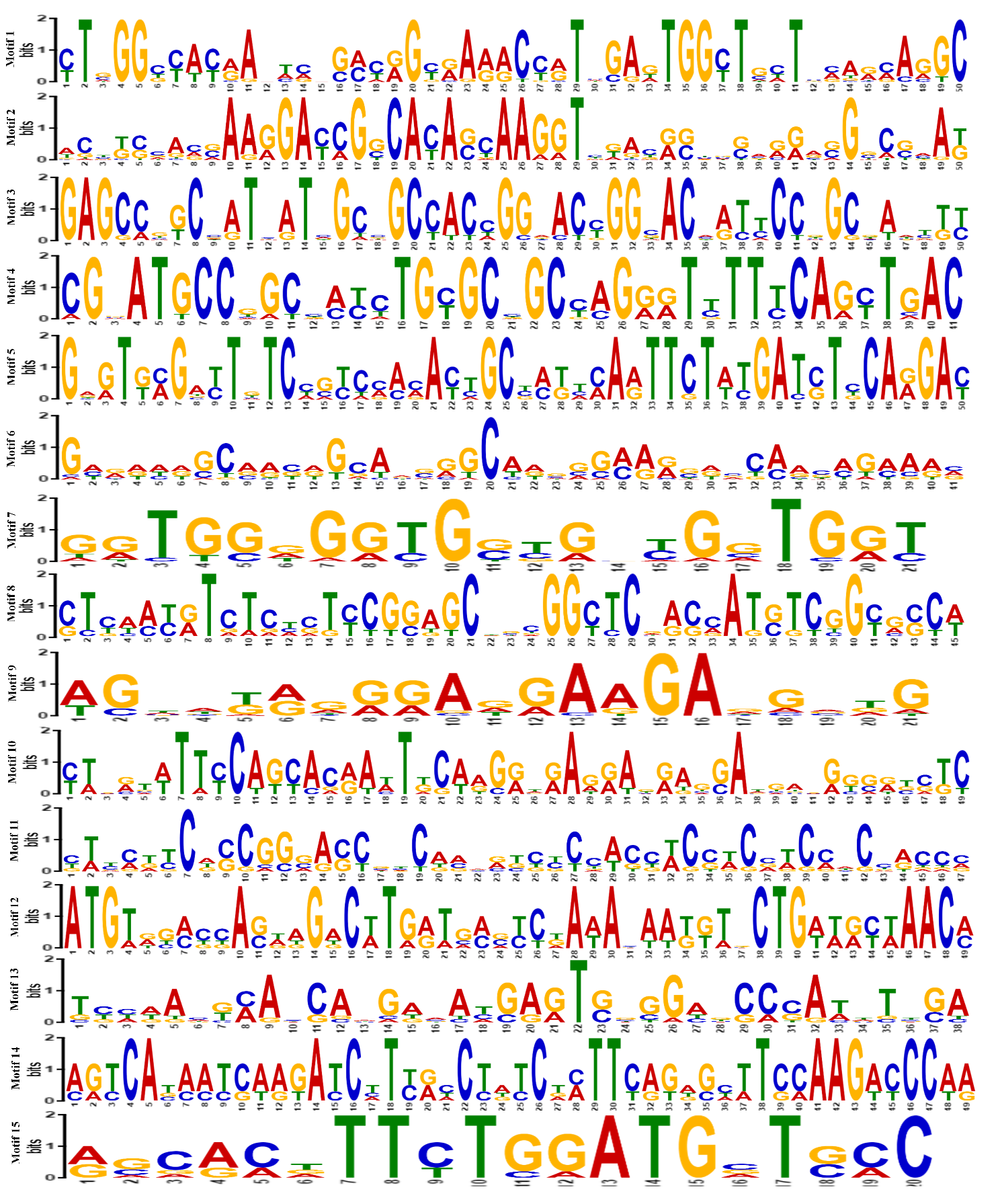


**Figure S1**. Sequence signs for 15 motifs of TCP domains using the MEME online software. MEME motifs are displayed by stacks of letters at each site. The total height of the stack is the ‘information content’ of that site in the motif in bits. The height of every letter in a stack is the probability of the letter at that site multiplied by the total information content of the stack. The *y-axis* denotes the bits of every letter and the *x-axis* denotes the width of the motifs.


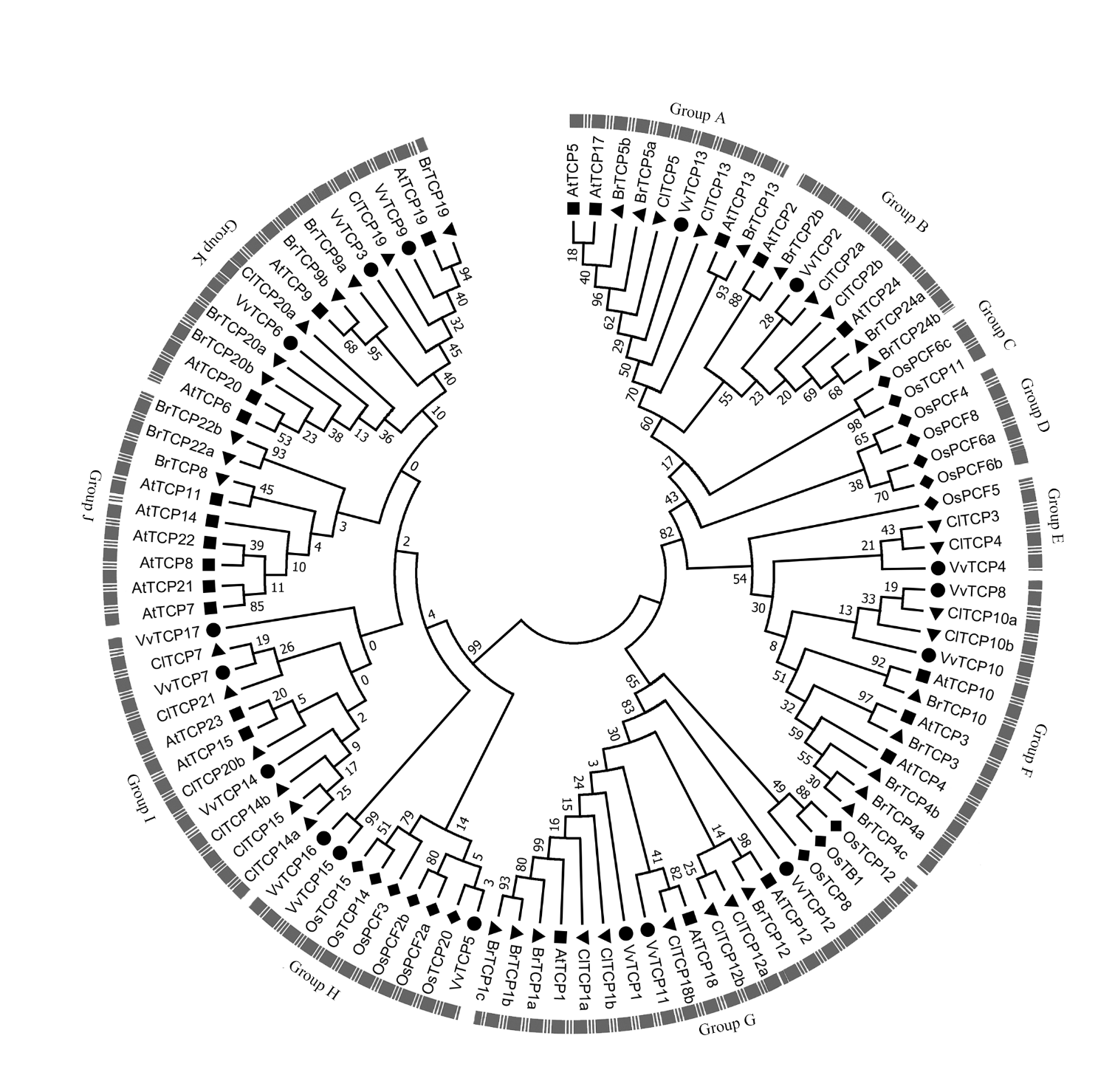


**Figure S2**. Phylogenetic analysis of TCP gene family among *V. vinifera, Arabidopsis, O. sativa, C. lanatus* and *B. rapa*. The phylogenetic tree was constructed using MEGA 7.0 software using Maximum-likelihood (ML) method with 1000 bootstrap replicates. Different shapes represented the TCP genes of different species, Data Set 1. The promoter sequences of 17 VvTCP genes in grape (*V. vinifera* L.)

**Data Set 1.** The promoter sequences of 17 VvTCP genes in grape (*V. vinifera* L.).

>VvTCP1 (*2000 bp)

CAACAGTCCAACGCATAGGTCTATTTTCATTCCATATTTGCATTCATCATTAATTATAATTGTTAGTTGTGACAAAAATGGAGTAAATAGGCCTAATTCTCCATCAAATAACTCTACTGTCTGTTTTTTTTTACTCCAAACATCCTCAAATTCTCTAATCAAAATAATCACTTACCATGTAGATGATGCAGTGCCCACTGATTGCAGTCTCATATATGTATGACTTCGATTGTTACAAGATTTTACAGATGATATGACCATGGCAATTACAAGCCACTCTTTTTACTTAAGAAAATGGAAGCAATATTGTTTTATGCGCAATGTCCTCACTTTCACCCACCATGTGCTATCTTCACCTTTCTGCAGTTACCCATAGACATCCTGTCCTTTGAAAACAACATCAGAACCACCCACTGCTATTAATGTCATTGACTCCTTGTAGCTATGCTCCATCAAGGAAAGAGGAAGATAGGAAGCACATGTACTACCTGAAAGGATATATAATGCAAGAAGAGGGCTTCTCTAGAATTATTAGTGGGGGGCGAAATAAGGATGGATGAATGGAAACATAAATCTAGGTTACAGAAGTATAGAATTAGCATATAACATGTGGTCAGTATTACGCTGACATGGTTGGTGGGGAAACCTAGGGTTCGGGAGATGAGCTCGTGAATGGCAGAGAGATACCACTATACCAGTACAAGCAACAACAGTAGCTCAGATAATTAAGAGTGACCTTGCTGCTGTGTTGAGTATTGGAATAAAAAAACCTAGAAATCCAAACCCTAATCAGAACCAAGTGTTTATAGGGATTCGACTAGAGATGTACTTTCAAGAACTGAAATAAAATAAAAAGAATCTAGACACAATTTAAGACCCAAGTCACCCTAAACAACCAAAAATAAAATAGAAGAACAAAAATGAAGGTTCTTAAAAATAATTGGACCTCCTCCTTCAGCATTTGAATCAAACCAGAGGTATGCATTGATGAAACCAAACATCTACAATAAGGAACTGAAATCAAAAGAGACCTTATAAAGGTGCCATGGCTTCACCATGGGTTTTGAGGAGGCAAATGTTTAAAGAAAGCATTGTAGAATGATCCCAGACTACAAACAGAAGAAAACTGAGATGCATTTCAATGGGCTAACAAGGAGATGGGCCTTCTCTTTTTACTGTACTCCTTGAATTGAGAGAGAGAGAGAGAGAGGAGAGAGAGGAGAGAGAGGTTTTAAGTGAAAGTGACGGCCAGAGATGGGTGTTCTTTTAAGTGAAAGCGACGGCACTGATAAGTTCAGTCAAGGAGGTGTGGAAGAAAGGGTTGATCACCAATTTACATGGTGTTACGGGCGGCCCAAGCAGTAAGAATTGTGTCCTTTTTAGACAGGAGAACGTGTAGCCTCTACAATATCTGTGTCTACGTGCACGGACAAGAGACACCACATAAAGGCCACGGCCTTTAGAAACCCTAAACGTTCTTTATTTGTTCTCATCCTAGACGTAGAAATATTAATTGAAAATATTGGAAATGAAAACCCTAGATGTACCCGTTTATCATTCTTCAGAATTCTTCGATTCAAATGAAGGGTGAGCCTAAGCTTAGCTCATTGCATTCCCCTTTCTTGGAAAAAGCTACATAAGTATGTCATATCATGTATAAGTGACCTAGACCGGTCAAATATATTTATTTAAAAAATATTAAATATAAATATAAATATGCCAAAATCAAACTATAAATATCAAAATAAAATCTTAGATAAAAAATACAATTTTAAACTAATTTAAAAAATAAATAAATAAAAGACTACTTTAAATGATGGTTTGGCAATATGCCAATAAGCTACGTTTTATTTGGCAAAAATTCCTCAGATCCACAAACAAATAACGGGCCTAAAAGTGATAGGGTTACATCAATCCCTGTCGAGAGGCTTTTGTCATCACTCTCTATTTGCTGCCCTAAAATTTAGTGGCATAAACTATTTATTTCATTGAATTAGGTC

>VvTCP2 (*2000 bp)

ACCTTTCACCAAGAAAACCAGCACAAGAAGAAGACCCCTTTGCCTTGCTTCCTATTATGACTTTGTCTACCTCGTTGCTTCTAGGGATAGAGAGAGTTTAGAGAAACAAAGGGGTGGCAGTTGGAGTGGTGGGAGTGCAGGTGGGGTGGTGTGTCCTTTGCATTGATGACGAATACTCAGAAGTACTTTGGCTGTGTTGTGATAAAAAGGCCTTTTTTTACGGTTCGATATATCGCAGTCCAAGCCGGAAATGTCAGCACTCTTCGTTTAAGCTTTTTGCAGTGCTTTCAAAAGCAAAGCCTGAAATGTCTCAGACTCACTCAGCCCCTGCGAACCTAAGAGAGAGAAGCATCGGATGCTTAATACGTACACTGTTTTAACACTAACATAAACACATCCCCACACCCATTTTATCAACATCTCATTTGACTTAGGCCTAAAGATCTGTTTTTAAAGATAATCAGCTTTGGAAAAGCGAAAAATAAAAGCTTACTACTTCTCACCTTGATCTGATGAGCTTTTTTCTGCTTCAGATTTAAGCTATATAGCCTGCAGAGTCGGCAATTGCGTACAGGTCGCCGATCTATGTCTTTTTTCAACTCCTTCGAATTTAAGGCTTTCAATGACCTAAAAATGGCACACATTACATTACTACTGTGCCCAGAGGAAAGAAAACAATAATCATAAAATATACATCGTTTCTGTTTTCTAAATTACAGACACCAAATCTTCAGCAATATTGACTGCGATTTTGAGGTTTTAAATTATGGTAAAAATGGCTGAACACTCTTTTGTTTTTCCTATGGTTTTCTTTGTTTTCAATGCGAACTAGAAAACACGGATCTGTGCTCATCCTCCACATGGATCTGTCGCTCTGAAGAACAATTACTGTACAAACCGAAACAAAATTTCGAGTATTCGTAACTCAAAATACGCAAAACAGCTCCATTTCAAAAGCTTAAAAAAATTGAACCAAAAAATTTTGTTTTAACCTGCGATTTCCAGTTCCTACGCAGCAAAACAAAGAATCAAACATTATTTGAAGAATGACAGGTGGGAATAAGCTCCATTTCAGAAAAAAAAAATTTAAGGAAAAAAAGGCAAAGAAAATAAAAAGGAAAAAAACCAAACAAACAACGAACGATATGGAGCCGGATCGGAGCGGTTTCAAAACGAGGAGACAAAGAAGACCTTCTTCGGATTTCGGGAAGCGAAGTTGGCGTGCGGTTGTTTCCTACGGTTGCGAAAGCAGTCTTCTCAGTAGAAAAAGACTCTCTCTCTCGAACTCGCTATGTATCGCTAAAAATGCACTAGCACTTGTTCAACTCCTCGTCCCCTCCCACTAGTCCAAGCCTTGAAAACACCAAAACCGCCATTAACGAAGCTCGAATTCACCTGAAACTGAGCTGAATTGCGACAGAGATTGAAGGAAGTCGTGGATGCGTGTTTCTCTCACTAGAACTGATATTTCTTTTTCTTGCGTTCCAGTTTTAAGAGAGAGGAAGAGAAAGAGAGATGAGGATTGTTTGGTGGCTGAGAAAATGAAATCCAAGTGTACAGGAATTTTTGAGAAATCAAGATACGAAGAGAGAGAAAGAGAGCTCGCGGTTCTATGCCCTAAGGCTTCACCATTCGAGCAATCACCGGGCTTATCGGGAAAGGACAGACACAGCGCCGTTCCCACTTTAAATATGTCTCTCTCTCCCATTTGCCCCTCTCTCTTTTCCATTTTACTCTTTTGCCTCCTTTCCTTCATTTTTACTTCCCTTCTCTTTTTTCTCTCATATTTTACTTCTTACGCCCTTTTTCTTCCCGTAAACAAACAATCAAAGGATGCTTTGTTTTCTATTCATAAGTAACCCATCCCTTCACCGTATGGTTAATTTTTATATCAAATTATGTTAGGTGCTTCTTATTATTCTTTATTTCTTCAAAAATCAAATGATACTACTTTAAGTACGCCATATAATTTTTTTTTTCTAAATATTTTTAATGAAAAT

>VvTCP3 (*1276 bp)

ATTTAAGTTGTCTTTCACTTTTACTCAAATACCAAAACCCCATTTTGTTGGAGGATAATTTGTCAGTCTAAACTCAAAAGTCGTCGAACTTTTTATTCTTTTTCAAACTTGAGAGCTAACTTCAGTTGCAAGTTGCAACACAACTTTACCGTCGTTAATGCTCATGCTCTGTTTCAACAGAAAAATTAAATCTCCATAAAGTAGGAATCAGCATTCTTTGATCAAGGCAAGTATGATTAACATCTTAATGCTTTATCCTCTATTGGAAGTACTTGCTCATTATGCCTTGAAAGCCGCCTCCGAAAAGGCAACCTTTGGAAGTAGAGAAAAGTAATACCCATCTCCTCCGTGAAATTGATCGAGTCACAAAAATTCCCCCTTGTGCGTGGAATTTAAGAAACTGAAGAAGAGAGAAGAGAACTGAACTTGGGATGTCATCTTCTGTGTGATGTCTTTGGTTCAGAAAAATGCAAAACTTGACACCCAAGTCAAATGGCTGTAATAGGAATAGGATGGGTGCTGAGGAGAGTTGTTGTTTGGTTGGGGCCCCAAATGATGAAAAAACAGAAACGGAAAAGAGCTTCAAACGAGTAATCCCCAGGACAGGTGAGAGGAACAGGAGTTGGGCGACATAAAATTCTATGGAGTCGGAGAGTGTAGAAAGAAAGAGATGGGACAAGTGGTCCAACGCTTTTTGTAGGAAAACAAAACATGAAAGGATGTGAGACAAGTGGCTAAATGGTTGTAGGCCGCAACAATGTCGGGGTTGAGCAAGGCAAATAGTGCCGCTATTCATAAACGTGAAAGAGTGATAAGAGGAACCAACAGATGTGGCCCCTTTGGTTTCTAGACAACAAAAGGTGGTGGTGGTGGTGTTTGGTAAGGCAATGGGTGTTTATCAGCATTCAAATTTGTGTTCCATTTGCAATTATACTTGTGGGGAGTAGCTCAACTTTAGGGAGGAGGAACCCAAAGAAAAAGAAGTTCCAATATCATATAAAATTTGACAGATTTCAAGAGTATGAGGACAATTTTGTGCTCAAGAAAATGCCAAAAACCAATTAGCCATTGTTTTTCTTTTCTTCTCGAAGTTAAAAGGGAGATTTAATCGATATTTCACCTACAAGTTAGGAGAAAAGGGGAGGAATCTAAATGTACCAATCCATGGCCAAGTGCAACTGTGAGGGTGGGGTAATACTAATGCTTTTCCGGTAGTACGTGGACCACCAAAGGTACGTGGTGGCAACCAATCAAAGCCATTTATTGAGTCAGAAAA

>VvTCP4 (*2000 bp)

ATAACTTAAAATTTAAAGTTTATAATATGATATTAAAGATATGTTATTTTCTTTTCCCCAATATTAATGTTAATTCTATCTTATTTATGACTTGATCGGTTTTATAAATTATATATTTAAACAACTTATAATTTAAAAAGAAATTTGATATTGAATGCCTGAAAATATTACTTTTTAATGAAATTTTATTTTAAGACCCGTAAAAAACTATTTCATATTATATGAAACTTTTGTAATGATAGTACCGTCTTTTGAAAATTGTGGGTTTAGTTCGACATTAACTTCTAGATAAAGTACAAAGACTATATAGTAAATAGAAGACAAAATCTTCAATATATTTTTGAATAATTCTTTTAAAAAACAAAAACTTTTAATTTAAGAAATAGCTTAATATGACAAACATAACAATATTCATTTTTCAAATATTTTTTTAATACTTAAAGTTATTTCAAGAATAATTAAATTATTTCTTATTAAATTAAATTTTTATTGCATCAAGATTGATCCTTAAAATTATGACATACAAAGGTGAAATAAAAAATAAAAAATCATGTAAACCCAACCCTTATAATTTTTTTTTTTAATAATATTTAGCATTATAAATAATCATAAATCCAAATTTAAGTTGTGAATTTATGTCATGGATTTAAATTCATAAATTTATATAACTCATTTGGGTAAATTTAGAGTTATTTCCTCAAAAAGATCCTTTTAATTTGATTTTTATTTTTATTTTTAATTTTTTTTATATTTTTTTGTGATTAATAAAAAAAACATATATAATGTAAACCTAAAAGCATGCTCTCTTATTGGGTATTGCTTTGGATCTCAATTTAAATTTTAATTGCCAAATAAAACTAATTATGAAAATATTATTTTTCTTAATATTTTTTTCTTTTGGTTATGTTTACTTTCTAGAAAATTTGAGAAAATGTAAATAAAAAGGAAAAATAAAAAGAAAGAAAAATAAAAATTAAATTTAAAATTATTAAATTATTTTTAAATACTTCTTTAAACTCATTTAACTTATTTTTCTTTATTATGTAAAAATTAAATAATTTTAAAATATATAAATTTTTAATTATATTTAATTTCCTTTCATATTTTCTAAATCAAATTAAATACGAAAAACTAATGTTTAATATTTTTTTTTCTTTTTTTAATACTTTCCTAAAGCCAAATTTTAAAACCAAACATAACCCTAATGTTTAAAGGTAACCTTAATTATCAAATCAAAAGTCGAAGTAAACACAGACTTAAAACTATCCCAAGAAGAAGGGACTTGGAATTCGATCTAACCCACTTAAAAAAATCTTCAATGAATGCCAATAATCATAAATTGGAAATCAATACTAAAAGGGGCTGTTTGGGGACAGAGAAACCTAGAAATATGATAGGAATTCTTACATTAATATCACATGTCTCTGATAAAGATTTTATCACCAAAAAGTGGCAACAAAATAAAGTAGAATGGATGTTTAAGGATTGGATTAGCCATTATAGAATTTACTGAGCTTTTACAAACATGTGGGACACAAACCATTTATTACAAAATACAGGGGCCACCCATGAAGAGATTGCCTTGACCAAGGACACCAAATTAAAAAAATAATAATAATAATTAAAAAAAAGGGGGAAAAAAGAAAAGAAAAGAAAAGAAGACAGGGCCTTGAGATGCTATGTACAGTAATCAAAGAAGGCAGAAAAAGGTGGGTTGGGAGATGGAAAATTGAGGGAAAGATGATGTTGTTTCTTTTAAGTGAAGCAATAAAGAAAGAGAGAGAGAGAGAGAGAGAGAGAGAGGGGAGAGAAAGAGAAAACCCAGAAAAAGGCTGTAAGAGGTCGCTTTCAAAAGTGGTGGTAGTGGCATTTGGGAGGGGGGAGGGGATCTAATAATATATATCAAAAGCATCCATCCAGAGGAAAATCCACTAAATCCCATATGACCACACAGTTGTAACTGTGATGTAAAAACCTGAAAATAACTGAATGGGAAGTGA

>VvTCP5 (*2000 bp)

ACGAACAACCATATGAGAACTTAATGAATATGATTTGTTCATTTGAGTCTGATGAACTAATACTTTATTTGAAAATCGTTCAATCTACAAGTTGAGACAAAACTCAGTGTCTCCTAGATCTTTTATTGCAAATTTCCTTTTTAAAAATTCAGTCGTCACTGTGAGTTTTCTAGGAACTCCAACAAGACTCAAATCATCTATATATATTGCAATAATTGCAAATTCGATCATTAATTTCCTAATGAATATATATAGATAAAGAGAATTATTTATATATCATATTTTCAATAAATATTAATTGAGGTGATTGTATCACATGAATCTGAATTGTTTCAATCCATACATGTGATTTTTCGAATTACTTATTTATATATATATGTAAAATAAAAAATTCTTTAGAATCGTAGAAATTATCTTATTCTAATAACATGTTATGAATCATAAAAGTTAAGGGGCAAAAAAAAAGAAAAAAAAAGAGAGAATAAGTTCATATTTTTTTATTAATTCAAAGACCCTTATTTATTTATAGGATAGGAGGCATTATTACTAAAATAACAATAATATAAACATCGTAAGAAGCAAATAGCCATTAAAAAATGTGTTTGGTCATTGAATCACATCAAAGGTCATTTCCAGCTGTAGGATGGGAAAGAGTCATTGGGAGAGACAGGAAATGAAATCCCCAGTTGACGATCGGACCGTCTTTCCACATTGTTTTCCATTCTTCGCTGCTCGAGATTGGCATAAATGGGCAATAATATGTCTATAAAACACAACACATCTCCTACCCTCATTTATCGTCATCTCAGTCCTGATTTATCATATATAACCTAAATTAGGCTGATGGGTCAATACAAGTACGGTTCCAGACATAAATATCTTTTAATTTACCAAAATAATAAAAGAAAAATTATGATATGTCAGAAACAATGAAGCTTAAACAACAGAGGGAAGGTTGGCATCTTTTGCTTTCACATTACTTTAAGATCAATAAATTTGTCAACAAAACTGCCTTATTTATAAAGGAACCCGAGATTCTGGATTTTTTTTTTTTCCAAGTAGGGGAAAAAAAAAAACGATTTGAAATAGTGGAAGTCACAAACTTTGAATTTTTTCCGAAATTTTGGAAATGGGTGGATATACTGATTAAAGGACCTTTAATGAAGCAGGGGAGGGAGACATATTGAAGACACAATTTCCGAGGGTGGTCAAAGCCCAAAAGCATGAGAGGAGAGGACTCATCAAATTTGAGAACACACCACTCAACCCAAGCTCTCCAGAGGGCGTGGCTCGTTAACCCAAGCAGTTGGTGGGGTTCACGGGAGTCCACGCCCACACCCATGACCCACACCACACGAGTCATGAGAGCGTGGGCGTTCCTTCACAAATGGAGCTGAGTAATATTGATTTGAGTAGTGGTGACCAGCGACCAGCGACCAGTATTGACCATCCATCATATCTGAAATGACTCACAGGTAGGATCCACCCAGGATTCAACATGGGGGTCCCCCACCACCCAAATCCTCCGTACAACACAGAATGCACAAAGCTCAACCAATTGTATGTCTCCACCTCAACCCAAAATAAATATCCAACTTAATAGCATTGTTTTTTGTAAAATCCAAAAATTTGAGGGGTTGAAAGAAAGGGAATTTTAGAAGGGGAAGAAGGTGGAGACAGGAGAGAGGCTCATCCCCTATCTCAATTATCGATTTCTCAAAAACCCACCCACCTACTACTGGTACTGCTCTCACTCTCACATGGGCACATGGGCCCAACGGAGGCCCATCTTATATATAAGGGTGTATGCTCCTCGTGGGGTCGATAGAATCCCCAAACACTGACTTCATACCTCGCTTTCTCTCTATCTCTCTCTCTTTCTCATCTATTAGACTATTACCCTCTCTTGGCAGAAACCCTAGAGTTCTGTCCTTTTTTGATTCAAACCCTAAAACCCCCTCTTTTCAGATCATTAGTCTTTTATCTGTATAGGTCTAATC

>VvTCP6 (*2000 bp)

ACAAATAGAAAAGAGAAAAGCAACTTTGGACTGATTACTCCACCTAATCCCCCTGCTTGCATGACTCCATGGCCATCACTACATAAATGATAACCTCTGATTTTACACTTCATAGATTTTGCACAGAATGGGCACATCCCCTCCATAATCAGCTTGTGCTATATGAAAAAAACCCAAGTATGGGACTTATATTTATCCACAGGAAGGGGCTAAACCTATGATGAATGATAGAATATTTTTTTCTTCTTCCCAAAAGCGTGATTTGTGGGAGGTTTTGACATTTCCTTTTCATTCTACTTAACAAGGCACCACACAAGACTAAAGTTCTCATATTCAAAACTATTTCACACCAGGATATGGGAATAGATTTGTTGTTTGTGTGTCCCAATGCACTCTATTATGATTAAAAGAAGAAAAAAGAAAAAGCCATTTTTAAAAGGGAAAATTCTGATAAGGGAATAATACATATTGAATTTTGGAGAGCAAGTATATTGAGATTGGAGAGAGAGTTAAGAACTCCATCAAATGTGAGTTGTACAAATGAGGTACACAAGCATCCACCAGCAAAGCCCAGGAATAATTATTCTCCGCCTGGAGGACTGGGGAATATGTAGATTTGTATCATATAAAAGCTTATTAAGGCATAGTAGTCGCTTTCAAGGCAGAATTGAAAATGGGGTTAGGCTCTTAAGAAATTGGGCATCTTTGTCATGGGAGAAGTTCAAGGGACTTCCTGTCATTTTCTTGAGCTGTTTATTGGTATCCACTCACACGTATATGCCTTTTACTATTTTGACAAGTGTGTACTTGCCATGATTTCCTCACCATAAAGCTTAAACTAAGTTTCCCTTTGCCTGATTGATTGCCTCTGCGCTCTCTCTCTCTCTCCCTCACAAATTTTCTTAAATTTTTTCTGAACAGCTGGTTTGAAACATTCCATATTAAGATTATGTGCAAATTTTAAAGTAGTTAAAATCTGAAATCCCATTTTAAAAACTTTCTTGGTCATTGAATGGAACAGAGGTGGTAGCAAGTGGGAAATTGAGATGGGTGGATGAAGATAGTGATGTGGAAGATAGGATTCTGTAGATGTAACCATGTACTTTGAAAAACTATTGAATCCAAGGGCTAAGAGGGGGCCACTAAGACTGGATGTCCAAACAGAAAACAAAAATCAAGACCTCACCATCAATGATCTGTGACTGCATCATCAACAACAACACCTTCATCACCACCACTGCCATCTCCTATGTTGATTCTATTATTTTATTTATTTATGTATCATTATATGTGGGGTTTAGGTTATTGTTCTTGAATTTTTGTAATGATTTTTTTAATAAGATACCTCAAAGGATGGGGTGGGGGGTTAGAGTGGGTTGGAAGCAACCCCACCATCCACTGAAATGGATCCAAATGGCAGAAGGGTAATCAACCACGTGTGCAGTTGAGTTGAGTCGCTTACAAGAGAGAGATGGCATCATAGAATGGGTGTTGCAATAATAGCATGCAGGAGGGTGGAGAAATAATGAGAGAGAGAGAGAGAGAGAGTGGGGGGGAATCCATGGAATGTAGGAATGGTGGGTTTTAATGCTGGTTTGTGTCTGAAATGAAATGAAATGAAGTGGCGAATTTGGCAGTTAAAAAGTGGCATCAAATCAGGGTAGTCAAAACTAAAAACCTATCACCCTTCCAAATACAGTGAAAATAAAGAAGAGATGAATACCGGCTAGAACCTGAGTTAAATTTTTTTTTTCTTTTTTTTTCTTTCTTTCTTTTTTTTTTTTTTTTTTTCGCTTTTGGGGTAGAACCGTAGAGAGGGAGAGGGGGGAGTGACATGGGTGCCATCTTGGGATTTCAATATTTTTTTGTTTTGTCTTTCATTCCTTGCCAAAAACAGTGAAAACTCATGAAAGTGTAGGACTTTTCAGGTCACCTTCTTCAATCAAAGAGGACTGAGAGAGGACCCCCTTTTTTTTCTTTTGTGTGTGCGCGCCCTGTTC

>VvTCP7 (*2000 bp)

TGTGGTCCACTCTGGAATTTAAAGTGGGGTTGTCAGTGTGGGCCCTCCCTCCCTCCGTACTCGAATTGAAGGCTCAGCTCATCTCCAGCTGGGTCCAATTACTCCCCTTTACCCAATCCCCTTTTGCCACGTCATCATCCCTAACTTAATAGGATCCTTTGTTAGGTGCTTCATTTCCTTGTTTTTTACTCTATTTACTAAACAGGTTTTTTTTTTTTATTACATTTCTGATCATTTTTAAAGTAATTATTTGGAGGAAATTTTTATTTTCTTTTTTCAAAAATAAAATAATACCATTTCCAATAATTTTAAATAAATACAAAAATCATCCCGATGATTTAAATTAGTAGAATTTTTTTTTTTTAAAAAAAAAAACTATAGACCGGAGAAGGGTGATGATGAAAGAAAGGGAGGGAAAAACGTGAAATGCATTGGAGCGTGAGGAGGAGGGGAGGGGAGAGGCGCGTGAAGGGGAAGGAGAGGAGGATATAAAGAAGTGAGAGTGAGTGAGTAAGAGAGTGGTGGAAATTGACACCTGGCCAACTGTTTAATTTCGTGTTTTTGCCCCCTTTTTATTCTACACACTATGCCCCCCAAACTCTAACTCAAAACTATTGCTACTCTCATTCCTTCCGCATACATATACATGTATTTTTTTATTTATTCAAAATAAAATAAATGTTGATTTTTGGATACATTTTCTATTTTTCTTAAAAAGAAAACAATTATAATAATTAGTATATAAAATATTTTATACAAAAAAATGTAATTTAATTTATGTTCTCAAAAGTTATTTTTTAAATTTTTTGAATTTGAGGTGATAAAGATTTTCTAATACCTCGAATTTTTTAAAATTTTATCTTTGTAATGTAAAACATTAGGGTTATGTTTGGTTGGCAAGTTGTTTGTAAATTTAATGTGATATTTTTACATTATGCTTGTATTTAGTTTGTAATGTGAATAAAAAATAAAATAAAATAAAATATATATTATTTTTAATATTTAAGATAAAAATGATATTATATTATAATTTTTTCTATTTAAAAAAAATGATATTTTCTTCTACCTTTTCCTCAACTTTATCATCACCACCATCATCATGATCATATTCTGCAAAGTCTCCAAACCAGCCTTTCATTACTCCTATAAATACTGCAAAATCAAACTTACAACTCTCTACTCCAATAGAAACAGTGTTAAAAGAAACACAACATAGATTAGATAAACTTAGTATTAATGATATTAATCAAAATAAGAGTCAAATTCAAATTATAACAGAAGATAAACTAGGAATCGAACAATTCAATATGTCTCCAGATTCAATGTTACACCCCCTCAAAAGTTAATGCAATATCTAATAATTTTGCAAGTAGAAAATATTATTATTCAAAACCCTCTTTTTCATATGTACAATTAGAAGAAATAAATTATCAATTAGATGTTAGTTACAATGATAATAGTTTTTATGAATGAAATATAGATCGAATGAATGAATATCAAATAATTAATTTACTTCATGAAATGACGATGACTGCTAATGTTTAAAGAAACAAGACTAGTAATTCTAATTTTGGTGTTATTGGTGTCTTAGTAGATATCTCATCATCCAAACATAGTCTAAGAGTTTCTTCATTTCTTATAAAAAGTTAACACTATTTTTTATATTGTAAAAATAGAAAACAAAAGTATAACCAAACAACATCTTAATATCAATTTATACTATTTTTGTTTTCTAAAATAATAGTAAATTTTATATAAGATATAACAACTTTTGAAATTTTAAATTAGAAAAGTAATTGTTTTAAAAATTATTTAAAAAAGTTAATTTTAAAAAAAATGAGATAACCTTAAATTTAAATTTTCTTAAAAATATAAGGTAAATTTATATTTTTTTTATACAAAATATTTTACTTTTATATAGAGATTTCTAAAAATAATAATTAAGAAATGTATTTGAACAGACACTTTATAAATTAATATGGGAAGCAAATTTCTTTATT

>VvTCP8 (*2000 bp)

TGTCGTTCTGCTATTAATTTTAATTTCAACTAAAAAATTGATACCCTAAATTTTTTTTATTTAAAAGATAAAAATGGTTGCATATTTAGTTAATTTTGTAATTGAAATTGTGATAATCTAAAATAATTTTAAAAAATAAGAAGTGCCTTCACTTTTAAAAATAATAAAAAAATTTATATTCTACAACTCTCATTTTTAAAAATTTTTAAATATCCATTTAAGTATATGATTGTCTCATTCTTAACTTTTGGATTAGATAGCTTGTTCAAATTACATTAAATTCAAATATTATGAAAACACAATAATTCCACCCCATCACATGGGAAAATTGAGGGCACCATGCCCACTTGCCCAATTATCTTAGATATAAATATATTATCCTTTTCCTTTTTCTTCTTTTTGTGTGGAAAATTTTATTTTTTAATATTTTTTATTTTTATTATTTTTATTTTTTTGGTTTTGTCTTAACTTGCATGCTTTTGTGGCTTTAAATATTAATTCACTTTCTCCCTTTAGCACAATCTGTGGGTACGGTTGAAAAACCTCCATGAGCAGCCTTGCACTTTTCCATCCAAAAATCAGAGCAATATTTAATTCATATTAAAAAATGCTTCTTGTCATGGAGTTTTCTTGGGGATTTGTCACAATTTTTTTATTTTTTTTGCCAGATGTCAGAAGGGGAAGAGATGGACGGCGCACGATGACAGAGTAGGGGTTTGAATTAAAAAAATGTATGAATGAGAGGGAGAGAGCACTCCCTTTCACTAGGAATTGTCATCAATATTTCAATCATATATAGGCAAGTAGTCCCCAAAAGGCATATTTATATATACTATACTTTATTTTTTTCTTTGGAGTAATCAATTTGGCGAGTGGTACACCATGCTGCCTCTTTGGAGGCAAGGTGGAAAGCTTCCAAGTCCTGCCAAACCAAAAGTTAACAATTACAAAGTTGATCATTTTAACCATTATTTTTTCTAGTGTACCTTTAGCAGGGGAATAAAAATTGGTCAAAATAAAGGGAAATATGCAAAGAAAGAGGTGGGAATTTTTGTACCTTTCTTTATGGAAGAATTTTGAATGTCCAAAGTAGTGGAATTTCAAAAAAAGATTTTTCCCTCTTTTTATCTTTTTGCCTTTTCTTCCCCAAATATGATTTTGGGTTTTTCTTCTCTTAAGGGAAATGTGGAATATAATCGGGGAAGTCTTTTCGTGTTCATCACTCCCAAATTTTAAGGTGATACTTATTATAAGATTTTCATATTTTATAAAACTTTTACGATATTAATACTATTACTTTTTAATAATTATGAGTAAGATGTCGACTTTAAGTACAATTTTAAAATAAATGAAAAAGTCAAAAGTTATCCAAACATGCTCAAAAATTTTTAAGGGTGAGACCCTCTATTATCAAGTCAAGTTGTTGGGTATGATTTGTCTAGGACAAGTGACAACCAAAGAAATATACATGGTAAGACAACTTAGATACCATTCCATTACAATAATCAATGCCCATTTGAACTCCTGATCATGTATAATGTTTAGAGGGTTTGACTCATCTCAAGTATCATTACCATATAAATTTGATATTGAATTTATATTATATTAGTTTGTCTTCTAGTGAGTATGGTTTAATAATGTAGTCATACCCAAAAAAAAGCATATTAAAATAGAGGCATTCTATCCATTCAAAATTTGTAACAGTTGAAAAAGCTTTCACTTCTTTGTGTACTCATCTAAGATGAGAAATTTAATGTAAATCTTGAAAAGAAGAGAAAGGAAAAGGGGGTGTGAAGTGTGGGTTTGAAGGGATGGTAAGAGTACAAAATGCTGATGGGGATGCTGAGAAAGCTGGGTGTGGGGGGCTGGGGAGGATGTTTGTACTTTCTTTTTGGTGAAACAATATTTCTCTTTTGTGAAACTAAATAAGTACAGGGGGGGGGAGAAACCACGCAAGCTGTTAGGGGATCATTATCAATGCTTCTCAAGGAGTGGGTGGG

>VvTCP9 (*2000 bp)

TGAAAAAATATATATTTTTTCAAATATTTTTAAATAAAATTTAATATATTTTTTTTATATTTTTTAAAATTAGTTAGTCAATACCTACTGCTCCCACTAAAATATATGTAAGATTACAATGCATTTTTTATGTCTATATGAGATGAACTCATGGAATCAAGCACCATTAATTAGTCCAATTAATGGCTTATAGTACAAACACTGTTTTGCTTAAATGGTGTTCATTGTTCAATTATTTTGTTCTAGATTCTTCCTAATACCTTTAATACCATAAAGATCGAAGATTGTCAACTTTCTACATGCCTTTCTTCCCTAGTCACAAAGTCTAGTGGATGTTAAAAGATAGAAAAATTTGTAAAAAATAAACTCCATGAAAGGAGACTTTTTCTAAAGGTGAACTTCCATTATATATACATAAATAAACTTAATTTGGGATTTTGTTCTTTAATAAGAAGTTAAAATAAATTCAATCATTTTATTATTATATTTGTTTCATTAAATAGGAAATATTAAAAAAAAAATATAATTTTGAGAAGAAATATTTTTTACTTTTCTAATTTTTATTAAATTTTTTTTAAATCAAATCATTATATGCTCTTCTCATCAAATTTAAGGAGTTTATTATTATTATTATTATTATTATTATTATTATTATTATTATTATTAATACTATTTGGCATAGTGAGAAATGTCAAATGGTTTTAAATTGCATTCAATTCTCCACATGAGAATCTTCCATTTGAATTGTGTTGATGATTCTATACTCCGACAACGTCTAGAGTTAATCGAATAATATGATATCTCACATCGAGTAAATCCTTAACCCTTCCCTCTCTTACTAAGACTATAAATTGTTCTTGTGAATTATAACCACTACTAGGTATATAAGCACTGATTTCAAATCCAAAAGTTTATTGTATTTTGACAATTTTATATAAGGTAGAGTTTGGTTTTTTGGAGTGATGGTGGAAAAGTTGATAGATAAGTCACCCTTATTGCCATTCTACAAAACTGTACATGAGATTTTCATATCATACGACTTCTTGTTTAATTCTTTTGAAGTCATTAGATCATTTTTCTCGTTTGAAAATCTCTTTCCCTCTTCCACTCTATCTCGGGGAGTAATTAGAACCAATTCAGTCACATTTTTATGTTCCAATTGAATACTTTCCATTTTTTTTTCAAATCAAAGTCAAAGATTATTCTTTTTACCATATCCTTATATTATCTTATTTTAGATTTTTTCTACATTAAAAGATATTTTGTTTTTCCAAATTTATAACTAAATGTTCTCGATATTTAAAATCTTTGGGTCTATTTGGTATTTATTTTTGAAAAATAGTTTTGTAAAATAGTTTTTTATAACAATTTTTGAAAACTATTTTATGATTTTTGTAAAATAAAAATCAATTGGAAAACTTAAAATGTTTTTAATATTTTAAAAATATTTTTTATATAGTATTTTATTTTTAATCATTTTACATGTTTGGATAATTATTTTTTAAAACAACTCTTGTAACACAAGTGAAAGAAATAAAAAATAATTAGAAGATGTTATCTTAAACTACTTGTTTTTTACTTTTAAAAGTAAAAAATACAAATCAGTTATTGATTACAATATTTTTTATTTTAAAGAACATAAAACTGTTGTAAAAAACAGTTGCTAAACAGAGTGTGGGCCCACAAAATATTTGTACTGTGAAAACAATATATTTTAAATAATGCCCTGAAAATGCTTTTTATCTTTTGAAACAGTTGAGAATGAAAAATAAAAAGACGGTTCTATATTCTTGTGGAAAGGCAAAGGAGAATGTGTGATTAGCTAGCGAACACATTTAAAAAGTGTTTGGCATGCTACCTAAATTGGCTTCAAATAGATTTTGGAAACACTTCTGATATTGAATTGGAAGGTGAAAATGAAAAACACATTTCGGAAAACAATGAAAACAGAAACAAACGATTGTTTATCTGGATCATTGTAGCCATCGCTTTGACCCAAA

>VvTCP10 (*2000 bp)

CCTAGAGACTTATCGCGACTGTTGAAGATACTGACAATGGCGAAGCAATCCTTTCTGTGTTGGCTCTTTTCATCATCTTCTTCCTTTGATCCTTTCTGTTGCAGAATCCGTAGAGAATCTCTATGTAGCTTATGATGAGTCCTTGAGAGATCTAGACTCGGACTCTCCTTCGTACCCTTTTCCAGCTTTTTGAATGTTTGTGGGGGTCGCCTCCACAACTTTGAAACCTGATACTAAGTATCAGAGTAGAGAAAGACAGTAAAATAAGGTGTGCTACAATGAGAAACCATGAACCCACAACTTAAGAAAAGGCATCTAAGGAATGTGACTGCGTCTGAGATAGAGGTGGTGGCCGTGGAGGTGGGGCGGAGGTGAAAGAGTAAAAGGGAGATGGTAGGGTTAGAAAGAGTACTCTTTCTGTGGAGTAACAGAGTCCGTAAATGAGGATGGTCAATGTGGAGATGAAGCACTGCATTCTGCAGTTGCATCGGTGCCGTTGTACGTACCAAAACAGGAACTGACACCCAGTACTCTTACTACTGCTACTACTCAAACACTCACTTTCTCACAGAGCCCTTTCACTTTCTGCTTAACTCAGTTATTTGCAGACAAATCTCTAGCATAATCCCTCACTTCAAGCAGCTTACCAGAGTAATGGGCAGCTGATGGGAGTTGTGCGCGTGGGTTGGAGATTGGGTGGTGAGAAGTGGGAACGTAGGAGGCATGAAGGCGATGTTTGAAGGATTGGAGAGATGGATTCCTCAAACGAAAGGGACGTATAACTCGATTTTCTTGGGAGGAGGTCACCTCTGTCCACTGTGTCGCGCGTGTCTGAGCCTGGCTTCAACAGTGTGTGAGAGAGAGAGCCCACGATCCACCCCTTTTTCATGGATTCCCCTTCCGAGGTCAAGGAGCTTTGATGATGGATTGCTGTGCATGCCTCAGCATTTTCTGTCCTCGAATATGTTTACTGTATTCTAGAATGCCCAAATTTATCCCATTGAGCACACCCACTTAAACATTGCTGTTTTTTGTTTTCAAAATGAAAATTTTAAGACTTGTACTTAATAAATTTGTTATTTATGGCAAAAACAAGTGGGTGAATAAACACATTTTTGAAAACAGACTTATTTATGTCCTTTTCATAACTTGAGTAATTAAAGTTCCATCAATGTCACTTGTATCTATCTTCCCCCTCTGATTTTACCCCCATATCAAGCACATACTTCCCATGGAAAACGTGACGTCCAAGGACTACCAGGTGTTGTTTATGCGATCTTTGCATTTAATTGCCTCCAATGAATAAGCTGGAGGACTCTCTCCCATAAGTGCCGTGTGGTAATTGCCACTTTCCAAGACCTGTAATGCCTCATCCATTGATACGGTTGTCTTTTAGGGTTAAAATTTGCTTCTAATGGACTTTGAAGGGCGTTCCAAGCTAGTGAAAATACCACCAAAATCCAAAGACAATAAGCATATGACAATCCGAATTACTTTCCAACGCGTAAACACTAGGATGAGATTGGGGCTGAGGGAATGGCTCATATTTTGGTTCAACCTCCAAGGCTCCAATTGAGTGGGTGGCGTCAAGTGGCGATGGAAATAGAATGCAGCATGTGCGGGCGGAGCCCACTTGTCATGATTAGCCTGACTCCTTGGTGTTGATGTCCACTTTCTCACTGTAAACACAGCAACACACCAACAGAATACGGATCCTGACTCTCACGTACTACTGCTGGCCCTGCTCATGCCTTTAAGTTGTATTTTGACAGGCGGTGTTTACTTTGCTGATTTGGTTTTCAATGGACAGCTCCATGTACCATTGGGTCGATTTTAAAGTTATTGTAAAATGAAAAGCTCTATGAAGAAGCAAAAGTTCTTCATCGAAGTAAAGATGATGAAGCAAATCTTGTAAGAGTGAATTCTCTTTATATTGCTTTCCTTAATTTTGTATTGTAGAAAGAGACTGCAGGTGTCAAAAGTGGACAAATTGTTTATCT

>VvTCP11 (*2000 bp)

GTTTTTGATGTAGGCTTTAAGGAGAATCTAAGGATCAAATTACAACAGAAAGGTAGTATCATGGAGAGACATCTTTTCTCGAAATGGGCCAAGCTGTGCTTTTCATTAGTATCAATCAAAACTCCTTCAATAACAGGAGCAACATAGAAAAGAGGATTTACAACAAAAAAAAAAAGAAAAAAGAAAGGAAAAAGAGAAAAATCAGCTGCAAATAAAGCAGACAGATACTGTTGGGCTATGATATTTAGTGGGCACAACCTGGGGATAATGGCCTATTTCGTGTCATACAAAGAGGGAAATGGAAAAAATAAGTGCTGTGGTCGGGTAATTCCTTGATCGGTTTAGACTAATGGCATTATAACCTTCATCCTGCTGTAGCTCCCATTGCAATTTGGTGTAGTGGTGGTGTCGTAGAAGTCACCAGTGGATCTGTTTGACTCATGAGTTTAATTAATTGCCTGAGATATCAAATCAAAAATCACAAAGGTGTTTTACTAAGGGTAAAGATGAAAAGCAAGATGATAATTAATTATAAGCATATCCCCAATGACCACTTTTGAGATGGAAGAGGTTTGTGCCATGATATGAGATTACTTCCCAAGCAAGAAATTTGAGGAAAAGATAAATGCTTCCAATGGGTTGACATATGATAATGCAATATTACCAAATAAGGCCACTGAGAAAGAAAATTATTGCACTACCAGTCTCACAAGTTCTTGCTCACCATGGAAATTGGTTCATCCCCTAGCAGACTCAATCCAAAAGGAATCCGGACAAGGGCGAAAGGCGGAAGCAACGGTGAAAATAATAAATATATACAGATGAGTTTNNNNNNNNNNNNNNNNNNNNNNNNNNNNNNNNNNNNNNNNNNNNNNNNNNNNNNNNNNNNNNNNNNNNNNNNNNNNNNNNNNNNNNNNNNNNNNNNNNNNAGAGAACTCACTGAAGTGAAACAAAAGGTGGGTGTCCTTATCTAAGGAAATGACCCACAAGGAAAAAAGGAAAAGAAAAAACGGAAGAAGAAATTATTTGTGGAAATGAAAGCACTGCCCTAACTAAGACTTTAAGGGTTTTATGCTCTCATTATCACCCATCCAAATGTGGTACTTCACCGTCTTTTTTCATAGTTATCGCCTCCTTGAAAGCAACCCCAGAACAACCCTGTTCACCATTTCATGTGGTCCTCCCAATTATGTCAGTCTAAAGAGGTCAGGCTTAGCTAGGCCACAAGCAGAGAGAGAGAGAAAGAGAGAGAGAGAGAGAGAGATAGAAAGAGAGAGAGAGAGAGAGAGAGATGATTCTTGATAAATGGGCACGACGGCATGTATAAATGGAAAAAAAATATCAGGCAAGAGGGTGAGAAAATAAATATGAGTCTGCAATAAGGGCTTTCGATTGTGGATAGGACTATTGCCATATTCGAATAAGATGAGATGTGAGGGGGTGGTAGTTACGGCTATAGGCGATTGGCTAGGGTTTGGAGAAGGTCTTGGGGGATGGCAGTGGGAAGTACTCGCCACAAACCAGAGCAAAACCAAAAACTGTAGCTATGTTAGTTTTTTGTGTTGTGATACAGTGATAGAGAGAGAGAGAGAGAGAGGGGGGCAGCCAGCTTTTTGGTTCTCAAAGAAAAGGTGAACACCCAAAAGCACGCGTCATGGAGTGAACCCAACAACATTCTCACAAGAGACTTGATAGATAGACACAAGGAAGAGAGAGAGAGAGAGAGAGACATAGAGTTGACAGACGGAGAGAGAGCAAGAGATGGAGATGGGTTCTCTTTTAAGTGATAGGGACATATGTTGAAAGAGAAAGAGGGAAGGCCAGTCAAAAGAGCATGGGGTTCCTTGGCAGGCCTCAAAGTATATGCCCTTCCAAGTGAAATAATTGTCCTTCCCTAGGGTTAAACAAGGCCACGTGGAGCCCTTCAAATATGTGATGTATATGCATCTTCCCATTACATTTCACTCACCCCTAACCCCATTTCCCCATTGCTCTTTGAA

>VvTCP12 (*2000 bp)

GAGTTGTCGTTGTGGTAATAAGTGAGTTGGAATGAGTTCATAGTCATCAAGAAACGCGGCAGGGAAGTGTAGGAAGGATGAGGGTGGTTGGTCCTCTCCTGAGTTAGGGTTTTCATCATCGATGTAGCGGAAAGGGTTAGAACGGTTGCTGGAAGGAAACATCCGCAGCGGGTGATGAAGACAAGTGGGAGTCCTTTTAACTCTGCAAGTGATCAGAACAAGTGGCTCAGGAGGAAATGGAAATATGAAGGGAAAAAGAGAGGGGTATTCGCTTTTATGCTGTGAAATAATACAAGTTGAGTAGTACTTACTGATAGATAGCCTGGGCGGCGCCTGAGAAGGAGGTGGTCGTTGTGGTGGTGGTGATGGTGTGGGGCGTGTTAAAGCTGGTGGAGGAATAGGAGGAATATAACACAGAAACTGGTATTTATTACGGACAAGAGCTTTTCTTTTATCTTCATCTTTTGGAGGAAAAGTGAACCGTGGTGCAATTTGGCCCATGACAGAGAGACTAGAGAGAGAGAGAGAGATGCAGTGGAACAGTGAGAGTGAGTTGATACGAAACGCAAATAGTATAATAGTGACACTTCACGTGGGAGCAGGTGAGAGCTTGATAGTAACCCGTCCTTGAGAGAGAGAGAGAGAGAGTCCATTTGAAAATGCCTGCAGTAAGTATAAGGGAGCTATGAGGTGTTGAGGAGGATAGATATGGGCACATCTATGCAAACCTAGACAAGGACAATTATTTCAATGGAAGAAGGCATGTGCATATCAAACCCCCCTTTTCCGCCATGGAAGGCAATGGCTCTTTGACTGGACGACCACTGTCACTTTCTACTCAAAAGAGAAAAACCCCCATGTCTCCTATGTGTATTTTTGCATCCTTAGTTCCTCCAGTTACACCCACCCACCCGCCTGCTTATGCTTATGTGTGATTCCCATATAACCACAAGTAATACTCTCTCTCTCCATCCGCTTTGTCAAAAAAGAGTCAGCACTCATTCGTACCTTTTCCACTGCCATCATGTTCACGAGACCATCTTCAAACCCATAAAATTCTTCATATACCTCACATCCGCATTTCATGTGAAAATGCAATGGTCCTAGACCCAATTCCCCTCTTTCCGGATCCCACCCTACAATTTCCTTTTCCATTTATACATGCCTTTTTATTTTCTTTCCATTTATACATGCCGTCGTTCTTTCCTCAATCTCTCCATCTGTACCTTACCTCCGGGAAGGGCATAACTCCGAGGAACCAACTATACTTTCTAGGACATGATTCCAGTTGAAGGGAACAGTTATAAAAGGTCATGGTTGAGCCCATTAATATATTGCTCAGGGCCTTTTCATTTCCTTGTATAGCGAAATCCCAGACCAAATACTGCAGCTGCATATATTGTTAAACTTTAATTAGGTACTGGCCAGCGGACAATTTAACTACATTGAATTTTCTGGGGAAGTTCTTCCTTCCATGCTTTTCTATATTGGTAATTACCACTTAATGTCAGTTTTCAATACTTGATAGCATGTATGAAATCACATCGTTGGTGATCAGTTAGATTTGTCTATAGATGTATGAATGTTTTATAATAATCTAGAATACCCATTAAGTGATGCTGGAGATGAGTGGAGAACCTTAAAAAAGAATCAGGTGTCTTCTCTTATCATTGAGTAGCTCATGAGATTATTAAGCTCTCATAACCGGCTTTTGAAAGTCAATTCGCTATCAGGTTTACTGTCAAGCAATAATTGTTTCTCAAAAGCTGAACAGAATAGATGGAACATTGTGCACAACAGTCTGTTTCACTCGCACAAATGCTATCTGAATAGATAAAAAGTATATAAAAGACCAATATTTTCATTTCTTTATCCTTTTCTGGTTGTGGTGGAGTTTGATGCTAATTGAACAGTACTAGATCTGAGACGCATACAGCCAGAAGTATCATTTTCAGGAGACAACTGATTAATTGGTAGGTTTGTTCTATGTTTTTTTCCGC

>VvTCP13 (*2000 bp)

CACGCCATATCTAAACTGATGTGAGTTGGTATCAAGAGTTTGATATGATATACCAGTTGAGATATAGAAGTTCCAAAGTCCTTAAATGGCACGAGTCCCTCATTTTGTAAAAGAGTATTGGTGCCAAATGAATGGGGTGGGGCACGGGCACCAGCTAACCAAAAGTGAAGCCGTCCTTTATTTTGTGGTGGGTATAGGTGATAACAGCATAGATTTAAGAAACCCACCCAACAGAGTAGAGCTGAGAGTAAAAGGACATTTGGGAGTAGTTTTCAGAGTGATTATGGAGCATGCATGCGTAGATAAATATTGCACAAAGTAGGGGCCATTTTCATGGAAGAGGTCGGTTTGGTGCGGCTCTACTATTCAAAAAGTGAGGGCGATTAATCAAAATGTGAGCAAAGACTTGTGTCTGGGATATGGGGACCATTTATTTGGCAATAATGGTACCCTTAGAATCAGTCAATCAGAACAGTGTAAACCGACCTTGAGATGCCCACCACTGCTTGTTTAGAGAGAAAGACAGGTGAAAACCGTACAGATTTCTCCTATCCAGCTTTCATCCTGCAAACAGCTAGCCATACCAAAGCCCCTCTCCCTCTTTCAAGTCTATCTCTGGAAGATATGTATTAAAGAACCATTAGCTAAAACACCAAGAACCCCTTATCTGTAACATACTGCTCATGAATATAGGTAACTGTGGGCATTAGGCTTTCGGTACTTTCTCAATTTTTTCGCAAGTAAAAAGCGATAAAGGCTTGGGAGTGTGGCGGTTTTTCCTGCACAACACAGAAGAGAAAAAGGGTAAAGGAATCCGAATTACTTGATTTATGAAATAATTAGGGTTCGAGTATTAGATTTTAGTCAGATGATGCTCATGCAATAGAGCCATAGGAAGATACATTTCTCTCTTTTATCCTTCATTTTATGCAATTCCTTCACTGGAGAGATGAACTCATTGTATGAATTGAATTGTTGAAGCGCTTTGTACGAAGGACAATTTTAACATGTGTCCACAAAGGCATCTCTCTCATTCTCTCAATACTGTTGATACAGCCCAGAGCTTTGGAGTTAGGCTAAGAGATGTTGGATCAGAAAAACCCACAATTTCATTTTTCATCAATTAAACTACAAGGATGATTTAATCCTTGTCTCATTCTTTACATTGCCCTAAGAAAGACTTGCTTTCCAGATCGTTGCTTGTATTGCAGGATGCATGAAAACTACACATAAGCAAATAGGGTGGGAAGCTTGTGATGAATGTATTCACTTGTTGGTTGCTTGTGTGTCCATCTAGAGAAGGCTTTTTCAGAGTAATGCAATCATGTTGAAGTTGGAAGCTATCTACTTCCTTGCTGCTAGAGAGAAGACGTAATCATTTAAGAATGCCATTCAATAAACATAATATGTGATCCTTTTCATCGTTGGGAAAACAATTTTTTTTTTTTTTTGCATAATCTTTTTACTTTGATATGGTTTAATGATGCGAGTCAGAAAAAGGAGAAATTCCATTTCTGGTTTTTACCTGTTAGGACCAACTTGCTCCATGCAAGTCTCTCCGCAGGAAAGTAGTGTGGTCCTCTTTTGAATTTCTTCTTAGTGGATGTTTTAAAGATGACACAAATTGGTCCCTCATCTATGAGAGAGAGCCTCTCATTCTCCCAAGTAGCCATGTACACATCAACACGACAATGCTCTTAGACATAGACCACAACTTTCAGAACAATGAATGGACTTACATATCTTAGATGATTTCTTTTTTGTCTCTGAAACTTCTCTCATCAGAAACCCTGATTGTGTATTATCTTCCATTCCTACATTTGATGATGATCCCTTTTTTTATCTTGCTAGACAGTTCTATCAATTTATTGATTAGGTGAAGTTATCTAACTTATAATACTTCACAAGGGTTTAAATGACTGATCAAATTAAGTCTAAAATTAGTATAGATAACAAATAAGAAATTGGGTAAAATAGATGGATTGAAACTTCAGCTGATA

>VvTCP14 (*2000 bp)

TCAAGTAATCATTTCCTTTTGCCTTTGGCCTTTGCCTTTGTTCTGCACCTTTGCTGGGGGACTTTGGGATTCCTGATTGCCTTTGGCCTTCCCTTAGGGTTAGGGCTTTTTCTTATCATCTATATCCTTCTTCCTTCTACGGTCGCCTTCCATCTCATGTTCAAATTCCTCAAAACTAGATTCAGTCTCAGTATGCCCAAGAAAATTACATATGATATACTACTTTTTTCACATTTATTATTCATTAAGGTCTCATTTATCTTGGGCAAAGGCTTAAATTAGTGAGCCTGGGGTGGTCTTATAAAGGCAATTAGATCATAATTGACTTGTTTTTGTACATAAGCCATACTCTTGTTCTTCTAAGATGTGGTTAAAGACAGGATCGATCATTAATGATCACCGGTCATAAGTGCTTATAAGCTCCAAAAATTAGAACATTCCACCCCAGAAGCACTGTGTGCACTTGTGCATGAGTGGCATGTCGCAATTTCTTCCATAATTGATGTTTACATAAGTAAATTCTTTTGACAATAAGGTTGATGGAAGATTAATGATAGCATTTTGGCCGTTTTCGTGCTTTTGGGGTAGAGAAAGATGATCTTTTGGAGTGCAGCATCCCATGAAGGACAAGAGCAAAAGGGTGCACATGAATTACTATGTGTGTATTTTATGATCAATTATCACAAGCTCTGGATACCTCGTTATGGTGGAAAGAAATACATTTTTTAAAACTTTACCAACCCGAAAAGTATATACCGTTTTGTAAGAGCCAAGTTTCACGTGGAATTGTGCTTTAATCAACATGTATAAATATGTGCCATAGTCGAGTAGTGCCCAAAATTTCGGGGCAGTGGCGAATGTCCTAGCCATGGCCTACAGCTGAAGGTGAAAGCTCGACAAAGTACGTTGGTAAACATGATTTGAGGATAGAATGAAGTTTGATCAGCTCCCTGGTAGCTTAGCCCAATTGCAACCATCAATTGTTGCAAGCATAATGTCATTTTCAAGCCCCACTTCACGAATATAGGTCTATCAGAAATTAATGGTTTGCCTCCCCCACAAAATGATACGATGCATGTTGTAAAAAACATTCAATTAATAAAAGATGGAGCTCTGGAGGATGTTACAACCCTATCCACAACTCCGGAAATTTTGATTTCATGAATCACCCCCATACCAGAATTATTCATGTTAAGGTGCACGCTTAGGGATTGATCCATCTTTGTGAAGCATTTACTGCTTGCGTACAAAAACATGTGGATCATTTGATGGGAATATAGTGAGGTGCCACAAATCAGGAGATAGCCCTAGTTGCAGTATCCTAGCTGTGACAGCATTGTAGACCGAGTCCCAGGAAGTGTAGGACCGAGCATTAGGCCATGTGCTGCTTCTTTTCGGACCACACCCCTTGCCCACCCTTGTCACTGAACAAGACGCTTCACCATCAAACTCTAACATCTCCCCGAATTAGAACCGCCGACATAGGAAAAATCTTTCTTCAACCTCTTCTAACCTACGATTTTTGTTTCTAACCCATGATTATGTTCCAAACCACAAATTGTGATATATTCCATGGAGTTGAGTGAAATAATGTAGTTGAGGCAATATTAGTCATAGTTGATGACTGGCGGTTAGGTGACTGACTGCGTGGTTGCAGGGTTGTCTGGTGCAGCCACTCCGGGGACTAGCAATTGACTTGAGTTTTTTCAATTATGGAGGAGCTGAGCCTGAAATTTCTGGCCCAACGCCTGTGTTTGTCTCCATTCACATCAAGTCAACTGGATTAATAAATGTGGTTGGCTAAGGGTTGCAGCCGTGTACCTCTAGCTTCGCTAATTTTGCATGATACCTTTTCCACCACAATAAAATAAAATCAATAATATCAATTAATAATACAACCAAAAAAAAAAAAAAAAAAGGGGCAAATATGGTATAAAAAAATATACCATTTCAAGGGTTAATAGTCAATACATGAGTGGTTTAGTTTTGAATATACAATT

>VvTCP15 (*2000 bp)

GATCATATAAAAATTTATTTTTATTTTTATTTTTGCCCTCAAATCGTGTAGGAGGACATGATCTGTAGATGACCCTCGACTCAATCACTTTGGTGGGTCTCTTTCTCTTTCTCTTTCTCTCTCTTGTAGAATGTATGGGAATTTCCTTTGAGATGGGAGCTGGCAGCTGAGGTGGTTTTGGCTTTGGGAGGATAAGGAAGGGAGGAGGAGGGCAAGCTTTCAGCAACAAATATCATGAGTGGGAATTATATGGTTGGCCTATGAAAAGCTTTGTATTTTTGAAAAATCCTTGCTTTTCCGACCCTCTTTTGGTCATTTGGGTTTTTCACTATTTTCTTAATATTAATATTGTTATTCTCCCTTTTTTTTTTTTTGAAAATTTTGGAGTCCTTTAATTGTGGTTGGACACTGGCTTGATTCAGACTCCACGTGCGGTGCTCTTTATCATTCAATCCGTAAAGACCCTGACCCTGGTGGGCAAAGCCCTAAGGTGAAGGGGGCGGGACCCAGTCCCCCAAGTCAAAAAACTGAAAGCCCGGTAAAGAAAAGATGATCAAGTGTGGCTCCCATCTCCTTCTTGTAATCATTGCGTTTCGCCTTTCCTCTAATCCCCTGGGCTTTGGGTTACCCACCCACTTGGATTTTTGATCTCTGTTAATTAATGTATACCCCCATTTCCATTAATTTATGGACCACAATACATAACCATGGGCATCACAATCTCATTGATGTTAACGTTGTCACATTTTACCACCTCCCCTAAAGTTAGGGGTTGAGATTTCAACTTTTTAGGGCGCCCATGGCTGGGCAAATGCTCGTACCTTGTCTCCTCCCTTTTATCATATTGAGGGTCCTCTTCCTTACCCATCACTGAAACACACCTAGGCCCTAGCCCTCCTCGCCTACTTGTGTGCCACCAAATCAACACACTTCTTCCATTCCATGTTTCCCTCCTAGGAGAATCTCATACTAATGCTACAATATATTTTTTCTTAATTGATTGCTACATAAATATGATCCCACACCTGTTTTTAATTCATTTGGGGTTTGAAAAAAGTGGATCCAACATGATATAATCACAATCACATGGGTTTACAGATTAGAATTTGATTTATAGATTTTAAGAACCAAAAAGAAAAAAATAAAATCAGAGAGACATAGTGATGAGCATAAATTATAAATAGCATGTGAGTGCGGCAATGTGTGTGGGAGAGGGGTACACACATACACAGTACATAATGAAAGTTGAACAGAGCAAATGCACATGCATCGCATGTGGCAAAGGGCCCCTAGTGTAACTTAAAAAGGAAAAAAGAGGACGCATTACAATTAAGTGACAAAGTTCAAAAAAAAAAAAAAAAAAATTTCCAACAACCAACATACCTAATCCATGAACAAACTATTTCAACCACTAATCATATGTAAATCCCAGTCAAGCCCCCCTCAGTCTCAACTTGATTATCTTAAAAAGCTTCGGCCTCTTCTTCCCCTCCAATTAAATTAGACTTAAGTTTCGACATGTACTATAAACCTCGTCCTGTCATTTCCTACACATTAGCACCCAGTTCGATTAGTACTAAACTTGATCTAGATGACACTTAAAGTCATGTCATTACCATTCCAAATGTACTTTTCAGTTTTCACCACCCACCAGGCCACCACCACCACCACCACCTTGTCTCACTCACTCAACTACACCTACATTCTATTCTGTTCCATTCTTTTCTTTTCATCAGTTCTTCCTTTTTCTCATTCGTTCCCTCCCCCTCACTGTAAATAATAATAATAATTCTAAGACCAACCCAATTATATGAAGGTCCTATCTCATGCCTGTCACTCAAGCCAACGACACAATCACGTTCCATTAGAACCGCAAGCCCACTCACAATTATTGTGCATATCAACAGCAACCCCTCCAATGCTACTGTATCTTTGGTGAATTAAAATCCTCCTTAATTTATTTTTAATTTCTTTTTAATCACCATGTGTAAGTTTATTCT

>VvTCP16 (*2000 bp)

TTTATTATTGATTATAGAAATTTATTTATTTTTTAAATTATTTTTTACTATTTACTTTATTTGGAAAATGAGGTAAATAAATAAAGGTAAAAGGGTTAAAAAGGGAAAGTTAATGGGAACAGAAAGAAATGAGGATCAGCATTGAATTGAATTAAGCGAGGAGATTTTGATAATCTCACCCGAAGTTGACAGTTTCTCACCAATTACAACGTGGATTTCCCCCCACTTTTTCTAACCACTACGTGACAATATCTATTTTTTTTTTTTTGCCCTTTTTCCTTTTTTGAATTTTGAGATATGATAGAACCTTAAAAGTGGGTAGGTTTGGGTTGCCCTTTTCCAAATTCTTAAAAGTCATATTTTTAAATTTTGGTCATAAAAATCTTTAAAATGATAAAAAAAGTTATTATCTTAATGACTAAAAAAAATATGAAATATTAATTATTTTTAAATAATTATTAGAAATTTATATTTTATAAATTTGAGTTCTTATTTTACGATTTTTTTATTTGAGTATATGAAAATATATTTTTCTAAAATTTTTTTTAGTATTTCTAAATTTTCATATAAATTTTAAATAAAGAAAATCATCAAAATCATAAAAAATTAAATTTATGAAAAAAAAATTAAAAAAAGGATTAAAATTATTTTTATAAAAATAAAATTAGAAAAAAGGTCATGGGTGGGGTCTCTTTCAAGAATCCCTTTTAATATGGAGAACCAAACAAAGGGTCTAGAATGTGATGTGTGAATAGTAAATTCCACTATGGTCACTGTCCATTCCAGAGGGGAAGAGAAATCAGGACTTTTTTTTTTTTATAATAATAATTTATTGGATTAAAATATTAAATATTTATTATTATATTAGGATGTGGGTTGGTTAATTTTTTTTAAAATAATTTGTGATGATTATCTGTTAGAAAAAAAATAAAAAAAATAAAGCATTTGATTGGAGAAAGCAGGCCCACAAGTGGAATTGGCTGGATGATTTGGTGGCTGTTAAATTAATTAATTAATTAATTATAATATATTTGTTGTTTAAAATTCCATAATTAAATAATAGGAAGGGCTTTTTGGGCAACCTCTATTTTTTTTAATTTAATTTATTTAATTTTGGGTTTCTTCTGACAATGAGTTGACAAAATTGTCCTTTTTTTAAACCATTGGGTAAAAGCCTCCAACAAACTTTATGCCAAAAAGACATCATCCATTATAGACTTTTTAGTAAATTTAACTGCCCTATTCAAAGTAATTATATTAATTTAATTTTTAAGAAAATATAATATTCAAAAATTTCTTAATTATACAATCCTTTAATAAAAATTAGGGAATATTTTGAAAAATTAATAAAAAATCTTCCATAATAATTACAAATATGACGTACATAAAAAAAAAAAATTAAGGGTATGAATAATAATAAAAATATGAACCCAAATTATATATCGGGAATGTTTTTTAAAATCACTGTCTAACGTACTATAAAATCGAATTTCGATTGCTCTTAGGATCTTTTTACAAATTTGATTATGGTCAAATTAAGTAAAACCCAAAAAGGGAAAGGGAGAAGGGGAAAAAATAAAAAATAAAAAAATAAGAAGAAGAAGAAGAAGAAGAAAGAATAATAATAATAAAGAAAGAAGAAGAAAGTGTGGGGTTGGGTTGGGATGAGCTGTCATACTGCATTAGATGCTTTATCTGATGCTGACCTGTGCTGTCTATCTGCTACCCTGGACTCACCCTTATTTTTACCACTAAACCCATTTCTCATATCTTTTTAGAAAGAGAAACAGCATGGAATTAAAATTAAAAATTAGAAAAAAAAAAAAAAAAAAGGGAAAGAAATTGACACTGTTGAATTAGGGTTTACCAGAGAAAGAAGAAAAGAAAAACCCACTTCTGCATATTCTGGGTCTGCAAACTGCACACCAACAGTCATATAGAGAGGATAGAGGATTCAGCTCACACAGAAAAAAAATAAAATAAAATAAAAAGAAAAAAAA

>VvTCP17 (*2000 bp)

GGTGGTTTAGGCCTCTTTGACGACGGCGGCGGCGATCCAAGCCGGAGAATCTTGGGGTTTCATCATCAGCTGTATCCTCATCTAGGAACCGGCGGTGCAACCTCGTCTTCGCTAACAAAGCCTGCGGCGCCCGGGCCTCAAGATCACGAACAGGGTTCTTCTCCGGCGGCGACGCACTGTTCAGCTCGACCTGCTCCGGCCATGTGGGCAGTTGCACCTGCCACCAGCAACGGCGGCAGCGCTTTCTGGATGCTGCCGGTGACTACCAGCGTTAGCACCTCAACCGCCGGAGTTGGGGCCTCGGAGCCTCAGATTTGGCCGTTTGCAACGGGCGGAGGGCAGTACAGAGTGAATTTCTCGGGTGGGGTGAGTCCAATCCAACTGGGTCCAATGGTTCTGCAACAGCCACAAGGGTCTCAGCAACTGGGATTGGGACTATCAGATATCAACATGGGAATGCTGAATGCTTATAACAGCAGTAGGGTTGATTTGGGGATGAATTTGGAACAGCATCAGCATCAGAACCAGCCTCCTCAAGGTAGTGAAGACAGTGGAGATGAAGATGCTGCTGAGTCTCAATGATTTCAAGTTAATTGCTTCTTTCTTTCGGCTTTGCTAGAGCTTTTGGGAGTGGTTTATCAGCCATGACTCCTAGCTTTTTGTGTATGTCCTTTGCTTTTGTAGCAGTAGCTATTATTGATTGTTGACTGCATTAAAGAGTTGAAGGAATGGAATTTCTTTTCACTTCAAATGCCTCTAATCACATCATTTCCAACCCGGATAATTGATCAATAATATCAACAATTTGGATTTTGTTTGTACTTAAAGAAGCTGAGAACACCCGGGTGCTCAATCTAAAATTGAGTTTATGCCGAATAATCACTAGTAAAAAAAAATTAATCTTTGAGGTGACGCCTAAACATTTTGACTTATTGACACTTCTAGACGGTGTTCTTACTACCATTGTCAAGACAGTTAATAAGCAATACTTAATAAGCATCATCATTAAATTATAATCACTTTTCTATGATCAAAGATAATAGTATCTAGTCTATGGGTTCATGTTTCTAGCTCTTTATTGAAAATTTCTAAAGTAACAAAGTTGCTTTATATTCACATTTTGCATCTACCTAGTCGGTGGCGATACTCTGAATTATTAGGAAAAATGTTCTATTTGTATCAAAAACTCGTTTTAAACCTATTTTCTAAAAACAAAAAAATGTTTAGCCTATTATTATGTTAAAAATAATAATTTAAACCTATTTATTAGCAAAAAAAGGTGATTTCAAACTATTTACATCTTTAGGAATGTAATTTAAATTTATTTTCATTTTTAAGGAAATCAATTTATTCATCTTTTCATAAAATAACTTCAGCTCTTTCTAATTTTCAGAAAAATGATTTTTAACTTTTCATATTTTTGGGAATGATTTCACCCTTTTGATTTTCAAAAGAATCGGTTTTTAATTTTTATATTTAGAAAAAAATGATTTCAGTGTTGAAAAACAACGATTTCAATTTTTATTTATAGAAAATACTTTCGTTTTTTATTAACAAAAAATGCCCTTGTATAGAAAAACGGTTTTGATTGTTTATGCTTTGACCTTTTCTAGATGGTGCCAAACCAAGCACCACATTTCAACTCTAAAACAAAAAAGGATGGTTAGAAGAGCTTTGCGGGGTGGGGGATGGTTGGTCAATCAGGCCAAACCTCTATTTAATGATTAAGTCAATCATGACTAGAAGATTAGAAGTGCTTATCATGGTCTGGATGTTTCCAGATCAAATCCTGGTGGTGAAGAAGGTTGTTGAAGTTGTTGAAATTCTTGCAAACTTGTTGATCTTCCTCCTGATGACTCTACCTTGAAGCTAGGTGTTAGGAAACCCCAAATTGCTCCATTGCTAAGCCCTAAATTACTAAGGTGCATTTAATCCTATCTTAAGCTTTCTAGATCTAAGCACAATGGAAACTTTGGTATTCAAAAACCAAAAAAAAAAAC
